# SMN deficiency inhibits endochondral ossification via promoting TRAF6-induced ubiquitination degradation of YBX1 in spinal muscular atrophy

**DOI:** 10.1038/s41413-025-00473-6

**Published:** 2025-12-01

**Authors:** Zijie Zhou, Xinbin Fan, Taiyang Xiang, Yinxuan Suo, Xiaoyan Shi, Yaoyao Li, Yimin Hua, Lei Sheng, Xiaozhong Zhou

**Affiliations:** 1 Department of Orthopedics, The Second Affiliated Hospital of Soochow University, Suzhou, Jiangsu China; 2 Department of Orthopedics, Gongli Hospital of Shanghai Pudong New Area, Shanghai, China; 3 Department of Neurology, Children’s Hospital of Soochow University, Suzhou, Jiangsu China; 4 Jiangsu Key Laboratory for Molecular and Medical Biotechnology, Nanjing Normal University College of Life Sciences, Nanjing, Jiangsu China

**Keywords:** Bone, Bone quality and biomechanics

## Abstract

Survival of motor neuron (SMN) protein encoded by *SMN1* gene, is the essential and ubiquitously expressed protein in all tissues. Prior studies demonstrated that SMN deficiency impaired bone development, but the underlying mechanism of abnormal endochondral ossification remains obscure. Here, we showed SMN is involved in hypertrophic chondrocytes differentiation through regulating RNA splicing and protein degradation via analyzing single cell RNA-sequencing data of hypertrophic chondrocytes. Of note, SMN loss induced dwarfism and delayed endochondral ossification in *Smn1* depletion-severe spinal muscular atrophy (SMA) mouse model and *Smn1* chondrocyte conditional knockdown mouse. Histological analysis revealed that SMN deficiency expanded the zone of hypertrophic chondrocytes in the growth plates, but delayed turnover from hypertrophic to ossification zone. Widespread changes in endochondral ossification related gene expression and alternative splicing profiles were identified via RNA sequencing of growth plate cartilages from SMA mice on postnatal day 4. Importantly, Mass spectrometry-based proteomics analysis elucidated Y-box-binding protein 1 (YBX1) as a vital SMN-binding factor, was decreased in SMA mice. YBX1 knockdown reproduced the aberrant gene expression and splicing changes observed in SMA growth plate cartilages. Comparing the binding proteins of SMN and YBX1 revealed TNF receptor-associated factor 6 (TRAF6), which promoted ubiquitination degradation of YBX1. By conditionally deleting *Smn1* in chondrocytes of WT mice and overexpressing *Smn1* in chondrocytes of SMA mice, we proved that SMN expression in chondrocytes is critical for hypertrophic chondrocyte-mediated endochondral ossification. Collectively, these results demonstrate that SMN deficiency contributes to rapid systemic bone dysplasia syndrome by promoting TRAF6-induced ubiquitination degradation of YBX1 in growth plate cartilages of SMA mice.

## Introduction

In vertebrates, long bones and vertebrae are formed through endochondral ossification. Chondrocyte differentiation in the growth plate is involved in endochondral ossification and is a major factor for bone growth. Impaired endochondral ossification leads to severe skeletal dysplasia. Apart from progressive atrophy and weakness of proximal voluntary muscles, spinal muscular atrophy (SMA), a fatal genetic disorder, also presents skeletal abnormalities, including bone growth defects, long bone fractures, osteopenia, and scoliosis.^1–3^ To date, several effective therapeutics including antisense oligonucleotides Nusinersen, small molecular drug Risdiplam, and gene therapy Onasemnogene abeparvovec have been approved for SMA patients, and significantly extend life-span especially for severe patients.^4^ This prompts us to further consider how to better improve living quality of SMA patients. Bone, as a critical factor determining quality of life, exerts important roles in supporting motor function and protecting soft tissues like muscles. Considering that bone development abnormalities may be attributed to impaired endochondral ossification, we further explore the mechanisms underlying endochondral ossification defects in SMA patients.

SMA is caused by homozygous mutations or deletions of survival of motor neuron 1 (*SMN1*) which encodes the essential and ubiquitously expressed protein SMN located in both nucleus and cytoplasm.^5^ The most well-known role of SMN is in the assembly of the spliceosomal snRNPs, which are required for the catalysis of intron removal during pre-mRNA splicing.^6^ Widespread defects in splicing have been reported in various tissues such as spinal cord, muscle, liver and brain in SMA mice where it was observed that the level of aberrant splicing increases with disease progression.^7–9^ SMN also plays important roles in multiple fundamental cellular homeostatic pathways including mRNA trafficking, cytoskeletal dynamics, and ubiquitin-proteasome system.^10–14^ All animal species possess only one *Smn1* gene, and knockout of *Smn1* is embryonic lethal, whereas humans harbor a paralogous backup *SMN2* gene, which expresses the same SMN protein.^5,15^ However, two crucial nucleotide transitions in *SMN2*, including C6T in exon 7 and a lesser degree G-44A in intron 6, induce skipping of exon 7 in approximately 90% of *SMN2* transcripts and consequently produce about 10% full-length SMN protein.^16–18^ The small amount of full-length SMN protein expressed by *SMN2* is not sufficient to fully rescue the loss of *SMN1*, but it is essential for the survival of SMA patients.

Accumulating evidence indicated that bone structural impairments and developmental defects in SMA patients and mouse models are primarily attributable to cell-autonomous mechanisms. Hensel et al. demonstrated that in SMA mice, bone growth defects occur before mineralization defects, even earlier than the onset of neuromuscular symptoms, indicating that bone growth defects are partially independent of neuromuscular degeneration.^19^ Moreover, Conditional knockout of *Smn1* in mouse mesenchymal progenitors led to bone development defects and impaired growth plate homeostasis which inhibited neuromuscular junction, emphasizing the importance of promoting bone growth in treating SMA.^20^ And this study also elucidated that SMN protein loss reduced local insulin-like growth factor in chondrocytes, accounting for inhibited bone growth in mice.^20^ Although defects in endochondral ossification have been observed in SMA, no comprehensive studies have yet investigated the impairment of this process at cellular and molecular levels.

In the present study, we showed that SMN protein is involved in hypertrophic chondrocytes differentiation through regulating RNA splicing and protein degradation via analyzing single cell RNA-sequencing (scRNA-seq) data of hypertrophic chondrocytes, which are characterized by the marker gene collagen X type I (*Col10a1*). SMN deficiency impaired endochondral ossification, and suppressed the differentiation of hypertrophic chondrocytes and the turnover from hypertrophic zone (HZ) to ossification zone in growth plates in SMA and chondrocytes-specific deletion of *Smn1* mice. Using mass spectrometry and GO enrichment analysis, splicing factor Y-box-binding protein 1 (YBX1) was identified as a crucial SMN-binding protein and regulated mis-splicing of endochondral ossification related genes in growth plate cartilages of SMA mice. Mechanistically, SMN ablation increased the interaction between YBX1 and TNF receptor-associated factor 6 (TRAF6) to promote YBX1 ubiquitination degradation. Finally, after treatment with antisense oligonucleotide 10–29 (ASO10-29), a therapeutic antisense oligonucleotide to increase SMN level, we observed a recovery of chondrocyte pathology, YBX1 protein level, and alternative splicing events. Together, these results demonstrate that SMN deficiency inhibits endochondral ossification via increasing TRAF6-induced ubiquitination degradation of YBX1 in growth plate cartilages of SMA mice.

## Results

### SMN regulates the progression of endochondral ossification

**Fig. 1:**
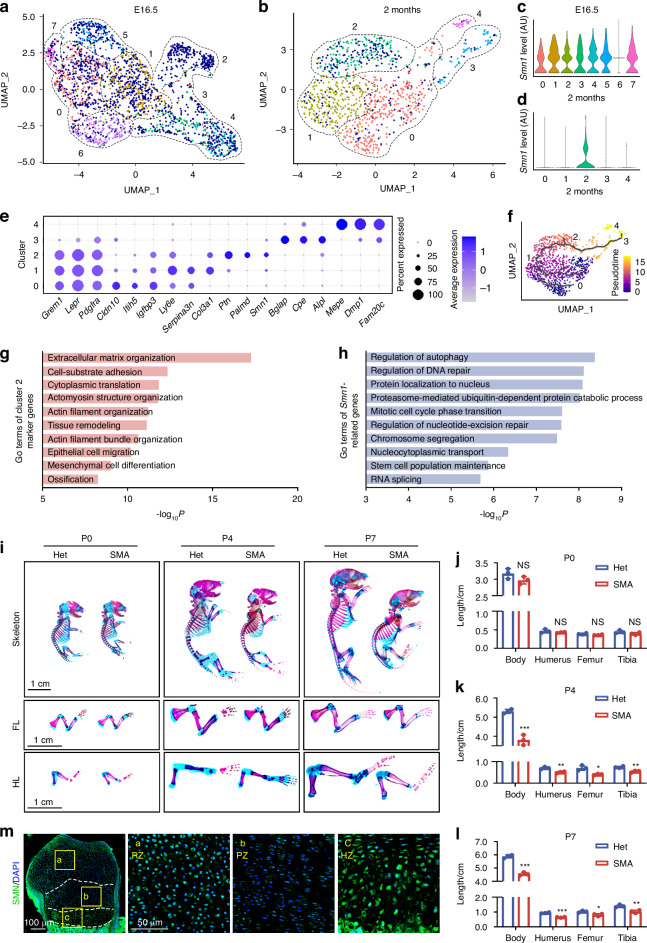
SMN deficiency retards bone development during postnatal growth. **a**, **b** Uniform manifold approximation and projection (UMAP) visualization of tdTomato^+^ cells isolated from tibia and femur of *Col10a1Cre; Rosa26*^*fs-tdTomato*^ mice at E16.5 (**a**) and two months of age (**b**), respectively. Each cell on the UMAP is colored according to its cluster, with *Smn1*^*+*^ cells specifically highlighted in dark blue. Violin plots showing the expression levels of *Smn1* in each cell cluster at E16.5 (**c**) or 2 months (**d**). **e** Dot plots displaying average expression of marker genes across different tdTomato^+^ cell clusters at 2 months. Dot size indicates the percentage of cells expressing the corresponding genes. **f** UMAP visualization of Monocle 3 trajectory analysis throughout all tdTomato^+^ cells at 2 months. **g** GO analysis of marker genes for cluster 2 at 2 months, identified using Seurat 5 with a minimum expression percentage of 0.25 and a log fold change threshold of 0.25. **h** GO analysis of *Smn1*-related genes with a Spearman correlation coefficient (ρ) greater than 0.3, a *P*-value less than 0.05, and a positive cell count exceeding 50. **i** Alcian blue and alizarin red double staining of skeletons, forelimbs (FL), and hindlimbs (HL) from SMA and Het mice at P0, P4, and P7. Histograms showing length of bodies, humerus, femurs, and tibias of SMA and Het mice at P0 (**j**), P4 (**k**), and P7 (**l**) (*n* = 3 per group). *P*-value was derived from Wilcoxon rank-sum test. **m** Immunofluorescence staining of SMN in growth plates of Het mice at P4. Data are presented as the mean ± SD. ^*^P < 0.05, ^**^P < 0.01, ^***^P < 0.001

To investigate the role of SMN in bone development, we analyzed scRNA-seq data from two publicly available datasets (GSE190616 and GSE179148).^21^ These datasets included 2 273 and 1 068 tdTomato^+^ cells at E16.5 and two months of age respectively, which are isolated from tibia and femur of a hypertrophic chondrocyte lineage tracing mouse model (*Col10a1Cre; Rosa26*^*fs-tdTomato*^). We detected widespread and uniform distribution of *Smn1* expression in tdTomato^+^ cells at E16.5, which primarily consists of hypertrophic chondrocytes and their descendants (Fig. 1a, c), only Cluster 2 exhibited high *Smn1* expression at 2 months (Fig. 1b, d). We then explored the cellular composition at 2 months. Clusters 0–2 were identified as skeletal stem and progenitor cells (SSPCs) based on their expression of canonical markers such as *Grem1*, *Lepr*, and *Pdgfra*. Clusters 3 and 4 were characterized by high expression of *Bglap*, *Cpe*, and *Alpl*, and *Mepe*, *Dmp1*, and *Fam20c*, respectively, and were thus annotated as osteoblasts and osteocytes (Fig. 1e). Trajectory analysis using Monocle 3 revealed that Clusters 0 and 1 (SSPCs) reside at the root of the pseudotime trajectory and give rise to Cluster 2, which subsequently differentiates into osteoblasts (Cluster 3) and osteocytes (Cluster 4) (Fig. 1f). These results suggest that *Col10a1*^+^ hypertrophic chondrocytes have already transitioned into SSPCs by 2 months of age and continue to differentiate along the osteogenic lineage. During this transfer, Cluster 2 appears to serve as a pivotal intermediate population, positioned between early SSPCs and mature osteolineage cells. Notably, Cluster 2 exhibits specific expression of *Smn1*, along with *Ptn* and *Palmd*, two genes implicated in osteogenic differentiation and calcification.^22,23^ To highlight the unique molecular profile of Cluster 2, we designated it as SSPC^*Ptn::Palmd::Smn1*^.

GO enrichment analysis of the marker genes for Cluster 0 and Cluster 1 revealed enrichment for cellular homeostasis (Fig. S1a) and vascular development (Fig. S1b), respectively. Moreover, marker genes of SSPC^*Ptn::Palmd::Smn1*^ showed strong correlation with extracellular matrix organization, actin cytoskeleton, mesenchymal cell differentiation, and ossification, suggesting that SMN may be important for the differentiation of hypertrophic chondrocytes into bone (Fig. 1g). To investigate how SMN regulates endochondral ossification, we calculated the Spearman correlation coefficients (ρ) and *P*-values between *Smn1* and other 14 715 genes. We identified 549 genes with ρ value greater than 0.3, *P*-value less than 0.05, and positive cell count exceeding 50. GO enrichment analysis showed that these genes were involved in autophagy, DNA repair, protein localization, proteasome-mediated ubiquitin-dependent protein catabolic process, and RNA splicing (Fig. 1h). Thus, SMN protein may influence the progression of endochondral ossification mediated by hypertrophic chondrocytes, potentially through its involvement in various biological processes, such as ubiquitin-dependent protein degradation and RNA splicing.

### SMN deficiency retards bone development during postnatal growth

Consistent with previous study,^24^ we utilized a severe SMA mouse model with a mean lifespan of 10 days (Fig. S2a). Considering that the first symptom appears at P5 with impaired motor function,^19^ we selected three time points: P0, P4, and P7, representing neonatal, pre-symptomatic, and symptomatic stages, respectively. No significant difference was observed in weight and body size of SMA mice compared to the Het group from P0 until P4 (Fig. S2b, c). Alcian blue and alizarin red double staining revealed the reduced length of body, humerus, femur, and tibia in SMA mice starting from P4 (Fig. 1i–l). Longitudinal limb bone development is driven by endochondral ossification controlled by the growth plates, which are divided into the resting zone (RZ), proliferative zone (PZ), and HZ based on morphology and function.^25^ Immunofluorescence staining revealed widespread expression of SMN protein in growth plate cartilage, with particularly strong signals in HZ (Fig. 1m). To validate the reduction of SMN protein, we performed Western blot analysis and confirmed a significant decrease in SMN expression (~50%) in SMA growth plate cartilage across all three timepoints (Fig. S2d). Together with the transcriptional findings, these results support the notion that SMN is essential for growth plate–mediated endochondral ossification. SMN deficiency is therefore likely to impair bone development at the pre-symptomatic stage.

### SMN deficiency induces abnormal growth plate anatomy

**Fig. 2:**
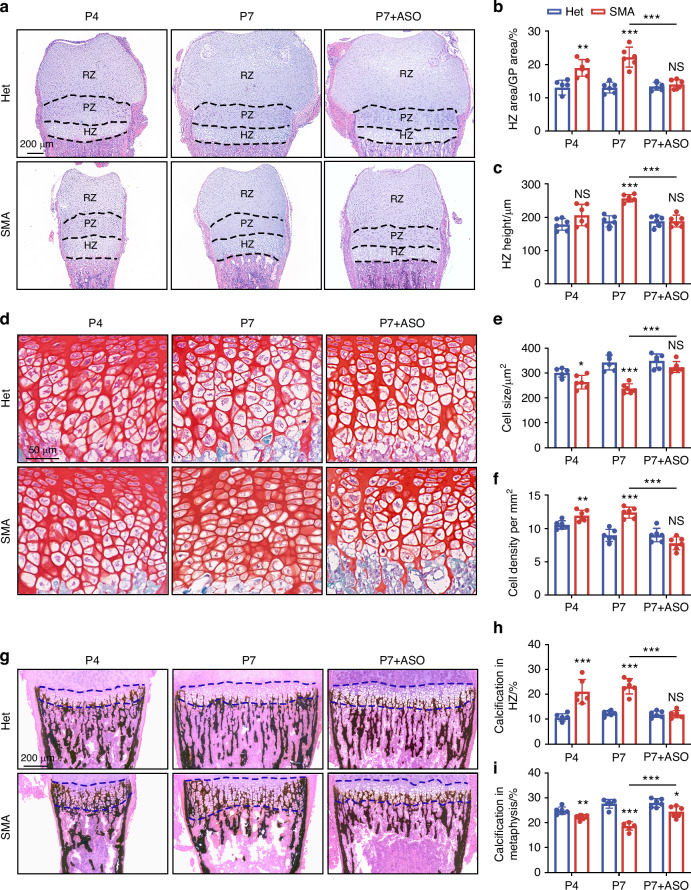
SMN deficiency induces abnormal growth plate anatomy. **a**–**c** HE staining of growth plates from SMA and Het mice at P4, P7, or after ASO treatment (**a**). Histograms show ratio of HZ area (**b**) and HZ height (**c**) in growth plates (*n* = 6 per group). *P*-value was derived from two-tailed unpaired Student’s *t* test. **d**–**f** SF staining of hypertrophic chondrocytes in HZ of SMA and Het mice at P4, P7, or after ASO treatment (**d**). Histograms show cell size (**e**) and cell density (**f**) of hypertrophic chondrocytes in HZ (*n* = 6 per group). *P*-value was derived from two-tailed unpaired Student’s *t* test. **g**–**i** VK staining of HZ and metaphysis from SMA and Het mice at P4, P7, or after ASO treatment (**g**). Histograms show ratio of calcification in HZ (**h**) and metaphysis (**i**) (*n* = 6 per group). *P*-value was derived from two-tailed unpaired Student’s *t* test. Data are presented as the mean ± SD. ^*^P < 0.05, ^**^P < 0.01, ^***^P < 0.001

Growth plate cartilages of SMA and Het mice were stained with hematoxylin-eosin (HE) at P0, P4 and P7 (Figs. 2a and S3a). No difference was detected in growth plate size (Fig. S3a), area proportions of different regions (Fig. S3b), and height of HZ (Fig. S3c) at P0 between Het and SMA group, suggesting that the skeletal developmental defects in SMA mice occurred postnatally. The size difference of the growth plates between SMA and HET mice was more pronounced at P7 compared to P4 (Fig. 2a). Starting from P4, the percentage of HZ area of SMA mice became higher than Het mice (Fig. 2b). Moreover, HZ height in SMA mice was slightly higher at P4 with no statistical difference and significantly higher at P7 (Fig. 2c).

We then investigated whether restoring SMN immediately after birth could rescue defects in endochondral ossification and bone development. In line with previous work, we utilized ASO10-29, an MOE-modified antisense oligonucleotide that promotes exon 7 inclusion of SMN2 gene to increase SMN levels.^26^ ASO10-29 was administrated to newborn pups at dose of 90 mg/kg, twice between P0 and P1 (Fig. S4a), and significantly increased SMN protein in SMA femur on P7 (Fig. S4b). Increased SMN rescued bone dysplasia in SMA mice according to the alcian blue and alizarin red double staining result (Fig. S4c). After ASO10-29 administration, both percentage and height of HZ in SMA mice were restored to levels comparable to those in Het mice (Fig. 2b, c). Moreover, the proportion of RZ area, which was reduced in untreated SMA mice at P7, was markedly increased following ASO10-29 treatment (Fig. S4d). The PZ area proportion did not exhibit any significant differences between the Het and SMA groups, regardless of whether it was at P4, P7, or after ASO treatment (Fig. S4e).

Safranin O-Fast Green (SF) staining revealed that cell size of hypertrophic chondrocytes increased and cell density decreased in HZ of Het mice from P4 to P7 (Fig. 2d–f), which is a natural phenomenon during endochondral ossification.^27^ However, the hypertrophic chondrocytes in HZ of SMA mice exhibited smaller size and higher density compared to the Het group, a condition that was recovered by ASO10-29 treatment, indicating an increase of immature hypertrophic chondrocytes. (Fig. 2d–f). At P4 and P7, Von Kossa (VK) staining showed reduced calcification in the metaphysis, while more calcium deposition occurred in the HZ of SMA mice (Fig. 2g–i), indicating the inhibited transition from HZ to ossification zone. ASO10-29 reduced the calcification rate in HZ and increased it in metaphysis (Fig. 2g–i). Taken together, SMA mice exhibited an abnormal growth plate anatomy characterized by suppressed differentiation of hypertrophic chondrocytes. The bone dysplasia could be rescued by SMN supplementation, which consequently alleviated the restriction on long bone development.

### SMN deficiency dysregulates expression of genes related to survival and differentiation of chondrocytes

**Fig. 3:**
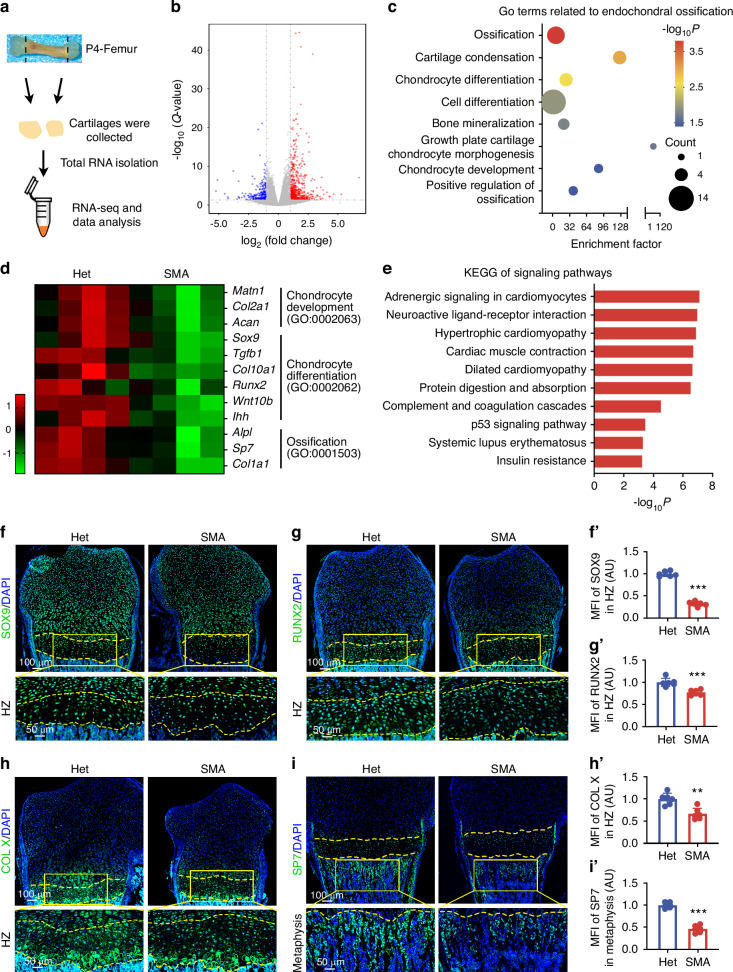
SMN deficiency suppresses endochondral ossification progression. **a** Schematics of the procedure for isolating P4 growth plate cartilages for the RNA-seq analysis. The femoral length of Het mice at P4 was ~5 mm. **b** Volcano plot showing DEGs between cartilages from SMA and Het mice at P4. **c** Endochondral ossification related GO enrichment terms of DEGs between cartilages from SMA and Het mice at P4. **d** Heatmap representing differentially expressed genes associated with the GO-enriched terms “Chondrocyte development”, “Chondrocyte differentiation”, and “Ossification” between cartilages from SMA and Het mice at P4. **e** Top 10 KEGG pathway analysis of DEGs between cartilages from SMA and Het mice at P4. Immunofluorescence staining of SOX9 (**f**), RUNX2 (**g**), COL X (**h**), and SP7 (**i**) in growth plates from SMA and Het mice at P4. Yellow dashed lines indicate the boundary of the HZ. Histograms show MFI of SOX9 (**f**’), RUNX2 (**g**′), and COL X (**h**′) in HZ, and SP7 in metaphysis (**i**′) (*n* = 6 per group). Results were relative to the Het group. *P*-value was derived from two-tailed unpaired Student’s *t* test. Data are presented as the mean ± SD. ^*^P < 0.05, ^**^P < 0.01, ^***^P < 0.001

To understand the molecular mechanism of endochondral ossification defects in SMA mouse model, we conducted bulk RNA sequencing on the growth plate cartilages of P4 SMA and Het mice (Fig. 3a). Samples from SMA mice were distinguishable from those of control mice according to principal component analysis (Fig. S5a). Genes that were upregulated or downregulated >2 folds with a *P*-value < 0.05 were considered significantly differentially expressed. We identified 633 upregulated genes and 241 downregulated genes (Fig. 3b). Analysis of gene ontology (GO) enrichment for biological processes showed that ossification, chondrocyte differentiation, and chondrocyte development were predominantly altered in SMA growth plates (Fig. 3c). Genes related to chondrocyte development, including matrilin 1 (*Matn1*), collagen Ⅱ type Ⅰ (*Col2a1*), and aggrecan (*Acan*) were downregulated (Fig. 3d). Chondrocyte differentiation related genes, including SRY-box transcription factor 9 (*Sox9*), transforming growth factor beta 1 (*Tgfb1*), *Col10a1*, RUNX family transcription factor 2 (*Runx2*), Wnt family member 10B (*Wnt10b*), and indian hedgehog signaling molecule (*Ihh*) were reduced in SMA group (Fig. 3d). Ossification related genes, including alkaline phosphatase (*Alpl*), Sp7 transcription factor (*Sp7*), and collagen Ⅰ type Ⅰ (*Col1a1*) also significantly decreased in SMA growth plate cartilages (Fig. 3d).

Additionally, Kyoto encyclopedia of genes and genomes (KEGG) pathway analysis of significantly differentially expressed genes revealed that several of the top enriched pathways involve sarcomeric and cytoskeletal genes commonly associated with cardiac muscle function, including *Myl2*, *Myl3*, *Myh6*, and *Myh7* (Fig. 3e). Given that proper cytoskeletal remodeling is essential for chondrocyte hypertrophy and growth plate morphogenesis, the enrichment of these genes suggests that hypertrophic chondrocytes in SMA mice may experience aberrant cytoskeletal dynamics, potentially disrupting their terminal differentiation during endochondral ossification. On the other hand, insulin administration has been shown to promote chondrocyte differentiation and maturation.^28^ Both insulin deficiency and insulin resistance are known to exert deleterious effects on bone tissue.^29^ Therefore, the enrichment of insulin resistance pathways suggests that SMN deficiency may impair chondrocyte differentiation through disrupted insulin signaling.

Moreover, enrichment of the p53 signaling pathway (Fig. 3e) suggests dysregulated cell proliferation and apoptosis.^30^ qPCR validation showed downregulation of proliferation marker *Mki67*, and upregulation of *Cdkn1a* and the pro-apoptotic gene *Bax* in SMA growth plate cartilage (Fig. S5b), indicating enhanced cell cycle arrest and apoptosis. However, TUNEL staining revealed positive signals only in the periosteum, but not in chondrocytes within the growth plate (Fig. S5c). This suggests that although *Bax* expression is elevated, chondrocytes have not yet undergone apoptosis at this stage. Therefore, apoptosis is unlikely to be the primary mechanism underlying impaired endochondral ossification caused by SMN deficiency.

In conclusion, SMN deficiency impairs expression of genes related to proliferation and differentiation in chondrocytes.

### SMN deficiency suppresses endochondral ossification progression

Apart from cell proliferation and apoptosis, chondrocyte differentiation is crucial in the process of endochondral ossification and bone development. Chondrocyte differentiation is initially controlled by SOX9 and then shifts to RUNX2 during hypertrophy. Then COL X-marked hypertrophic chondrocytes regulate matrix mineralization and subsequently differentiate into SP7-dependent osteoblasts to promote bone ossification.^31,32^ We examined expression of SOX9, RUNX2, COL Ⅹ, and SP7 in SMA growth plates through immunofluorescence. SOX9 showed a significant reduction throughout the growth plates of P4 SMA mice, especially in HZ (Fig. 3f, Fig. S5d, e). RUNX2, primarily expressed in hypertrophic chondrocytes, was also downregulated across P4 SMA growth plates (Fig. 3g, Fig. S5f, g). Consistent with increased HZ height, the range of COL X-positive cells expanded longitudinally in P4 SMA mice growth plate cartilages, whereas the mean fluorescence intensity (MFI) of COL X was markedly lower compared to the Het mice, reflecting restricted mature of hypertrophic chondrocytes (Fig. 3h). Moreover, the level of SP7 decreased in P4 SMA metaphysis, indicating restricted bone ossification (Fig. 3i).

To confirm that the restoration of SMN can reverse these changes, ASO10-29 was utilized to elevate SMN levels. Immunofluorescence staining of growth plate sections at P7 revealed a widespread decrease in SOX9 and RUNX2 levels throughout the growth plates of SMA mice at P7 (Fig. S6a, b), consistent with the trends seen at P4. MFI of COL X and SP7 was downregulated in HZ and metaphysis of SMA mice, respectively (Fig. S6c, d). In growth plates of SMA mice treated with ASO10-29, we observed marked upregulation of SOX9, RUNX2, COL X, and SP7 levels (Fig. S6a–d).

Overall, these results suggest that SMN loss suppresses the differentiation of mature hypertrophic chondrocytes, thereby inhibiting endochondral ossification. Notably, these defects can be reversed by early restoration of SMN protein levels.

### SMN deficiency dysregulates pre-mRNA alternative splicing in genes linked to endochondral ossification

**Fig. 4:**
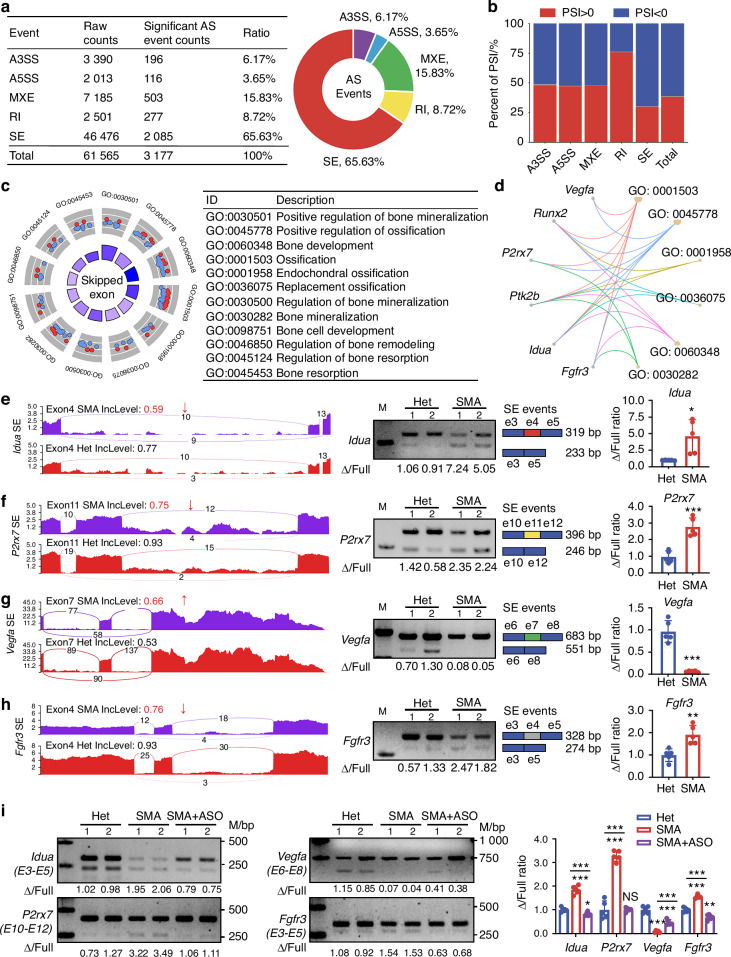
SMN deficiency dysregulates pre-mRNA alternative splicing in genes linked to endochondral ossification. **a** Alternative splicing profile of SMA cartilages compared to control littermates. **b** Percentage of splicing events per category with an increased or decreased inclusion. **c** Endochondral ossification related biological process in GO enrichment of genes with significant SE events. **d** Cnetplot revealing the top 6 genes in these enriched GO terms. Sashimi plots of the different SE events for the genes *Idua* (**e**), *P2rx7* (**f**), *Vegfa* (**g**), and *Fgfr3* (**h**). Semi-quantitative real-time PCR analysis of these genes in cartilages from SMA and Het mice at P4 was presented. Exon skipping ratios were relative to the Het group (*n* = 5 per group). *P*-value was derived from Wilcoxon rank-sum test. **i** Semi-quantitative real-time PCR analysis of *Idua*, *P2rx7*, *Vegfa*, and *Fgfr3* in cartilages from Het, SMA, and SMA mice treated with ASO10-29 at P7. Exon skipping ratio was relative to the Het group (*n* = 5 per group). *P*-value was derived from Wilcoxon rank-sum test. Data are presented as the mean ± SD. ^*^P < 0.05, ^**^P < 0.01, ^***^P < 0.001

SMN protein plays a crucial role in biogenesis of small nuclear ribonucleoproteins, which are essential for RNA splicing events.^33^ We investigated the alternative splicing profile of SMA growth plate cartilages compared to control littermates at P4. Five main types of alternative splicing, including alternative 3′ splice site (A3SS), alternative 5′ splice site (A5SS), skipped exon (SE), mutually exclusive exons (MXE), and retained intron (RI), were analyzed. Using a significant threshold of *P* < 0.05 and ΔPSI > 0.1, we identified a total of 3177 significant splicing events within 2159 genes. Pre-mRNA splicing in SMA cartilages showed changes in A3SS (6.17%), A5SS (3.65%), MXE (15.83%), RI (8.72%), and SE (65.63%) (Fig. 4a). We observed a higher number of significant AS events with decreased inclusion in SE (69.9%) and increased inclusion in RI (76.2%) compared to Het group (Fig. 4b). Since SMN deficiency had the largest effect on SE, we further analyzed genes with significant SE events using GO analysis and found significant enrichment in endochondral ossification, bone development, bone mineralization, bone resorption, and bone remodeling (Fig. 4c). Six transcripts, including vascular endothelial growth factor A (*Vegfa*), *Runx2*, purinergic receptor P2X, ligand-gated ion channel, 7 (*P2rx7*), PTK2 protein tyrosine kinase 2 beta (*Ptk2b*), alpha-L-iduronidase (*Idua*), and fibroblast growth factor receptor 3 (*Fgfr3*), were widely involved in the affected biological pathways (Fig. 4d). Using rMATS Sashimi plot software, we visualized the SE events of these six genes. Semiquantitative PCR validated increased SE events in exon 4 of *Idua*, exon 11 of *P2rx7*, and exon 4 of *Fgfr3*, and decreased SE event in exon 7 of *Vegfa* in cartilages of P4 SMA mice (Fig. 4e–h), while no difference in SE events was detected in *Runx2* and *Ptk2b* (Fig. S7a, b). In growth plates of P7 SMA mice following ASO10-29 injection, aberrant splicing events were corrected, resulting in increased exon inclusion levels for *Idua*, *P2rx7*, *Fgfr3*, as well as restored exon skipping for *Vegfa* (Fig. 4i). Analysis of *Smn1* alternative splicing revealed an 85.5% reduction in exon 7 inclusion in the SMA group (Fig. S7c). This finding aligns with the established mechanism of SMA mouse model, in which exon 7 of *Smn1* was mutated to induce SMN knockdown. Moreover, we examined the expression of these genes through qPCR. Gene levels of *Idua*, *P2rx7*, *Fgfr3*, and *Runx2* significantly decreased in SMA cartilages, while *Vegfa* and *Ptk2b* showed no difference compared to Het group (Fig. S7d). Overall, SMN deficiency broadly dysregulated pre-mRNA alternative splicing patterns of genes linked to endochondral ossification in SMA growth plate cartilages.

### SMN deficiency leads to reduction of YBX1

**Fig. 5:**
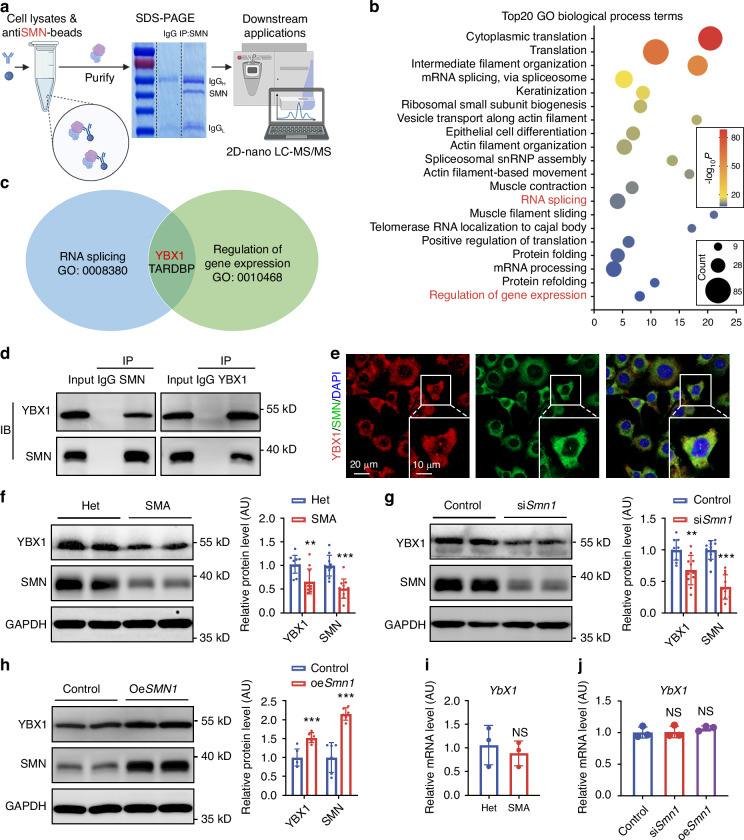
SMN deficiency leads to reduction of YBX1. **a** Schematics of the procedure for mass spectrometric analysis. **b** Top 20 GO biological process terms of SMN-binding proteins. **c** Venn diagrams showing the overlapping SMN-binding proteins between RNA splicing (GO: 0008380) and regulation of gene expression (GO: 0010468). **d** Co-IP analyses of the interaction between SMN and YBX1 in ATDC5 cells. **e** Immunofluorescence staining of YBX1 (red) and SMN (green) in ATDC5 cells. Nuclei were stained with DAPI. **f**–**h** Western blot analysis and corresponding quantifications showing relative protein levels of YBX1 and SMN in cartilage tissues from P4 SMA and Het mice (f, *n* = 11), ATDC5 cells transfected with si*Smn1* (g, *n* = 11) and ATDC5 cells transfected with oe*Smn1* (h, *n* = 6). GAPDH was used as the loading control and results were relative to the Het or control group. **i**, **j** Relative mRNA level of *Ybx1* in cartilage tissues from P4 SMA and Het mice (*n* = 3) (**i**); ATDC5 cells transfected with si*Smn1* and oe*Smn1* (*n* = 3) (**j**). *Gapdh* was used as the loading control and results were relative to the Het or control group. *P*-value was derived from Wilcoxon rank-sum test. Data are presented as the mean ± SD. ^*^P < 0.05, ^**^P < 0.01, ^***^P < 0.001

To identify potential key regulators that modulate gene transcription and alternative splicing in SMA growth plate cartilages, we performed mass spectrometry-based quantitative proteomics which revealed 827 proteins as SMN-binding targets (Fig. 5a). GO analysis indicated that SMN-binding proteins were closely related to RNA splicing and regulation of gene expression (Fig. 5b). Venn analysis showed that YBX1 and TAR DNA binding protein (TARDBP) were involved in both biological processes (Fig. 5c). Importantly, YBX1 knockout has been proven to cause osteopenia,^34^ while its function in SMA-related endochondral ossification defects remains unclear. Interaction between SMN and YBX1 was verified via Co-IP (Fig. 5d). According to immunofluorescence staining, co-localization of SMN and YBX1 existed in cytoplasm, but not in nucleus (Fig. 5e). Furthermore, SMN deficiency reduced YBX1 protein levels in both SMA growth plate cartilage and ATDC5 cells, whereas SMN overexpression significantly restored YBX1 protein levels (Fig. 5f–h). However, *Ybx1* mRNA levels remained unchanged under both knockdown and overexpression conditions (Fig. 5i, j), indicating a post-transcriptional regulatory mechanism. In vivo, ASO10-29 treatment significantly restored YBX1 levels throughout the growth plates of SMA mice (Fig. S8a). These findings suggest that SMN positively regulates YBX1 protein expression at the post-transcriptional level, potentially influencing RNA splicing and gene expression.

### YBX1 knockdown induces SMA-like phenotypes in chondrocytes

**Fig. 6:**
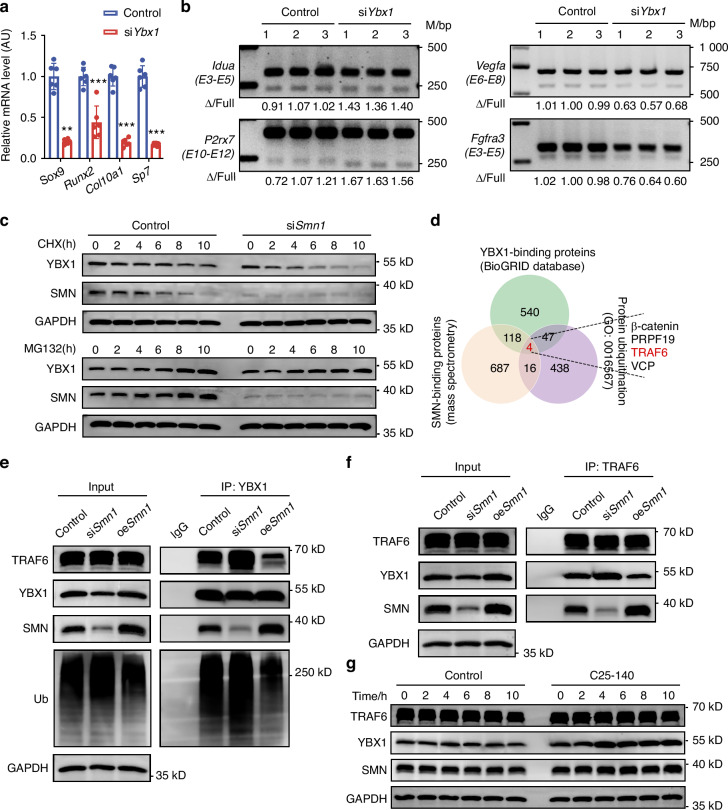
SMN deficiency promotes TRAF6-induced ubiquitination degradation of YBX1. **a** Relative mRNA levels of *Sox9*, *Runx2*, *Col10a1*, and *Sp7* in ATDC5 cells transfected with siCtrl or si*Ybx1* (*n* = 6 per group). *Gapdh* was used as the loading control and results were relative to the control group. *P*-value was derived from two-tailed unpaired Student’s *t* test. **b** Semi-quantitative real-time PCR analysis of *Idua*, *P2rx7*, *Vegfa*, and *Fgfr3* in ATDC5 cells transfected with siCtrl or si*Ybx1*. **c** Western blotting analyses of the effect of si*Smn1* on YBX1 stability in ATDC5 cells incubated with CHX or MG132 at indicated time points. GAPDH was used as the loading control. **d** Venn diagrams of overlapping proteins between SMN binding proteins (data from mass spectrometric analysis), YBX1 (data from BioGRID database), and protein ubiquitination (GO: 0016567). **e** Co-IP analyses of YBX1 interactions with TRAF6, SMN, and ubiquitin in ATDC5 cells transfected with siCtrl, *siSmn1* or oe*Smn1*. GAPDH was used as the loading control. **f** Co-IP analyses of TRAF6 interactions with YBX1 and SMN in ATDC5 cells transfected with siCtrl, *siSmn1* or oe*Smn1*. GAPDH was used as the loading control. **g** Western blotting analyses of the effect of C25-140 on YBX1 stability in ATDC5 cells at the indicated time points. GAPDH was used as the loading control. Data are presented as the mean ± SD. ^*^P < 0.05, ^**^P < 0.01, ^***^P < 0.001

Then we further explored the role of YBX1 in chondrocyte differentiation in SMA model. Knockdown efficiency of si*Ybx1* was confirmed through qPCR (Fig. S9a). *Ybx1* knockdown declined hypertrophic chondrocyte differentiation related genes, including *Sox9*, *Runx2*, *Col10a1*, and *Sp7* in ATDC5 cells (Fig. 6a). In addition, *Ybx1* knockdown also reduced expression of *Idua*, *P2rx7*, *Fgfr3*, and *Runx2* (Fig. S9b), consistent with the gene alterations detected in SMA cartilages (Fig. S7d). Semiquantitative PCR validated increased SE of *Idua* and *P2rx7*, as well as decreased SE of *Vegfa*, after transfection with si*Ybx1* in ATDC5 cells (Figs. 6b and Fig. S9d). Compared with in vivo data from SMA cartilages, SE of *Fgfr3* showed an inverse tendency and was suppressed in ATDC5 cells with *Ybx1* depletion (Figs. 4h, i, 6b, and Fig. S9d). No differences in SE events were detected in *Runx2* and *Ptk2b* (Fig. S9c, d). In summary, decreased YBX1 protein induced by SMN deficiency is likely the primary contributor to aberrant gene expression and splicing patterns in SMA growth plate cartilages.

### SMN deficiency promotes TRAF6-induced ubiquitination degradation of YBX1

Since YBX1 decreased in SMA growth plates without changes in transcription, we adopted cycloheximide (CHX) chasing assay and found that SMN deficiency accelerated the degradation of YBX1, which was prevented by proteasome inhibitor MG132 (Fig. 6c). These results suggested that YBX1 is subject to proteasome-dependent degradation in the absence of SMN. To explore the underlying mechanism, we aimed to identify the specific ubiquitin ligase responsible for YBX1 ubiquitination. Venn analysis between mass spectrometry results of SMN-interacting proteins and BioGRID dataset of YBX1^35^ identified β-catenin, pre-mRNA-processing factor 19 (PRPF19), TRAF6, and valosin containing protein (VCP) as potential candidates (Fig. 6d). TRAF6 has been reported to induce ubiquitination of inhibitor of kappa B kinase (IKK) that binds to SMN.^36^ Further experiments confirmed the interaction between SMN, YBX1 and TRAF6 (Fig. S10a). We then investigated the effects of SMN deficiency on TRAF6 level and detected no difference in SMA growth plate cartilages (Fig. S10b) or ATDC5 cells transfected with si*Smn1* (Fig. S10c), compared to their respective control groups. These findings suggest that SMN knockdown does not directly alter TRAF6 protein levels but rather enhances TRAF6-mediated ubiquitination and subsequent degradation of YBX1. Co-IP analysis confirmed that SMN deficiency increased the interaction between TRAF6 and YBX1, leading to elevated ubiquitin tagging of YBX1 (Fig. 6e, f). Conversely, SMN overexpression reduced this interaction and ubiquitination, thereby restoring YBX1 protein levels (Fig. 6e, f). Moreover, treatment with C25-140, a selective inhibitor of TRAF6’s E3 ubiquitin ligase activity,^37^ effectively elevated YBX1 levels, thereby underscoring the critical role of TRAF6 in mediating YBX1 ubiquitin-dependent proteasomal turnover (Fig. 6g). Together, SMN deficiency promotes YBX1 degradation through TRAF6-induced ubiquitination.

### Loss of SMN in chondrocyte caused retardation of bone development

**Fig. 7:**
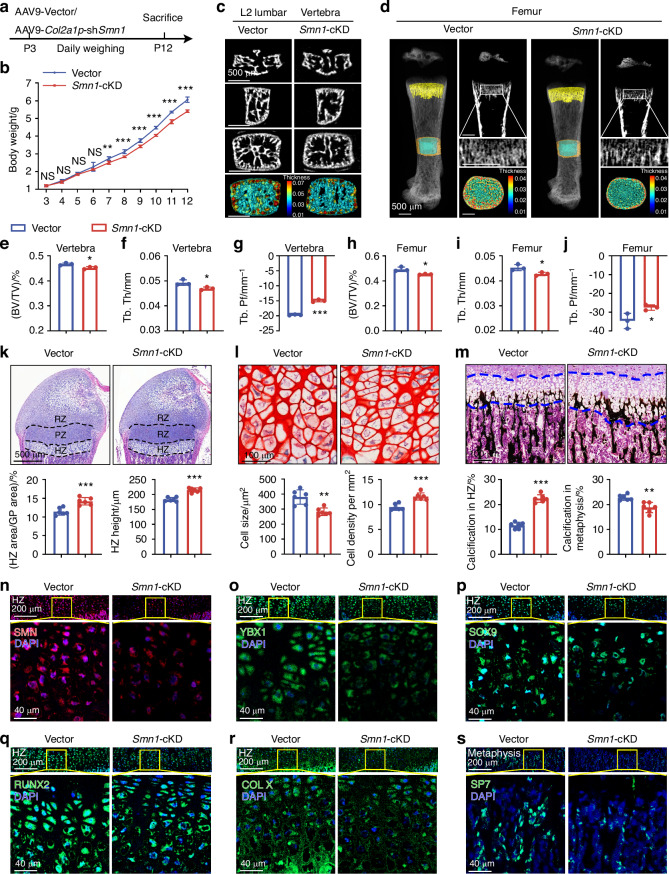
Loss of SMN in chondrocytes caused retardation of bone development. **a** Experimental design involves the retro-orbital injection of AAV9-Vector/AAV9-*Col2a1*p-sh*Smn1* (2 ×10^11^ vg/g) into WT mice at P3, with daily weight monitoring until P12. **b** Weight measurements of control and *Smn1*-cKD mice from P3 to P12 (*n* = 6 per group). *P*-value was derived from two-tailed unpaired Student’s *t* test. Representative micro-CT images of L2 vertebra (**c**) and femur (**d**) from control and *Smn-* cKD mice at P12. The pseudocolor represents the thickness of bone trabecula. Quantitative analysis of trabecular bone microarchitecture (BV/TV, Tb.Th, and Tb.Pf) in vertebra (**e**–**g**) and femur (**h**–**j**) from control and *Smn1*-cKD mice at P12 (*n* = 3 per group). *P*-value was derived from Wilcoxon rank-sum test. **k** HE staining of growth plates from control and *Smn1*-cKD mice at P12. Histograms show ratio of HZ area and HZ height in growth plates (*n* = 6 per group). *P*-value was derived from two-tailed unpaired Student’s *t* test. **l** SF staining of hypertrophic chondrocytes from control and *Smn1*-cKD mice at P12. Histograms show cell size and cell density of hypertrophic chondrocytes in HZ (*n* = 6 per group). *P*-value was derived from two-tailed unpaired Student’s *t* test. **m** VK staining of growth plates and metaphysis from control and *Smn1*-cKD mice at P12. Histograms show ratio of calcification in HZ and metaphysis (*n* = 6 per group). *P*-value was derived from two-tailed unpaired Student’s *t* test. Immunofluorescence staining of SMN (**n**), YBX1 (**o**), SOX9 (**p**), RUNX2 (**q**), COL X (**r**), and SP7 (**s**) in hypertrophic zone or metaphysis of control and *Smn1*-cKD mice at P12. The regions outlined with yellow boxes in the upper panels are shown at higher magnification in the corresponding lower panels. Full-length views of the growth plates and their associated MFI data are provided in Fig. S12a–f. Data are presented as the mean ± SD. ^*^P < 0.05, ^**^P < 0.01, ^***^P < 0.001

To preclude the possibility of indirect impacts of SMN on abnormal bone development, we conducted conditional knockdown of chondrocytic *Smn1* (*Smn1*-cKD mice) by injection of AAV9-*Col2a1*p-sh*Smn1* into WT mice at P3 and daily record their weight until P12 (Fig. 7a). Immunofluorescence verified the knockdown efficiency of SMN in entire growth plate of *Smn1*-cKD mice (Fig. 7n and Fig. S12a). A significant difference of weight between control and *Smn1*-cKD group was detected starting from P7 (Fig. 7b). However, femur length was not reduced in *Smn1*-cKD mice, unlike in mice with systemic *Smn1* knockdown (Fig. S11a). Micro-computed tomography (Micro-CT) analysis of L2 vertebra showed reduced bone volume fraction (BV/TV) and attenuated trabecular thickness (Tb.Th) in *Smn1*-cKD mice (Fig. 7c, e, f). Moreover, the increased trabecular bone pattern factor (Tb.Pf) in *Smn1*-cKD group indicated a transition in trabecular morphology from plate-like to rod-like structures, suggesting a phenotype of osteoporosis (Fig. 7g). However, the bone mineral density (BMD), trabecular separation, and trabecular number (Tb.N) showed no difference after *Smn1*-cKD (Fig. S11b–d). Similar to the vertebrae, the femurs of *Smn1*-cKD mice also exhibited lower BV/TV and Tb.Th (Fig. 7d, h, i), increased Tb.Pf (Fig. 7j), while maintaining similar BMD, Tb.Sp, and Tb.N (Fig. S11e–g) compared to the control group. The cortical bone in the femurs of *Smn1*-cKD mice also exhibited lower trabecular thickness (Ct.Th) and consistent BMD and bone area fraction (BA/TA) compared to the control group (Fig. S11h–j).

To determine whether conditional knockdown of *Smn1* in chondrocytes causes bone development defects, we conducted histochemical and immunofluorescence staining to compare the anatomical structure of the growth plate and the levels of endochondral ossification-related proteins. The proportion of HZ area and HZ height were both elevated in *Smn1*-cKD mice, while proportion of RZ and PZ area were similar to that in control mice (Fig. 7k, Fig. S11k, l). SF staining demonstrated smaller size and higher density in hypertrophic chondrocytes of *Smn1*-cKD mice (Fig. 7l). VK staining revealed more calcification in HZ and delayed calcification in metaphysis of WT mice after *Smn1*-cKD (Fig. 7m). Moreover, the protein levels of YBX1, SOX9, RUNX2 exhibited a widespread decrease in growth plates of *Smn1*-cKD mice, particularly in HZ (Fig. 7o–q and Fig. S12b–d). The MFI of COL X was significantly lower in HZ, and SP7 levels decreased in metaphysis of *Smn1*-cKD mice compared to the control mice (Fig. 7r, s, Fig. S12e, f), indicating a retardation in the progression of bone ossification.

In conclusion, the bone development abnormalities in SMA mice are primarily caused by the absence of *Smn1* in chondrocytes, independent of SMN deficiency in other tissues.

### Chondrocyte-specific SMN restoration partially reverses endochondral ossification defects in SMA mice

**Fig. 8:**
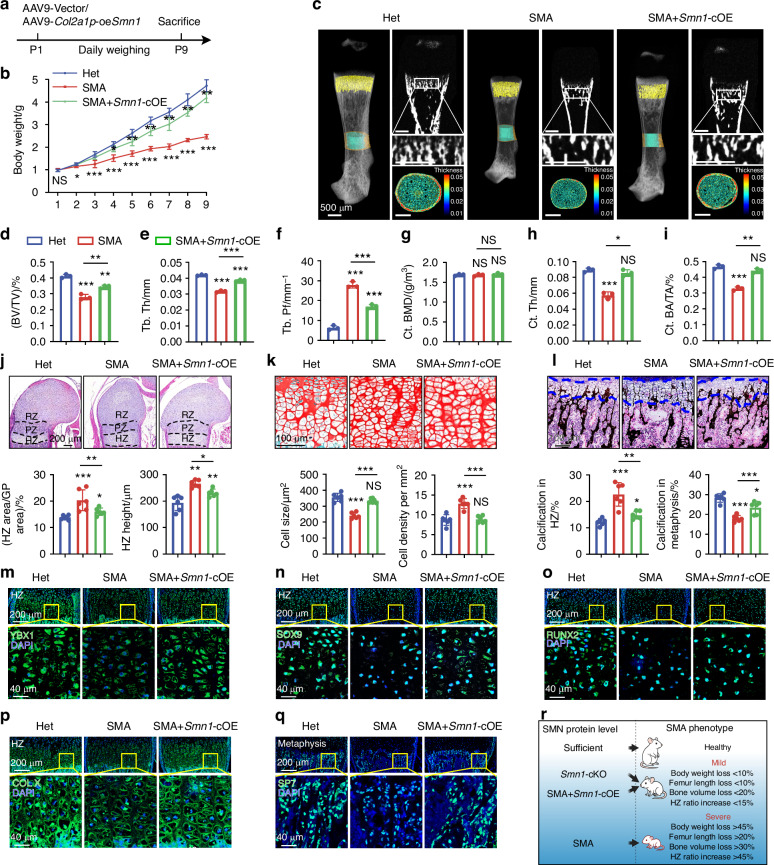
Chondrocyte-specific SMN restoration partially reverses endochondral ossification defects in SMA mice. **a** Experimental design involves the retro-orbital injection of AAV9-Vector/ AAV9-*Col10a1p*-oe*Smn1* (2 ×10^11^ vg/g) into WT mice at P1, with daily weight monitoring until P9. **b** Weight measurements of Het, SMA and *Smn1*-cOE mice from P1 to P9 (*n* = 6 per group). *P*-value was derived from two-tailed unpaired Student’s *t* test. **c** Representative micro-CT images of femurs from Het, SMA and *Smn1*-cOE mice at P9. The pseudocolor represents the thickness of bone trabecula. **d**–**i** Quantitative analysis of trabecular bone microarchitecture (BV/TV, Tb.Th, and Tb.Pf) and cortical bone microarchitecture (Ct.BMD, Ct.Th, and Ct.BA/TA) in femurs from Het, SMA and *Smn1*-cOE mice at P9 (*n* = 3 per group). *P*-value was derived from Wilcoxon rank-sum test. **j** HE staining of growth plates from Het, SMA and *Smn1*-cOE mice at P9. Histograms show ratio of HZ area and HZ height in growth plates (*n* = 6 per group). *P*-value was derived from two-tailed unpaired Student’s *t* test. **k** SF staining of hypertrophic chondrocytes from Het, SMA and *Smn1*-cOE mice at P9. Histograms show cell size and cell density of hypertrophic chondrocytes in HZ (*n* = 6 per group). *P*-value was derived from two-tailed unpaired Student’s *t* test. **l** VK staining of growth plates and metaphysis from Het, SMA and *Smn1*-cOE mice at P9. Histograms show ratio of calcification in HZ and metaphysis (*n* = 6 per group). *P*-value was derived from two-tailed unpaired Student’s *t* test. Immunofluorescence staining of YBX1 (**m**), SOX9 (**n**), RUNX2 (**o**), COL X (**p**), and SP7 (**q**) in hypertrophic zone or metaphysis from Het, SMA and *Smn1*-cOE mice at P9. The regions outlined with yellow boxes in the upper panels are shown at higher magnification in the corresponding lower panels. Full-length views of the growth plates and their associated MFI data are provided in Fig. S14a–e. **r** Schematic illustration showing the relationship between SMN protein level and SMA phenotypic outcomes. Data are presented as the mean ± SD. ^*^P < 0.05, ^**^P < 0.01, ^***^P < 0.001

Although *Smn1*-cKD mice exhibited alterations in multiple aspects of endochondral ossification, these changes were less pronounced than those observed between SMA and HET mice. This suggests a compensatory support that mitigates the effects of SMN deficiency in chondrocytes. We conditionally overexpressed *Smn1* in chondrocytes (*Smn1*-cOE) within the SMA mouse model to evaluate whether restoring SMN in cartilage alone could rescue defects in endochondral ossification under systemic SMN deficiency. We administered AAV9-*Col10a1p*-oe*Smn1* to SMA mice at P1 and performed analyses at P9 (Fig. 8a). To evaluate the effect of chondrocyte-specific *Smn1* overexpression, we firstly assessed SMN protein levels in the growth plate by immunofluorescence. *Smn1*-cOE significantly restored SMN expression in the resting and proliferative zones, and even led to supraphysiological SMN levels in the hypertrophic zone (Fig. S13a), confirming the efficiency of AAV9-*Col10a1p*-oe*Smn1* delivery.

Compared to SMA mice, *Smn1*-cOE mice showed substantial recovery of body weight (Fig. 8b) and femur length (Fig. S13b), although these parameters remained lower than those in Het control. Micro-CT analysis demonstrated that the reductions in BV/TV, Tb.Th, and the increase in Tb.Pf observed in SMA mice were significantly ameliorated in *Smn1*-cOE mice (Fig. 8c–f). However, these values in *Smn1*-cOE mice still differed from those in Het mice (Fig. 8c–f), indicating an incomplete rescue of trabecular bone structure. Consistent with findings in *Smn1*-cKD mice, neither SMN deficiency nor its restoration affected BMD, Tb.Sp, or Tb.N, implying that SMN primarily influences the morphology rather than the quantity or spatial distribution of trabecular bone (Fig. S13c–e). Interestingly, cortical bone indices such as Ct.Th and Ct.BA/TA were fully restored to Het levels in *Smn1*-cOE mice (Fig. 8g–i), suggesting that SMN deficiency in non-chondrocytic cell types may primarily contribute to trabecular, but not cortical bone alterations.

Histological and immunofluorescence analyses further supported these findings. The enlarged HZ area, decreased RZ area, increased HZ height, and altered mineralization patterns in SMA growth plates were significantly rescued in *Smn1*-cOE mice (Fig. 8j, l, Fig. S13f, g). Notably, the size and density of hypertrophic chondrocytes were completely restored (Fig. 8k). Protein levels of YBX1, SOX9, RUNX2, and COL X in the hypertrophic zone, along with SP7 expression in the metaphysis, were all upregulated compared to SMA mice (Figs. 8m–q and Fig. S14a–e), supporting the role of SMN in regulating the terminal differentiation and function of hypertrophic chondrocytes.

These findings, taken together with the phenotypic alterations observed in *Smn1*-cKD mice, demonstrate that chondrocytic SMN expression is essential for the progression of hypertrophic chondrocyte-driven endochondral ossification. Meanwhile, SMN in non-chondrocytic cells appears to partially compensate for the loss of SMN in chondrocytes, thereby mitigating the severity of ossification defects in *Smn1*-cKD mice (Fig. 8r).

## Discussion

In SMA patients and mouse models, inhibition of bone development has been reported, partially independent of neuromuscular degeneration.^19^ Most mammalian skeletons are composed of bones that originate from cartilage templates through endochondral ossification. However, whether low levels of SMN affect endochondral ossification in a cell-autonomous manner has not been previously addressed. Here, we demonstrate that *Smn1* is involved in hypertrophic chondrocytes differentiation and *Smn1* depletion in early symptomatic stages leads to dwarfism by impeding the differentiation of hypertrophic chondrocytes and turnover from HZ to ossification zone in growth plates. Mechanistically, SMN deficiency enhances the interaction between TRAF6 and YBX1, promoting ubiquitination degradation of splicing factor YBX1. Decreased YBX1 causes mis-splicing in genes crucial for endochondral ossification, including *Fgfr3*, *Vegfa*, *Idua*, and *P2rx7*. These findings underscore the regulatory role of SMN in gene expression and alternative splicing during endochondral ossification, elucidating the upstream molecular events contributing to impaired bone development in SMA.

A recent study demonstrated that mice lacking *Smn1* gene specifically in limb mesenchymal progenitor cells, a type of bone-forming cells, and having one copy of *SMN2* (*SMN2* 1-copy *Smn1*^ΔMPC^), exhibited inhibited bone development and decreased body size from embryonic phase. Interestingly, our severe SMA mice lacking *Smn1* and possessing two copies of *SMN2* gene (*Smn1*^−/−^; *SMN2*^*2TG/0*^) did not exhibit significant abnormalities in limb length and body size until P4, indicating the delayed SMA symptoms. In the absence of *Smn1* gene, the number of *SMN2* gene copies inversely correlates with the age of disease onset and severity. Histologically, *SMN2* 1-copy *Smn1*^ΔMPC^ mutants and SMA mice showed reduced height and chondrocyte number in HZ of growth plate cartilages, along with decreased cell proliferation marked by Ki67.^19,20^ Our findings further indicate suppressed cell proliferation in growth plate cartilages. Previous studies reported reduced cell number and/or decreased proliferation in various tissues such as the heart, hippocampus, retinal, and optic nerve of SMA mouse models, suggesting that alteration in cell proliferation is a widespread phenomenon across multiple tissues in SMA.^38–40^ During endochondral ossification, chondroblasts proliferate rapidly at the distal end, organizing themselves into columns perpendicular to the long axis of growth. Thus, inhibited chondrocytes proliferation is likely a cause of short bone in SMA. However, our study revealed a relatively expanded HZ and increased hypertrophic chondrocyte density in severe SMA mice with two copies of *SMN2* gene, phenomena that cannot be explained solely by suppressed proliferation, suggesting that different SMN protein levels have various impacts on bone development.

Hypertrophic chondrocytes are the principal engine of bone development, serving as the primary drivers of mineralization of their surrounding matrix and attracting blood vessels and chondroclasts. Subsequently, hypertrophic chondrocytes either undergo apoptosis^31^ or transdifferentiation into osteoblasts for trabecular bone formation in the embryonic and neonatal stages in mouse models.^21,41,42^ Therefore, the observed increase in HZ height and hypertrophic chondrocyte density may result from several factors, including decreased apoptosis and vascular invasion, accelerated differentiation of chondrocytes, accumulation of immature hypertrophic chondrocytes and delayed terminal transdifferentiation into osteoblasts.^43^ Our data showed upregulation of apoptosis related gene *Bax* in SMA cartilages, suggesting that enhanced apoptosis does not account for the expansion of HZ. Studies reported vascular-related defects in severe SMA patient and mouse models, including digital necrosis, vascular thrombosis, and decreased capillary density in various tissues.^44–47^ There was no significant difference in *Vegfa* gene expression, but increased *Vegfa121* and decreased *Vegfa165* were observed in SMA growth plate cartilages, which encodes heparin-sulfate binding domains crucial for VEGFA’s interaction with the extracellular matrix.^48^ VEGFA165 can effectively promotes the growth and the proliferation of endothelial cells, whereas Wang et al. demonstrated that VEGFA121 and VEGFA165 exerted opposing effects on angiogenesis due to activation of different phosphorylation sites of VEGFR2.^49^ Thus, impaired vascular invasion due to reduced SE of *Vegfa* likely contributes to the expanded zone of hypertrophic chondrocytes in SMA mice.

Accelerated differentiation of chondrocytes from the proliferative to the early hypertrophic stage and accumulation of immature hypertrophic chondrocytes could also account for the expansion of the zone of hypertrophic chondrocytes. But two critical regulatory factors SOX9 and RUNX2 were downregulated in SMA growth plates, inhibiting the differentiation of chondrocytes and increasing accumulation of immature hypertrophic chondrocytes. This led to a reduced number of mature hypertrophic chondrocytes; however, cell density in HZ of SMA mice is significantly increased and cell size is smaller, as a matter of fact. In RUNX2 deficient mice, hypertrophic chondrocytes maturation was inhibited and were absent in most skeletons.^50^ Moreover, RUNX2 deficiency reduced the number of transdifferentiated osteoblasts from hypertrophic chondrocytes, leading to a reduction in trabecular bone formation.^51^ Lui et al. demonstrated that downregulation of SOX9 in hypertrophic chondrocytes promotes transdifferentiation of hypertrophic chondrocytes into osteoblasts,^52^ which is contradictory with our results. But considering the different role of SOX9 and RUNX2 in regulating transdifferentiation of hypertrophic chondrocytes, we suppose that the decreased RUNX2 level inhibited the process of transdifferentiation. Importantly, *Col10a1* expression was restrained in hypertrophic chondrocytes, correlating with excessive ossification in HZ and decreased ossification in metaphysis, indicative of delayed terminal chondrocyte mineralization. In conclusion, the expanded zone of hypertrophic chondrocytes and reduced ossification in severe SMA mouse models are mainly caused by impaired vascular invasion and delayed transdifferentiation and ossification of hypertrophic chondrocytes. However, the mechanism by which SMN deficiency induces these changes remains obscure.

The primary role of SMN protein is to facilitate snRNP assembly by forming SMN complex with Gemins2-8 and Unrip.^33,53^ snRNPs are major components of the spliceosome which regulate pre-mRNA splicing.^54,55^ Loss of SMN altered the stoichiometry of snRNAs and caused widespread defects in pre-mRNA splicing in numerous transcripts of diverse genes in SMA mouse models.^9,56^ As revealed by RNA and single-cell sequencing analysis, SMN is implicated in differentiation of hypertrophic chondrocytes through alternative splicing, yet the key molecules acting downstream of SMN remain elusive. Here, YBX1 was identified as a key protein in SMA cartilages. YBX1, as an RNA-binding protein, is responsible for pre-mRNA transcription and splicing, mRNA stability, and translation.^57,58^ YBX1 regulates multiple cellular processes, including proliferation, apoptosis, cell differentiation, and cell stress response.^59,60^ Depletion of YBX1 inhibits cancer cell proliferation and causes embryonic development defects.^61,62^ Importantly, YBX1 knockdown leads to exon skipping changes in BMSC osteogenesis related genes such as *Fn1*, *Sp7*, and *Spp1*, ultimately shifting BMSC differentiation.^34^ In cartilages of SMA mice, we observed exon skipping changes in endochondral ossification related genes, including *Fgfr3*, *Vegfa*, *Idua*, and *P2rx7*, consistent with in vitro results of YBX1 depletion in ATDC5 cell line. Together, decreased YBX1 expression due to SMN deficiency may be the primary reason for alterations in chondrocyte proliferation, apoptosis, and endochondral ossification of SMA mice.

However, no difference in transcript level of *Ybx1* was observed in SMA, indicating that decreased YBX1 protein is triggered by protein degradation. Poly-ubiquitination and subsequent degradation of YBX1 have been explored in various cell types.^63,64^ In chondrocytes, CHX and MG132 assays confirmed that YBX1 protein degradation occurs through ubiquitin-proteasome system (UPS). SMN was previously reported to interact with several components of the UPS, including UBA1 and several E3 ligases, mediating cell-wide ubiquitination.^10^ By comparing mass spectrometry results of SMN and the Co-IP database of YBX1-binding proteins (BioGRID), TRAF6 was identified as a potential SMN-related E3 ubiquitin ligase inducing YBX1 ubiquitination. Activation of TRAF6 has been shown to suppress cartilage extracellular matrix degradation and inflammation in osteoarthritic rats, highlighting its importance in cartilage homeostasis.^65^ Notably, SMN was identified as a negative regulator of an ubiquitylation complex that includes TRAF6, bendless, and DIAP2 in Drosophila.^66^ Kim EK et al. reported that SMN functions as a novel inhibitor of TRAF6-mediated IKK ubiquitination in BV2 cells.^36^ Nonetheless, our current findings suggest that SMN depletion enhances the interaction between TRAF6 and YBX1, promoting its proteasome-mediated degradation. Based on these results, we speculate that SMN depletion reinforces the ubiquitylation complex composed of TRAF6, leading to the degradation of various targeted proteins, not limited to YBX1 and IKK.

While our study provided detailed insights into inhibited endochondral ossification in SMA mice, potential limitation should be acknowledged. YBX1 degradation induced by SMN depletion may not be limited to growth plate cartilages but could also occur in other tissues such as the brain and spinal cord. Further studies should investigate the interaction between SMN, YBX1, and TRAF6 in other organs to validate our findings. Nonetheless, our data strongly suggest that SMN ablation activates TRAF6-induced YBX1 degradation, disrupting the expression and splicing of genes related to endochondral ossification, ultimately leading to bone development deficiencies.

## Materials and methods

### Mice and in vivo treatment

All mouse studies were conducted in accordance with the guidelines and protocols approved by the Institutional Animal Care and Use Committee (IACUC) of the Second Affiliated Hospital of Soochow University, Jiangsu, China. The study complied with Animal Research: Reporting in Vivo Experiments (ARRIVE) guidelines to minimize the discomfort and pain of the animals. The initial breeding pairs of human *SMN2* transgenic mice were purchased from Jackson Laboratory (stock number 005058), and the severe SMA model (*Smn1*^−*/*−^, *SMN2*^*2TG/0*^) was generated as described.^67^ Genotypes of mice were identified with One Step Mouse Genotyping Kit (Vazyme, PD101-01). For tissue sample collection, mice were sacrificed by CO_2_ asphyxiation, and then femurs or growth plate cartilage tissues were immediately isolated, snap-frozen in liquid nitrogen for storage at −80 °C. Both male and female mice were utilized in all experiments, as no discernible sex differences were noted in any of the measured endpoints.

### Alcian blue-alizarin red double staining of the skeleton

Mice were sacrificed by CO_2_ asphyxiation and fixed in 4% paraformaldehyde for 24 h. Skin and organs of mice were removed before fixation in 95% ethanol for 48 h and then immersed into lacquer thinner for 1 week and again 95% alcohol for 2 days. Tissues were stained with Alcian blue solution (Sigma-Aldrich, B8438) for 24–48 h and destained with 95%, 90%, 40%, and 15% alcohol for 2–3 h, respectively. The ethanol was then replaced with 2% KOH clearing solution for 2 h and then stained with Alizarin red solution (Sigma-Aldrich, A5533) for 15–30 min, followed by incubation in 2% KOH until the soft tissue disintegrated for 1 week. The mice were stored long-term in glycerol.

### Micro-CT

The vertebras and femurs from WT mice injected with AAV9-shCtrl or AAV9-*Col2a1*p-sh*Smn1* were isolated and cleaned of muscles and skin. Afterwards, the femurs were fixed in 4% paraformaldehyde prior to analysis. Imaging of the tissues was conducted using a NEMO micro-CT system (PINGSENG Healthcare, NMC-200). Scanning parameters were set at 90 kV and 88 mA, with a field of view of 10 mm (voxel size of 20 μm; scanning duration of 14 min). The 3D images were visualized using the Quantum GX II’s 3D Viewer software. The region 0.5 mm below the lowest point of upper edge of femoral diaphysis was used to measure trabecular parameters, while the area 0.5 mm around the midpoint of the femoral long axis was used for cortical parameter measurements. Due to the indistinct boundary between the cortical and trabecular bone in the vertebrae, the entire vertebra was considered trabecular bone for calculations.

### Histology and immunofluorescence staining

Femurs were fixed in 4% paraformaldehyde for 24 h and then decalcified with 10% EDTA. Tissues were embedded in paraffin and 5 μm-thick paraffin sections were cut for HE, SF, and immunofluorescence staining. Fixed non-demineralized femurs were used for Von Kossa staining. For immunofluorescence staining, sections were dewaxed and rehydrated and followed with antigen retrieval by Tris-EDTA (PH 9.0) (Abcam, ab93684) in boiling water for 20 min. Sections were permeabilized with 0.3% (v/v) Triton-X100 (Sangon, 9002-93-1) for 10 min and then blocked with 10% (v/v) goat serum for 30 min. Primary antibodies including SMN (Abcam, ab314895, 1:100), SOX9 (Abcam, ab185966, 1:100), RUNX2 (Abcam, ab192256, 1:100), Collagen Type X (Abcam, ab260040, 1:100), SP7 (Abcam, ab209484, 1:500), YBX1 (Abcam, ab76149, 1:100) were incubated overnight at 4 °C. Sections were incubated with corresponding secondary antibodies conjugated to Alexa Fluor 488 (Invitrogen, A-11008) and Alexa Fluor 568 (Invitrogen, A-11004) for 1 h at room temperature. Nuclei were counter-stained with DAPI (Thermo Fisher Scientific, 62248). The images were captured with a confocal microscope (Zeiss, LSM800). The area of different zones in growth plates, cell size, cell density, and calcification area were analyzed using ImageJ.

### Cell culture

The chondrogenic cell line ATDC5 was cultured in Dulbecco’s modified Eagle’s medium (DMEM, Gibco, 11965092) containing 10% fetal bovine serum (Gibco, 16140071) and 1% Penicillin-Streptomycin-Amphotericin B Solution (Procell, PB180121) at 37 °C, in a humidified atmosphere containing 5% CO_2_. The ATDC5 cell line was purchased from Procell (FuHeng, FH0378) and has been authenticated by STR profiling. The siRNAs to deplete *Smn1* or *Ybx1* and plasmids containing ubiquitin were purchased from GenePharma and details are listed in Supplementary Table S1. According to the manufacturer’s instructions, ATDC5 cells were seeded in 6-well plates at 70% confluency, and 100 nmol of each siRNA or 0.1 ng plasmids was transfected into cells in each well, using Lipofectamine 3000 (Invitrogen, L3000001). Otherwise, ATDC5 cells were treated with 20 mol/L C25-140 (MCE, HY-120934), an inhibitor of TRAF6’s ubiquitin ligase activity, for 0, 2, 4, 6, 8 and 10 h before collection.

### Western blot analysis

The growth plate tissues were pulverized in liquid nitrogen and protein extracts were separated on a 10% SDS-polyacrylamide gel and electro-blotted onto PVDF membranes (Bio-Rad, 1620184). The membrane was blocked with 5% non-fat milk for 1–2 h and was incubated overnight at 4 °C with primary antibodies including SMN (Abcam, ab314895, 1:1 000), YBX1 (Abcam, ab76149, 1:5 000), TRAF6 (CST, 67591S), Ubiquitin (CST, 20326S), and GAPDH (Proteintech, 60004-1-Ig). Membranes were incubated with secondary antibodies (Proteintech, SA00001-2, 1:1 000) at room temperature for 1 h. After three washes with TBST, immunolabeling was activated by an Omni-ECL™ Femto Light Chemiluminescence Kit (Epizyme, SQ201) and captured by a GeneGnome XRQ analyzer (Syngen). Gray value was analyzed using ImageJ normalized to GAPDH.

### Co-immunoprecipitation (Co-IP)

Co-IP was carried out using Protein A/G PLUS-Agarose (Santa Cruz, sc-2003) according to the manufacturer’s instructions. Briefly, total proteins were extracted and quantified. A total of 2 mg protein in 500 μL supernatant was incubated with 10 μg anti-SMN (Abcam, ab314895), anti-YBX1 (Abcam, ab76149), anti-TRAF6 (CST, 67591S) or anti-IgG (Abcam, ab6708) antibodies for 12 h at 4 °C. Agarose beads were washed, eluted in sample buffer, and boiled for 10 min at 100 °C. Immune complexes were subjected to Western blot and mass spectrometry analysis. Anti-IgG was used as a negative control.

### Mass spectrometric analysis

In-gel digestion was performed using trypsin as previously described.^68^ After digestion, peptides were extracted from the gel piece with 50% acetonitrile/0.1% formic acid. The extracted peptides were dried in SpeedVacuum concentrator and resuspended in 0.1% formic acid for LC-MS/MS analysis. Peptide samples were separated by EASY nLC-1200 (Thermo Fisher Scientific). The isolated peptides were subjected to Nano source followed by Q Exactive HF-X mass spectrometer. Mass spectra were processed and searched using Proteome Discoverer (version 2.4, Thermo Fisher Scientific) against the Swissprot protein database (release 2022_01). The mass tolerance allowed for the precursor ions is 10 x 10^-6^, while the mass tolerance of fragment ions is set to 0.01 Da. Carbamidomethyl on cysteine was specified as fixed modification, while oxidation on methionine and acetyl on protein N-terminal were specified as variable modification. Peptide confidence was set at high.

### RNA isolation, semi-quantitative and quantitative real-time PCR

Trizol (Invitrogen, 15596026) was used to extract total RNA from growth plate cartilages of mice and ATDC5 cells after pulverizing them in liquid nitrogen with mortar and pestle. One microgram of total RNA was reverse transcribed with Hiscript III Reverse Transcriptase (Vazyme, R302-01). Semi-quantitative real-time PCR was performed with 2× Taq Plus Master Mix (Vazyme, P212-01) in PTC-200 (BIO-RAD, CA, USA). Quantitative real-time PCR was performed with ChamQ SYBR qPCR Master Mix (Vazyme, Q311-02/03) in QuantStudio 5 (Thermo Fisher Scientific) according to the manufacturer’s instructions. The primers for all genes are supplied in Supplementary Tables S2 and S3. Gene expression levels were analyzed relative to *Gapdh*.

### RNA sequencing analysis and resource of scRNA-seq data

Neonatal mice at P4 were euthanized, and the femurs were rapidly harvested in cold PBS. Under a stereo microscope, surrounding soft tissues and metaphyseal bone were removed with micro-scissors. The epiphysis was bisected longitudinally to expose the growth plate and cartilage was carefully dissect with fine forceps. Total RNA was isolated from the growth plate cartilages of the femurs on P4. RNA-seq was performed using an Illumina Genome Analyzer, with an average of 44–52 million mapped reads per sample. The raw data were subjected to QC analyses using FastQC v0.11.7 software. The transcripts were normalized to those of the control group and transcripts with low variance (<0.1) across samples were removed. Statistically significant changes in gene expression were calculated using the DESeq2-package. Genes with FC > 2 and *Q*-value < 0.05 were considered as differentially expressed genes and the DAVID Bioinformatics Resources 6.8. rMATS software was used to calculate the inclusion of a given differentially expressed exon as percent spliced-in (PSI) and assess the fraction of a gene’s mRNA.

The scRNA-seq data were downloaded from two deposited dataset (GSE190616 and GSE179148). We excluded cells with fewer than 200 detected genes, more than 7% mitochondrial genes, or those expressed in ≤5 cells. Cells were categorized into different clusters as described in Long et al., 2022. and marker genes for each cluster were identified using the R package of Seurat 5.^69–73^ Monocle analysis was performed (num_dim = 50) using the Monocle3 R package.^74–77^

### CHX chase assay

ATDC5 cells were transfected with negative control or si*Smn1* for 4 h and then treated with MG132 (20 μmol/L) or CHX (50 μg/mL) for 0, 2, 4, 6, 8 or 10 h. Cells were collected at the indicated time points and subjected to Western blot analysis. All experiments were performed at least three times independently.

### Antisense oligonucleotide and AAV9 treatment

MOE-modified ASO10-29 (5′-ATTCACTTTCATAATGCTGG-3′) with phosphorothioate backbone and all 5-methylcytosines were synthesized by Genepharma. The oligonucleotide solution was injected subcutaneously twice at 90 mg/kg between P0 and P1 and tissue samples were collected at P7. AAV9-shCtrl, AAV9-*Clo2a1*p-sh*Smn1* and AAV9-*Col10a1p*-oe*Smn1* were synthesized by Genechem. The rAAV9 virus was diluted in 50 µL of saline at a concentration of 2 × 10^11^ vg/g and administrated via retro-orbital injection at P1 or P3. Blanching of the superficial temporal vein indicates successful injection.

### Statistical analysis

The Shapiro–Wilk test was performed to evaluate the normality of the numerical data. Normally distributed data are presented as the mean ± standard deviation (SD), while non-normally distributed data are presented as the median (25th percentile, 75th percentile). A two-tailed unpaired Student’s *t* test was used for comparisons between 2 groups obeying normal distribution. One-way ANOVA followed by Tukey post hoc test was applied for comparisons among several groups obeying normal distribution. In condition of an abnormal distribution or a small sample size (*n* < 6), Wilcoxon rank-sum test was applied for comparisons between 2 groups and Kruskal–Wallis test was applied for comparisons among several groups. A *P*-value of <0.05 was considered statistically significant. GraphPad Prism 9.0 software was used for all statistical analyses.

## Supplementary information

Supplemental data

Unedited blot and gel images
