# Supplementary material for: SMN deficiency inhibits endochondral ossification via promoting TRAF6-induced ubiquitination degradation of YBX1 in spinal muscular atrophy
Source: Bone Res. 2025 Dec 1;13:97. doi: 10.1038/s41413-025-00473-6 (PMC12665793; doi:10.1038/s41413-025-00473-6)
Supplement: Supplementary file 1 — Supplemental data [file 41413_2025_473_MOESM1_ESM.pdf]

Supplementary Information

Supplementary Tables

Supplementary Tab. 1 Sequences of siRNAs

**Name**		**Sequence**
Smn1 siRNA	Sense:	5’-CUAUAUGGGUUUCAGACAATT-3’
	Anti-sense:	5’-UUGUCUGAAACCCAUAUAGTT-3’
Ybx1 siRNA	Sense:	5’-GUCAAAUGGUUCAAUGUAATT-3’
	Anti-sense:	5’-UUACAUUGAACCAUUUGACTT-3’
Negative control	Sense:	5’-UUCUCCGAACGUGUCACGUTT-3’
	Anti-sense:	5’-ACGUGACACGUUCGGAGAATT-3’

Supplementary Tab. 2 Sequence of primers for quantitative real-time PCR

**Primer**		**Sequence**
Mki67	F:	5’-GACCGCTCCTTTAGGTATGAAGA-3’
	R:	5’-TCGCCTTGATGGTTCCTTTCC-3’
Cdkn1a	F:	5’-CCGCACAGGAGCAAAGTGT-3’
	R:	5’-ACGAAGTCAAAGTTCCACCG-3’
Bax	F:	5’-TGCAGAGGATGATTGCTGACG-3’
	R:	5’-CAGGGCCTTGAGCACCAG-3’
Sox9	F:	5’-GCGGAGGAAGTCGGTGAAGAATG-3’
	R:	5’-GAAGATGGCGTTAGGAGAGATGTGAG-3’
Runx2	F:	5’-GGACGAGGCAAGAGTTTCACC-3’
	R:	5’-TTAGAGTCATCAAGCTTCTGTCTG-3’
Col10a1	F:	5’-TCTGTGAGCTCCATGATTGC-3’
	R:	5’-GCAGCATTACGACCCAAGATC-3’
Sp7	F:	5’-ACCCAGGAAGAAGCTCACTAT-3’
	R:	5’-TCTTTGTGCCTCCTTTCCCCA-3’

Supplementary Tab. 3 Sequence of primers for semi-quantitative real-time PCR

Primer		Sequence
Idua	F:	5’- GTACGACCTTAGTTGGGACC-3’
	R:	5’- CAGCCCATACCTACCAATGT-3’
P2rx7	F:	5’- GTGGAAAAGCGGACGTTGAT-3’
	R:	5’- GTCCATCTGGGGTCTTGGAA-3’
Vegfa	F:	5’- GGTCAAAAACGAAAGCGCAA-3’
	R:	5’- TCCTCCTCCCAACACAAGTC-3’
Fgfr3	F:	5’- GCTAAATGCCTCCCACGAAG-3’
	R:	5’- CTGGTGCCGGAGCTTGAT-3’
Runx2	F:	5’- GGCACAGACAGAAGCTTGATGAC-3’
	R:	5’- AAAGGGCCCAGTTCTGAAGC-3’
Ptk2b	F:	5’- GACCTCCTCCACAGACCAAC-3’
	R:	5’- CACCATCTGCTTCTGCTGTC-3’

Supplemental Figures

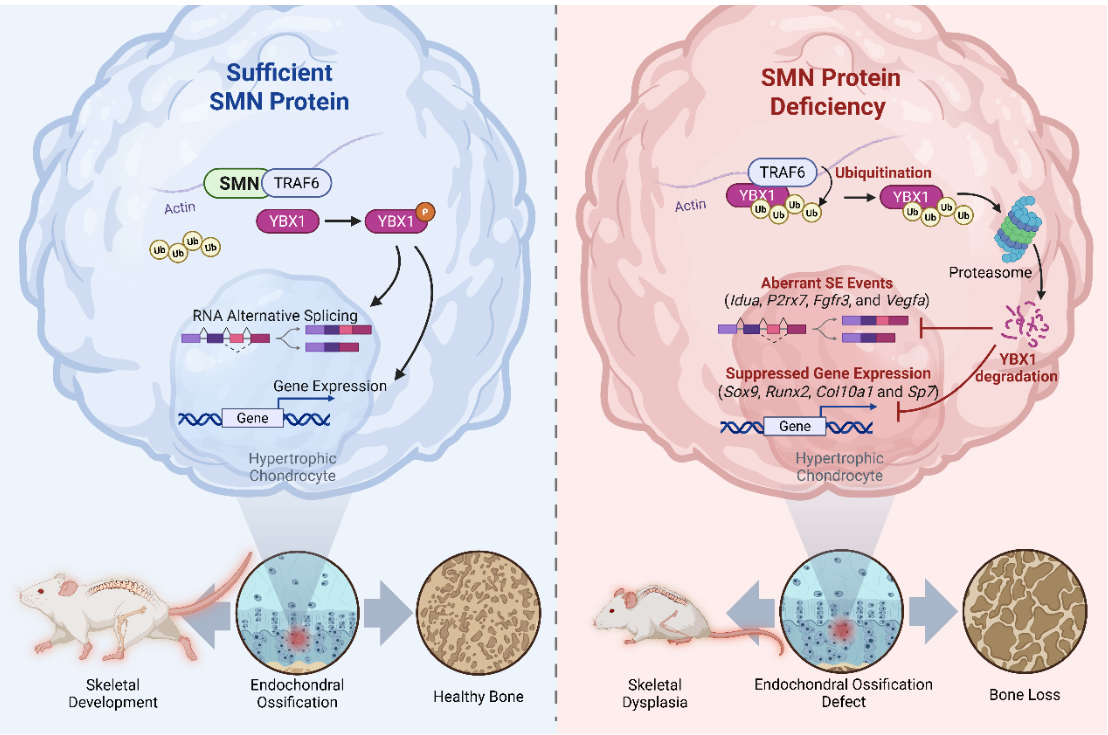

Graphic Abstract: Schematic illustration showing the possible mechanism by which SMN deficiency suppresses endochondral ossification. SMN deficiency promotes TRAF6-induced ubiquitination degradation of YBX1. Decreased YBX1 level leads to aberrant RNA alternative splicing and suppressed expression of genes involved in hypertrophic chondrocyte differentiation, resulting in endochondral ossification defects and ultimately causing skeletal dysplasia and bone loss.

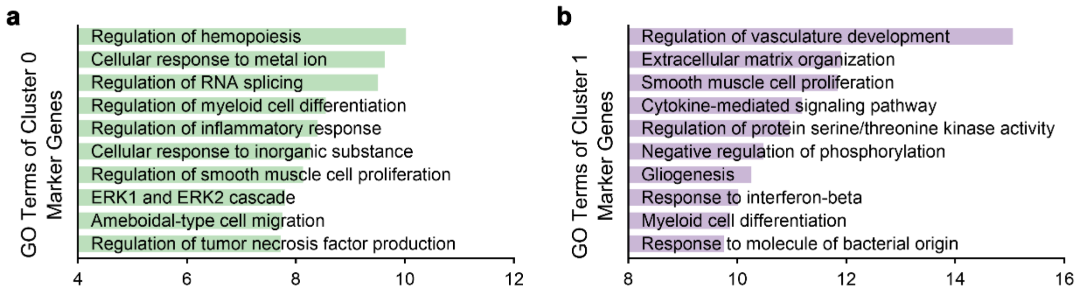

Supplemental Fig. 1 Different SSPC subclusters participate in various cellular biological processes.

(a and b) GO analysis of marker genes for Cluster 0 (a) and Cluster 1 (b) at 2 months, identified using Seurat 5 with a minimum expression percentage of 0.25 and a log fold change threshold of 0.25.

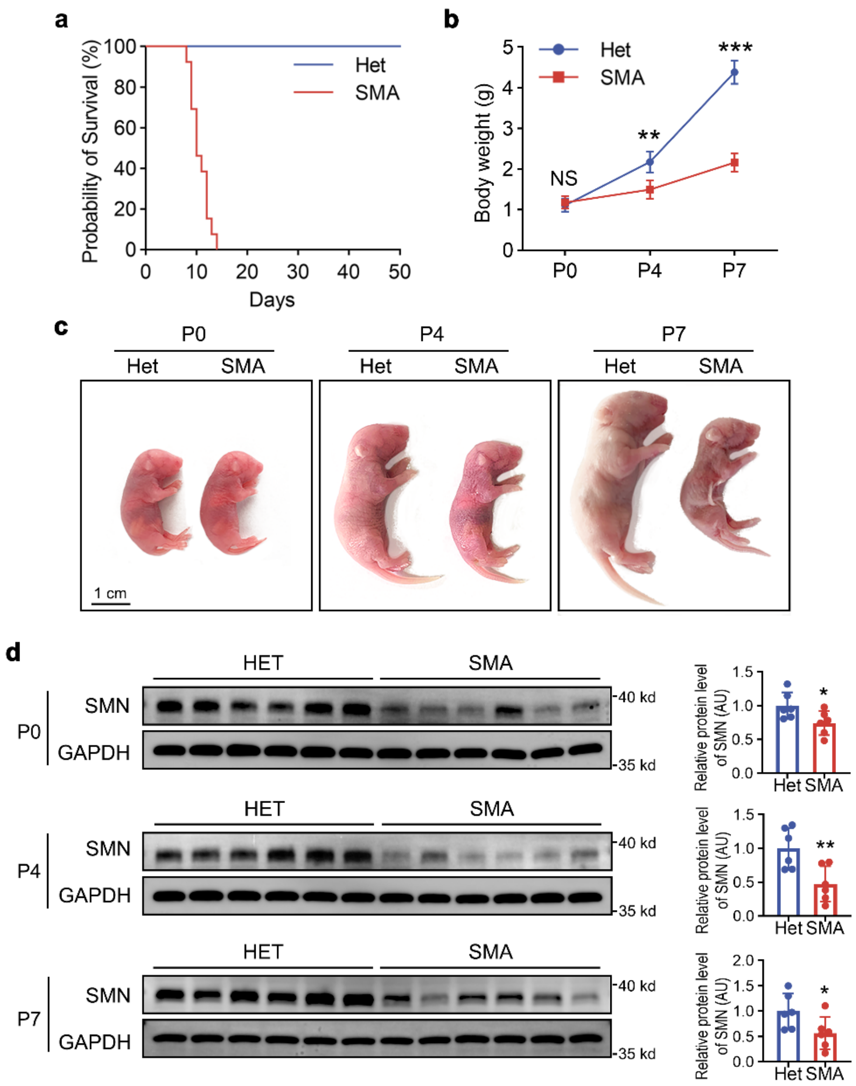

Supplemental Fig. 2 SMN is essential for survival and development of neonatal mice.

(a) Probability of survival of SMA and Het mice (n = 13 per group).

(b) Weight of SMA mice and Het littermates at P0, P4, and P7 (n = 3 per group). p-value was derived from Wilcoxon rank-sum test.

(c) General inspection of SMA and Het mice at P0, P4 and P7.

(d) Western blot analysis of SMN in growth plate cartilages from SMA and Het mice (n = 6 per group). GAPDH was used as the loading control and results were relative to the Het group. p-value was derived from two-tailed unpaired Student's t-test. Data are presented as the mean ± SD. $^{*}p < 0.05$, $^{**}p < 0.01$, $^{***}p < 0.001$.

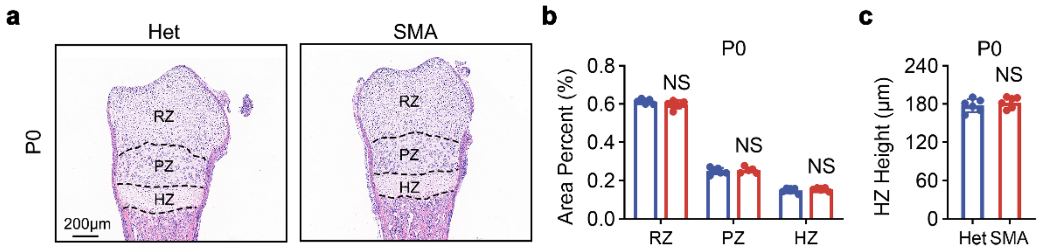

Supplemental Fig. 3 SMA mice show no abnormalities in growth plate anatomy at birth.

(a) HE staining of growth plates from SMA and Het mice at P0.

(b) Histograms show the ratio of different areas in growth plate from SMA and Het mice at P0 (n = 6 per group). p-value was derived from two-tailed unpaired Student's t-test.

(c) Histograms show HZ height in growth plates from SMA and Het mice at P0 (n = 6 per group). p-value was derived from two-tailed unpaired Student's t-test. Data are presented as the mean ± SD. $^{*}p < 0.05$, $^{**}p < 0.01$, $^{***}p < 0.001$.

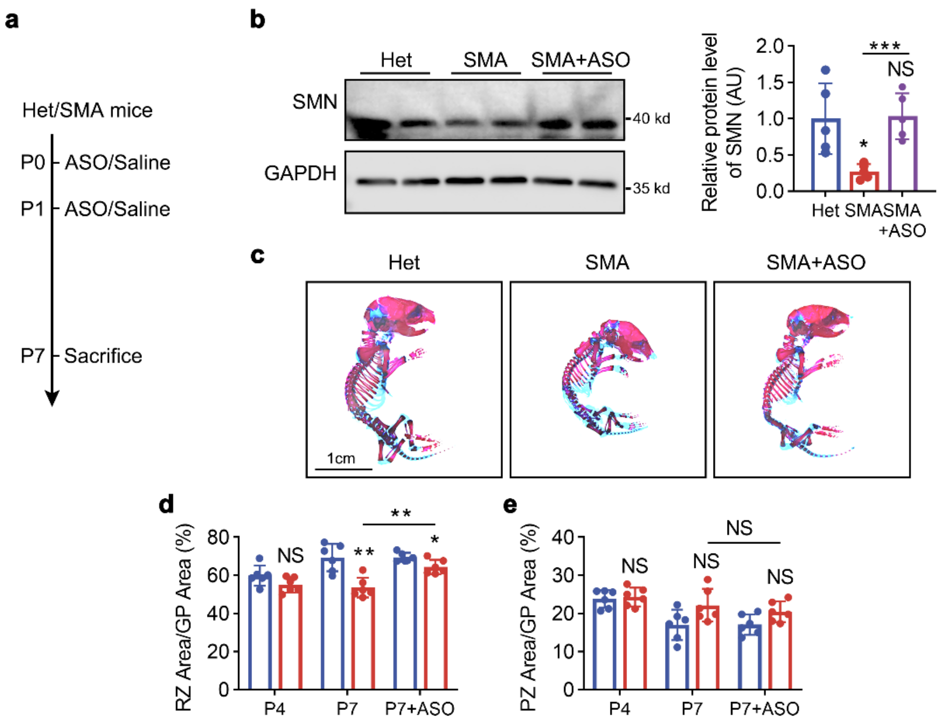

Supplemental Fig. 4 Increasing SMN protein with ASO10-29 rescues bone development defects.

(a) Experimental design involves the subcutaneous injection of ASO10-29 at a dose of 90 mg/kg, twice between P0 and P1.

(b) Western blotting of SMN in cartilages from HET, SMA, and SMA mice treated with ASO10-29 at P7 ($n = 5$ per group). GAPDH was used as the loading control and results were relative to the Het group. p-value was derived from Wilcoxon rank-sum test.

(c) Alcian blue and alizarin red double staining of skeletons from Het, SMA, and SMA mice treated with ASO10-29 at P7.

(d and e) Histograms showing ratio of RZ area (d) and PZ area (e) in growth plates (n = 6 per group) from HET, SMA, and SMA mice treated with ASO10-29 at P7. p-value was derived from two-tailed unpaired Student's t-test. Data are presented as the mean ± SD. $^{*}p < 0.05$, $^{**}p < 0.01$, $^{***}p < 0.001$.

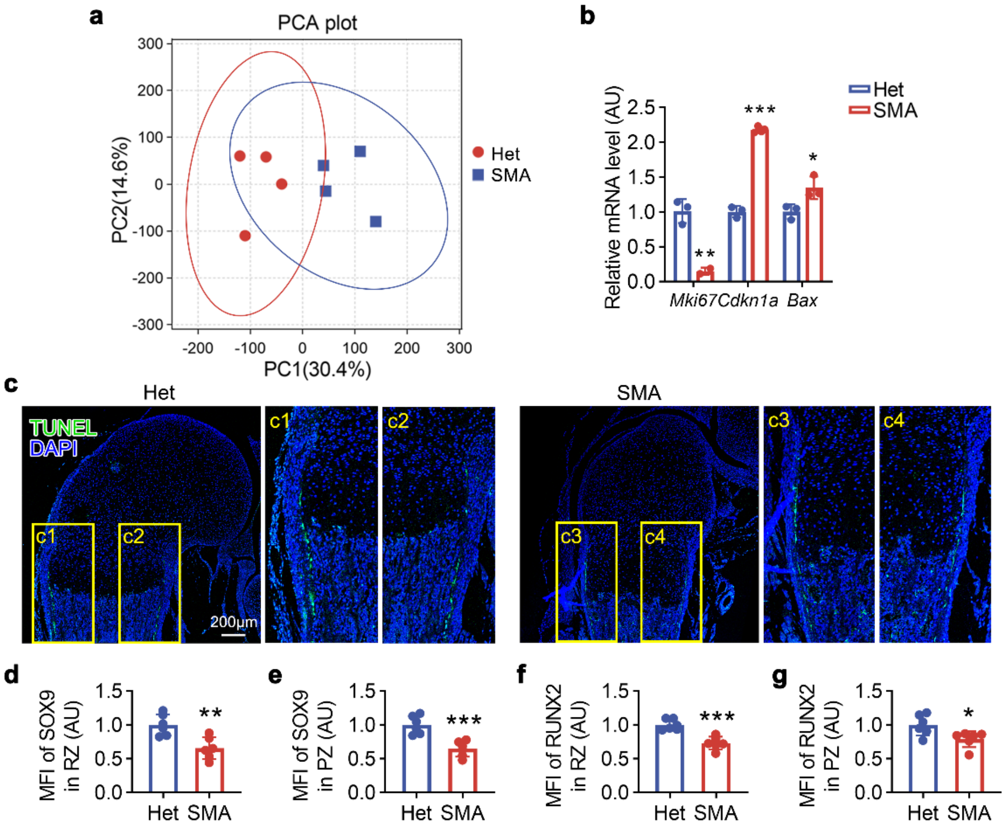

Supplemental Fig. 5 SMN deficiency suppresses endochondral ossification progression.

(a) PCA analysis of cartilage samples from SMA and Het cartilages on P4 (n = 4 per group).

(b) Relative mRNA levels of *Mki67*, *Cdkn1a*, and *Bax* in growth plates from SMA and Het mice at P4 (n = 3 per group). *Gapdh* was used as the loading control and results were relative to the Het group. p-value was derived from Wilcoxon rank-sum test.

(c) Immunofluorescence image of TUNEL staining in growth plate of Het and SMA mice at P9. Yellow boxes indicate the magnified TUNEL-positive regions in the periosteum adjacent to the hypertrophic zone.

(d-g) Histograms showing MFI of SOX9 (d and e) and RUNX2 (f and g) in RZ and PZ (n = 6 per group). Results were relative to the Het group. p-value was derived from two-tailed unpaired Student's t-test. Data are presented as the mean ± SD. $^{*}p < 0.05$, $^{**}p < 0.01$, $^{***}p < 0.001$.

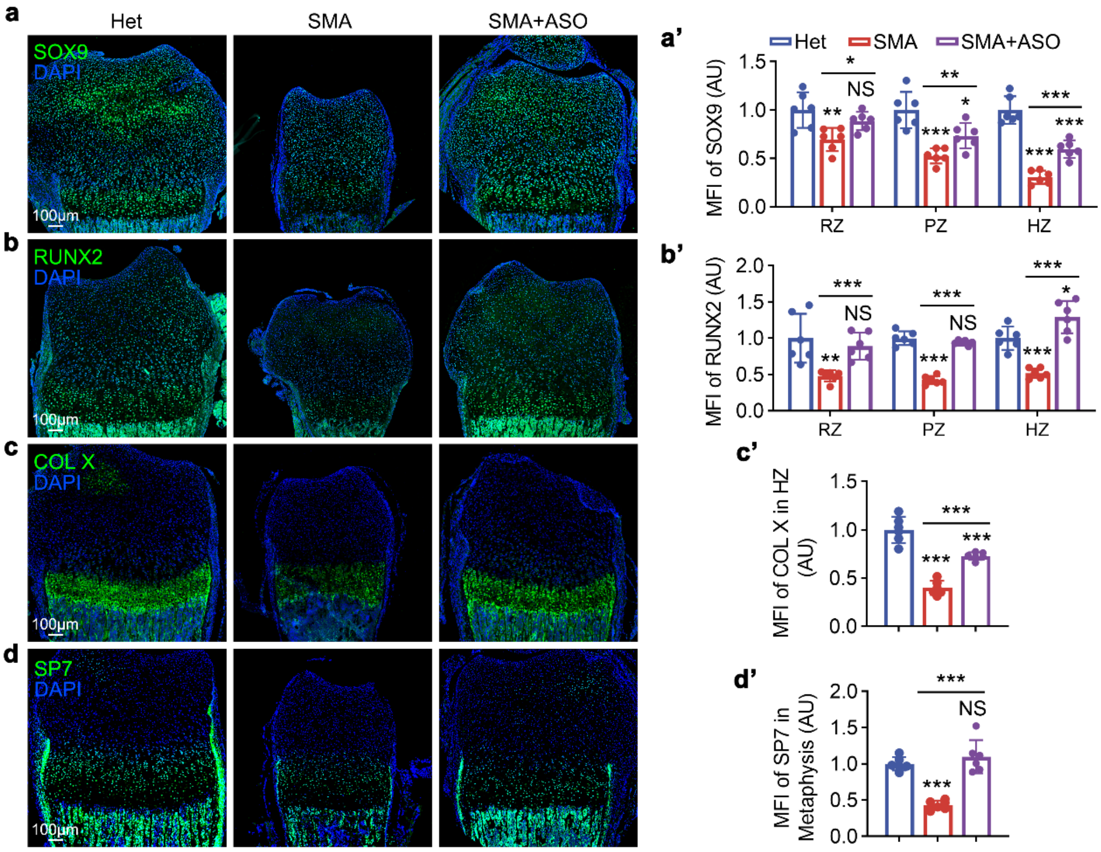

Supplemental Fig. 6 Increasing SMN protein levels rescues endochondral ossification defects.

(a-d) Immunofluorescence staining of SOX9 (a), RUNX2 (b), COL X (c), and SP7 (d) in growth plates from Het, SMA, and SMA mice treated with ASO10-29 on P7. Histograms show MFI of SOX9 (a'), and RUNX2 (b') in growth plates, COL X in HZ (c'), and SP7 in metaphysis (d') (n = 6 per group). Results were relative to the Het group. p-value was derived from two-tailed unpaired Student's t-test. Data are presented as the mean ± SD. $^{*}p < 0.05$, $^{**}p < 0.01$, $^{***}p < 0.001$.

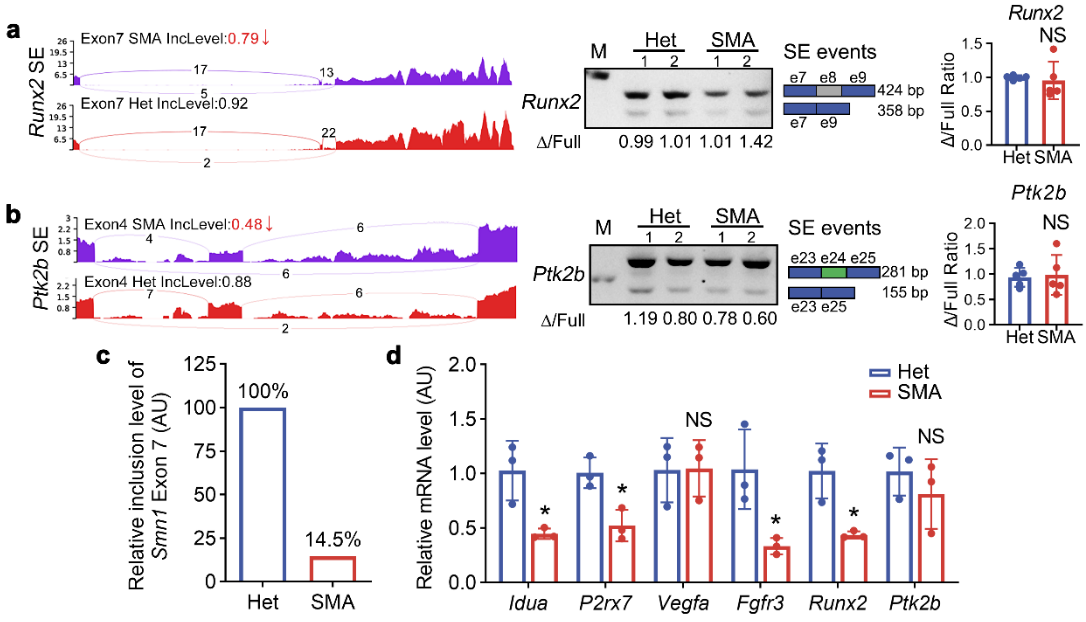

Supplemental Fig. 7 SMN deficiency dysregulates pre-mRNA alternative splicing and gene expression patterns.

(a and b) Sashimi plots of the different SE events for the genes *Runx2* (a) and *Ptk2b* (b). Semi-quantitative real-time PCR analysis of these genes in cartilages from SMA and Het mice was presented. Exon skipping ratios were relative to the Het group (n = 5 per group). p-value was derived from Wilcoxon rank-sum test.

(c) Histograms showing exon 7 inclusion level of *Smn1* in growth plate cartilage of SMA and HET mice. Results were relative to the Het group.

(d) Relative mRNA levels of *Idua*, *P2rx7*, *Vegfa*, *Fgfr3*, *Runx2*, and *Ptk2b* in growth plates from SMA and Het mice on P4 (n = 3 per group). *Gapdh* was used as the loading control and results were relative to the Het group. p-value was derived from Wilcoxon rank-sum test. Data are presented as the mean ± SD. $^{*}p < 0.05$, $^{**}p < 0.01$, $^{***}p < 0.001$.

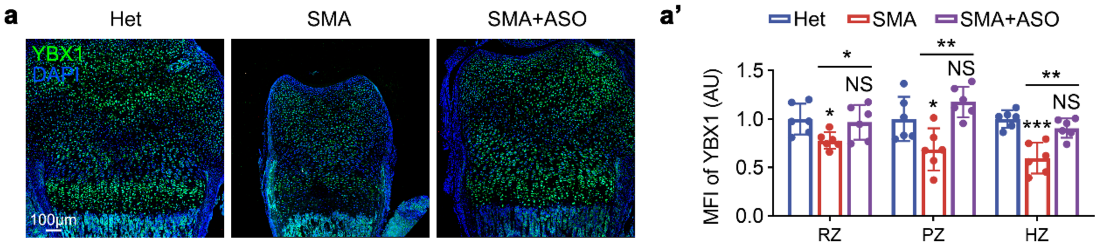

Supplemental Fig. 8 SMN deficiency leads to reduction of YBX1.

(a) Immunofluorescence staining of YBX1 in growth plates from Het, SMA, and SMA mice treated with ASO10-29 at P7. Histograms show MFI of YBX1 (a') in growth plates (n = 6 per group). Results were relative to the Het group. p-value was derived from two-tailed unpaired Student's t-test. Data are presented as the mean ± SD. $^{*}p < 0.05$, $^{**}p < 0.01$, $^{***}p < 0.001$.

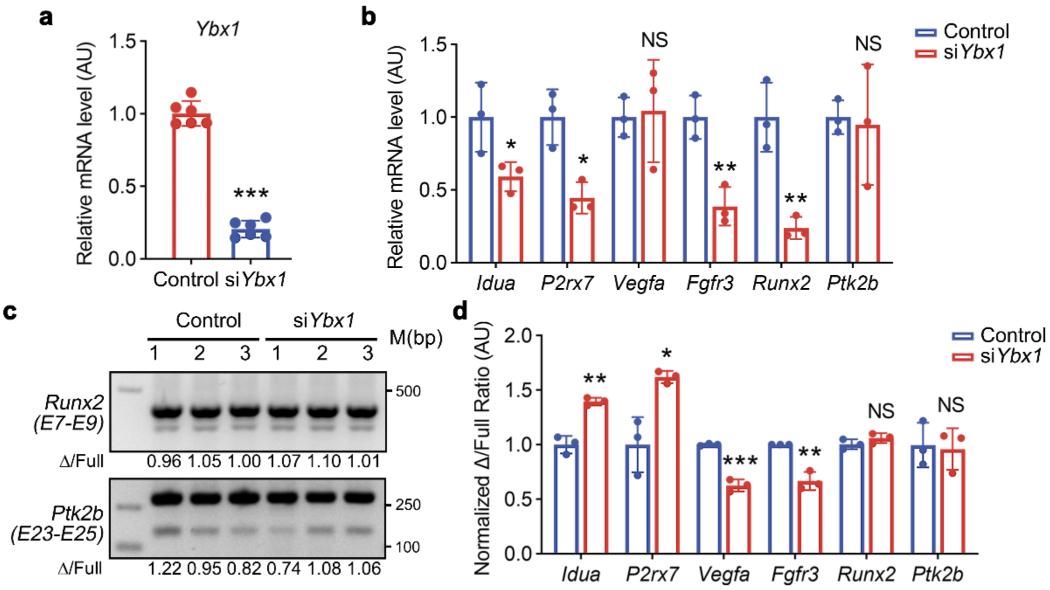

Supplemental Fig. 9 YBX1 knockdown induces SMA-like phenotypes in chondrocytes.

(a) Relative mRNA levels of *Ybx1* in ATDC5 cells transfected with siCtrl and si*Ybx1* (n = 6 per group). *Gapdh* was used as the loading control and results were relative to the control group. p-value was derived from two-tailed unpaired Student's t-test.

(b) Relative mRNA levels of *Idua*, *P2rx7*, *Vegfa*, *Fgfr3*, *Runx2*, and *Ptk2b* in ATDC5 cells transfected with siCtrl and si*Ybx1* (n = 3 per group). *Gapdh* was used as the loading control and results were relative to the control group. p-value was derived from Wilcoxon rank-sum test.

(c and d) Semi-quantitative real-time PCR analysis of *Runx2* and *Ptk2b* in ATDC5 cells transfected with siCtrl or si*Ybx1* (c). Exon deletion ratio was relative to the control group (n = 3 per group) (d). p-value was derived from Wilcoxon rank-sum test. Data are presented as the mean ± SD. $^{*}p < 0.05$, $^{**}p < 0.01$, $^{***}p < 0.001$.

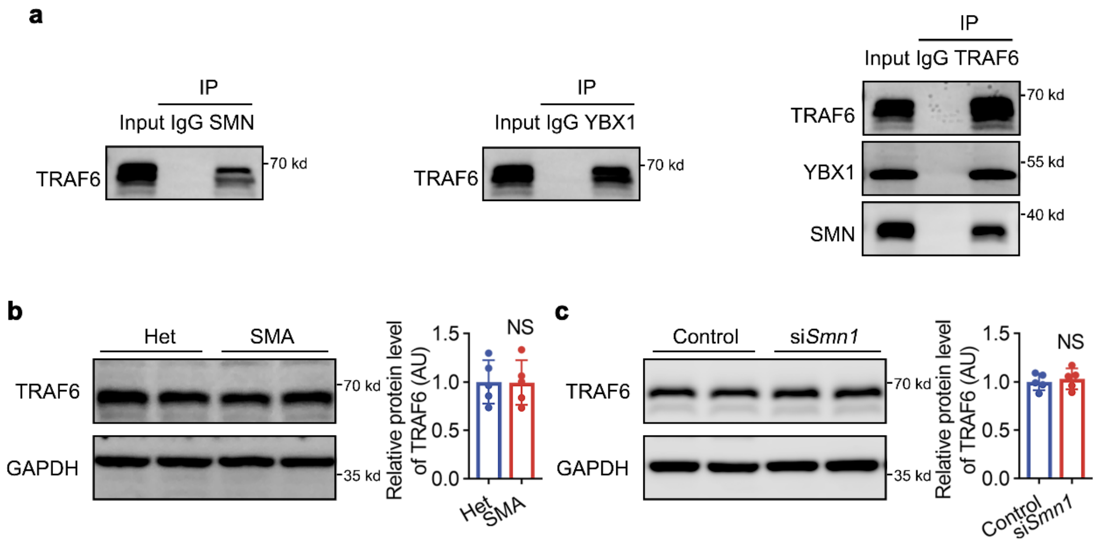

Supplemental Fig. 10 SMN deficiency does not affect TRAF6 protein levels.

(a) Co-IP analysis of the interaction between SMN, YBX1, and TRAF6 in ATDC5 cells. **(b and c)** Western blot analysis of TRAF6 in cartilages from SMA and Het mice at P4 (n = 5 per group) (b) and ATDC5 cells transfected with siCtrl and si*Smn1* (n = 5 per group) (c). GAPDH was used as the loading control and results were relative to the Het or control group. p-value was derived from Wilcoxon rank-sum test. Data are presented as the mean ± SD. $^{*}p < 0.05$, $^{**}p < 0.01$, $^{***}p < 0.001$.

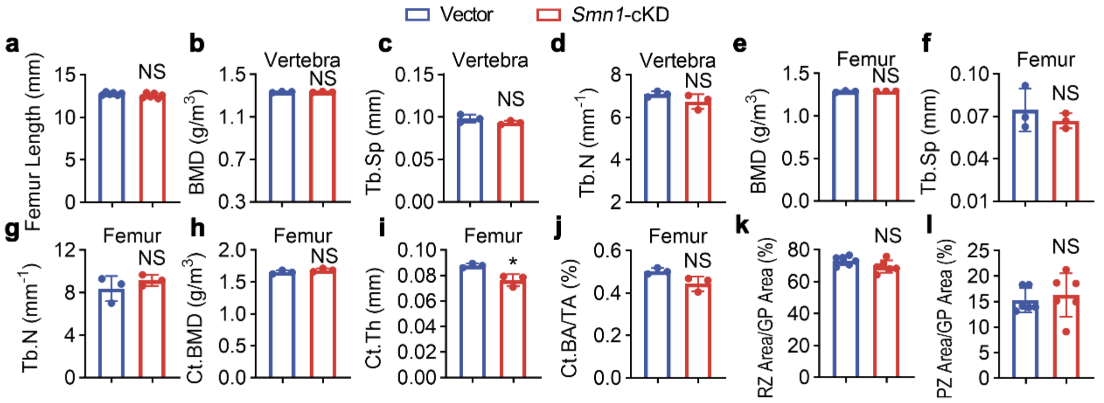

Supplemental Fig. 11 *Smn1* cKD in chondrocytes leads to decreased bone quality.

(a) Histogram showing length of femurs of Vector and *Smn1*-cKD mice at P12 ($n = 6$ per group). *p*-value was derived from two-tailed unpaired Student's t-test.

(b-g) Quantitative analysis of trabecular bone microarchitecture (BMD, Tb.Sp, and Tb.N) in vertebra (b-d) and femur (e-g) from Vector and *Smn1*-cKD mice at P12 ($n = 3$ per group). *p*-value was derived from Wilcoxon rank-sum test.

(h-j) Quantitative analysis of cortical bone microarchitecture (Ct.BMD, Ct.Th, and Ct.BA/TA) in femur from Vector and *Smn1*-cKD mice at P12 ($n = 3$ per group). *p*-value was derived from Wilcoxon rank-sum test.

(k and l) Histograms showing ratio of RZ area (k) and PZ area (l) in growth plates ($n = 6$ per group) from Vector and *Smn1*-cKD mice at P12 ($n = 6$ per group). *p*-value was derived from two-tailed unpaired Student's t-test. Data are presented as the mean ± SD. $^{*}p < 0.05$, $^{**}p < 0.01$, $^{***}p < 0.001$.

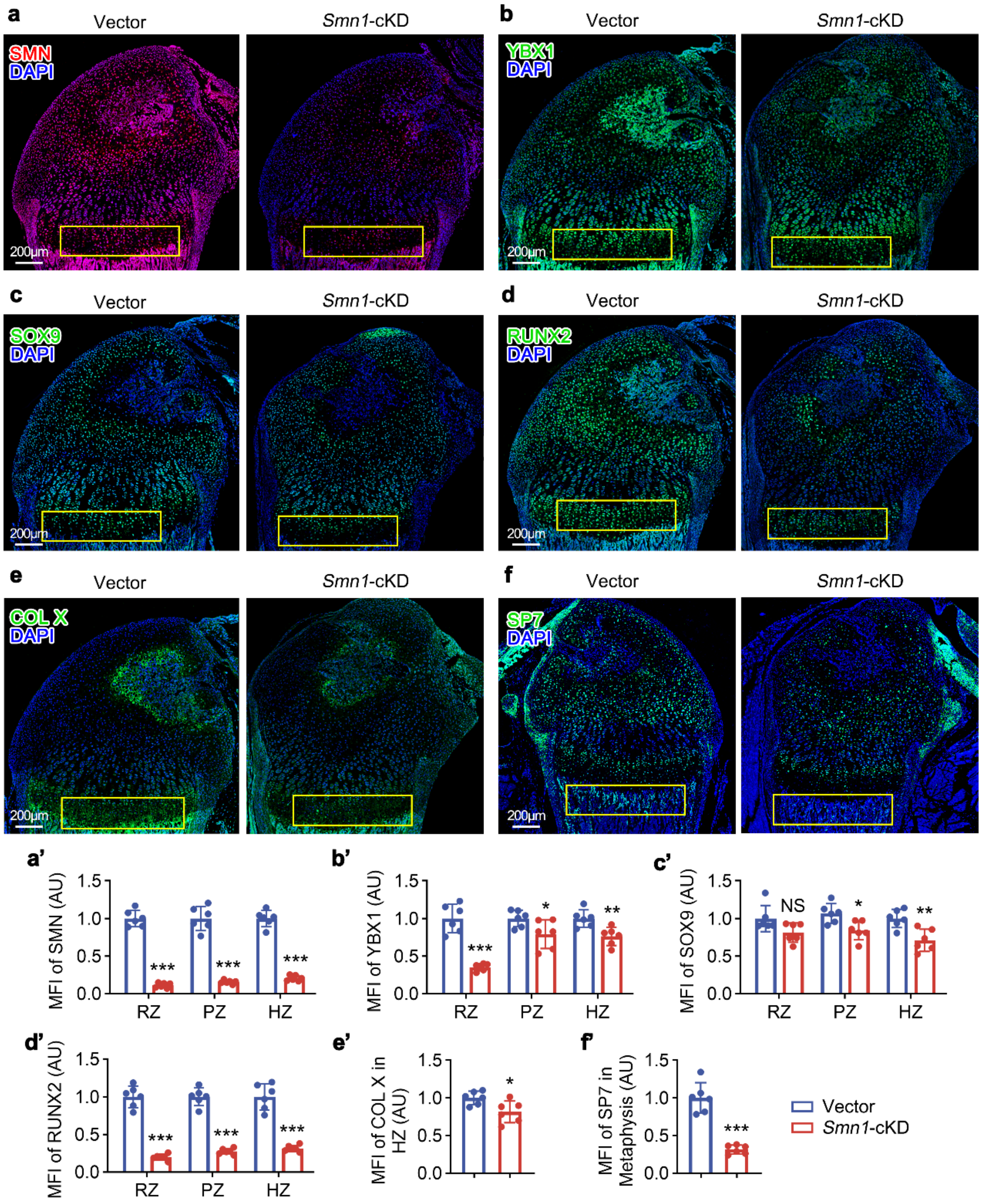

Supplemental Fig. 12 *Smn1*-cKD in chondrocytes caused retardation of endochondral ossification.

(a-f) Immunofluorescence staining of SMN (a), YBX1 (b), SOX9 (c), RUNX2 (d), COL X (e), and SP7 (f) in growth plates from Vector and *Smn1*-cKD mice at P12. Histograms show MFI of SMN (a'), YBX1 (b'), SOX9 (c') and RUNX2 (d') in growth plates, COL X in HZ (e'), and SP7 in metaphysis (f') (n = 6 per group). Results were relative to the Vector group. p-value was derived from two-tailed unpaired Student's t-test. Data are presented as the mean ± SD. $^{*}p < 0.05$, $^{**}p < 0.01$, $^{***}p < 0.001$.

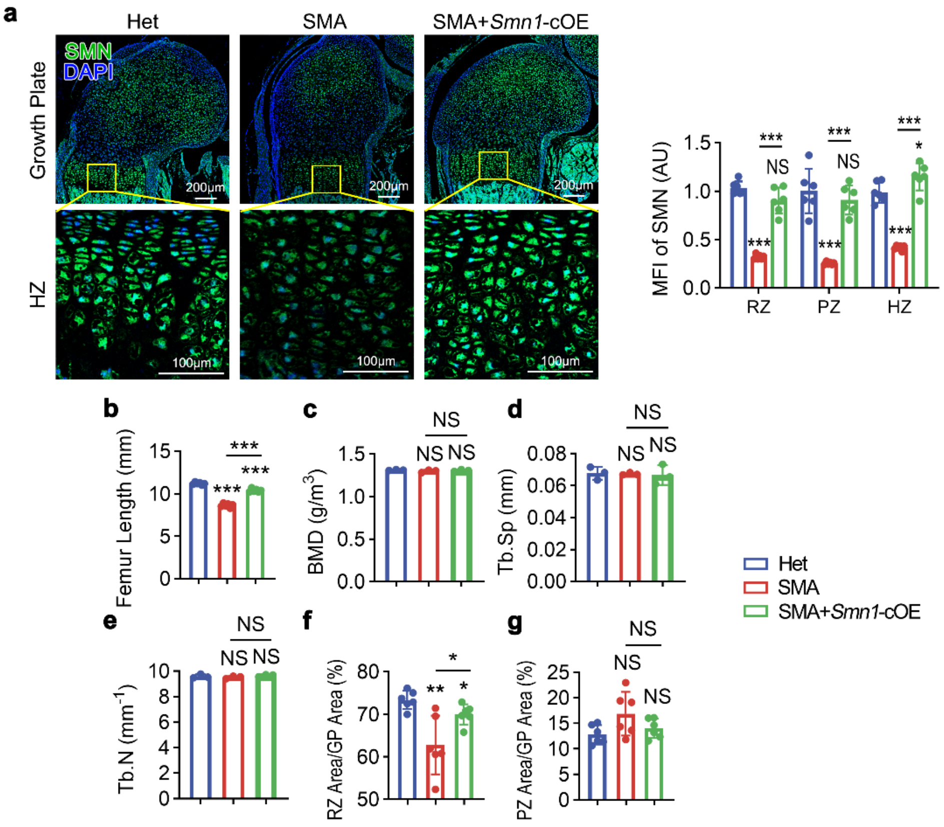

Supplemental Fig. 13 Chondrocyte-specific SMN restoration partially reverses skeletal abnormalities in SMA mice.

(**a**) Immunofluorescence staining of SMN in growth plates from Het, SMA and *Smn1*-cOE mice at P9. The regions outlined with yellow boxes in the upper panels are shown at higher magnification in the corresponding lower panels. Histograms show MFI of SMN in growth plates ($n = 6$ per group). Results were relative to the Het group. p-value was derived from two-tailed unpaired Student's t-test.

(b) Histogram showing length of femurs from Het, SMA and *Smn1*-cOE mice at P9 ($n = 6$ per group). p-value was derived from two-tailed unpaired Student's t-test.

(c-e) Quantitative analysis of trabecular bone microarchitecture (BMD, Tb.Sp, and Tb.N) in femurs from Het, SMA and *Smn1*-cOE mice at P9 ($n = 3$ per group). p-value was derived from Wilcoxon rank-sum test.

(f and g) Histograms showing ratio of RZ area (f) and PZ area (g) in growth plates ($n = 6$ per group) from Het, SMA and *Smn1*-cOE mice at P9 ($n = 6$ per group). p-value was derived from two-tailed unpaired Student's t-test. Data are presented as the mean ± SD. $^{*}p < 0.05$, $^{**}p < 0.01$, $^{***}p < 0.001$.

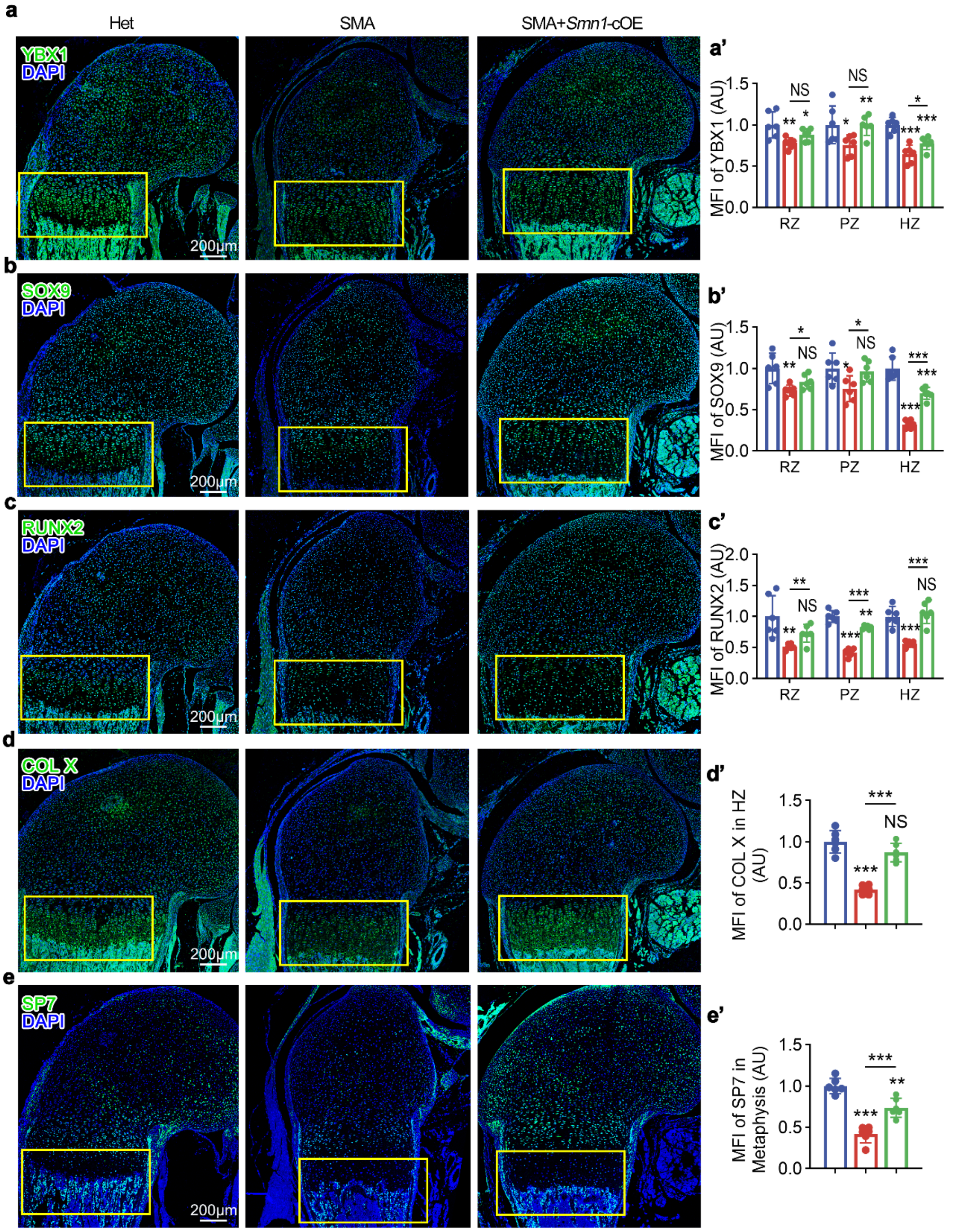

Supplemental Fig. 14 Chondrocyte-specific SMN restoration partially reverses endochondral ossification defects in SMA mice.

(a-e) Immunofluorescence staining of YBX1 (a), SOX9 (b), RUNX2 (c), COL X (d), and SP7 (e) in growth plates from Het, SMA and *Smn1*-cOE mice at P9. Histograms show MFI of YBX1 (a'), SOX9 (b') and RUNX2 (c') in growth plates, COL X in HZ (d'), and SP7 in metaphysis (e') (n = 6 per group). Results were relative to the Het group. p-value was derived from two-tailed unpaired Student's t-test. Data are presented as the mean ± SD. $^{*}p < 0.05$, $^{**}p < 0.01$, $^{***}p < 0.001$.
