# Supplementary material for: SMN deficiency inhibits endochondral ossification via promoting TRAF6-induced ubiquitination degradation of YBX1 in spinal muscular atrophy
Source: Bone Res. 2025 Dec 1;13:97. doi: 10.1038/s41413-025-00473-6 (PMC12665793; doi:10.1038/s41413-025-00473-6)
Supplement: Supplementary file 2 — Unedited blot and gel images [file 41413_2025_473_MOESM2_ESM.pdf]

Unedited gel images

Figure 4e and 4h

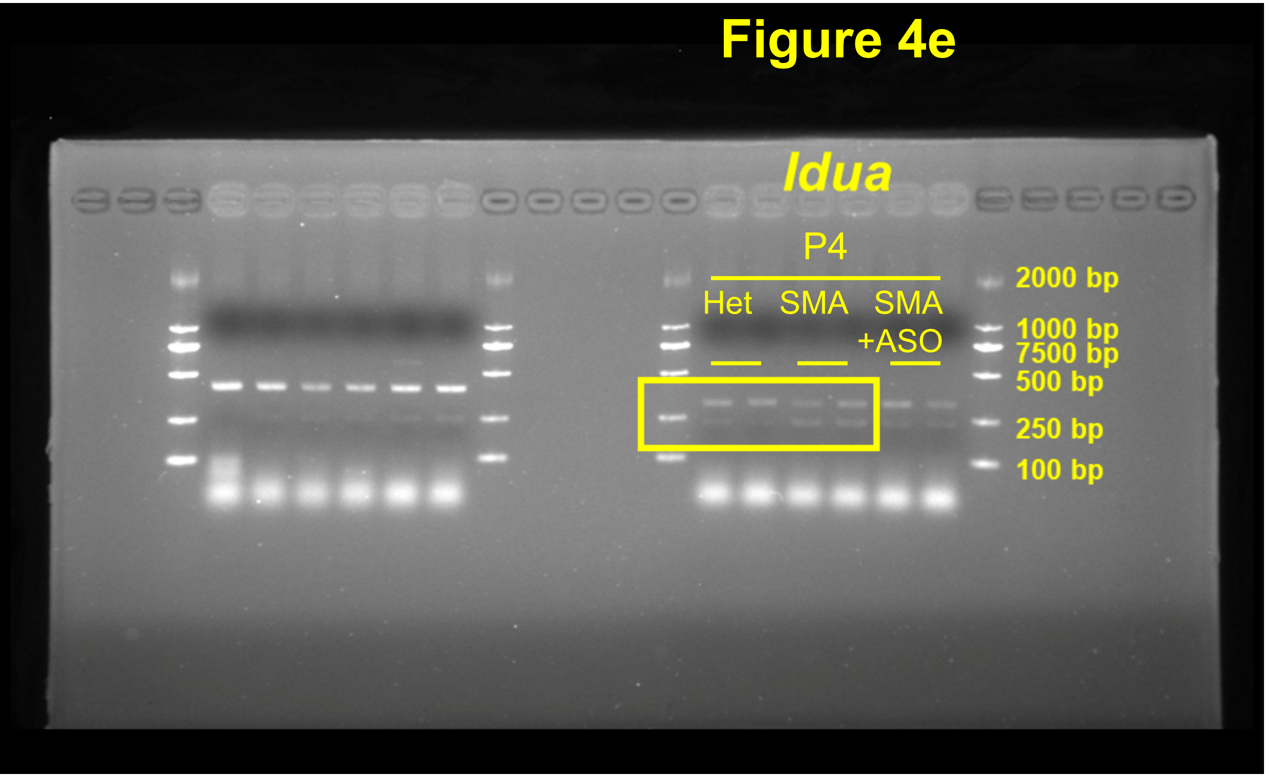

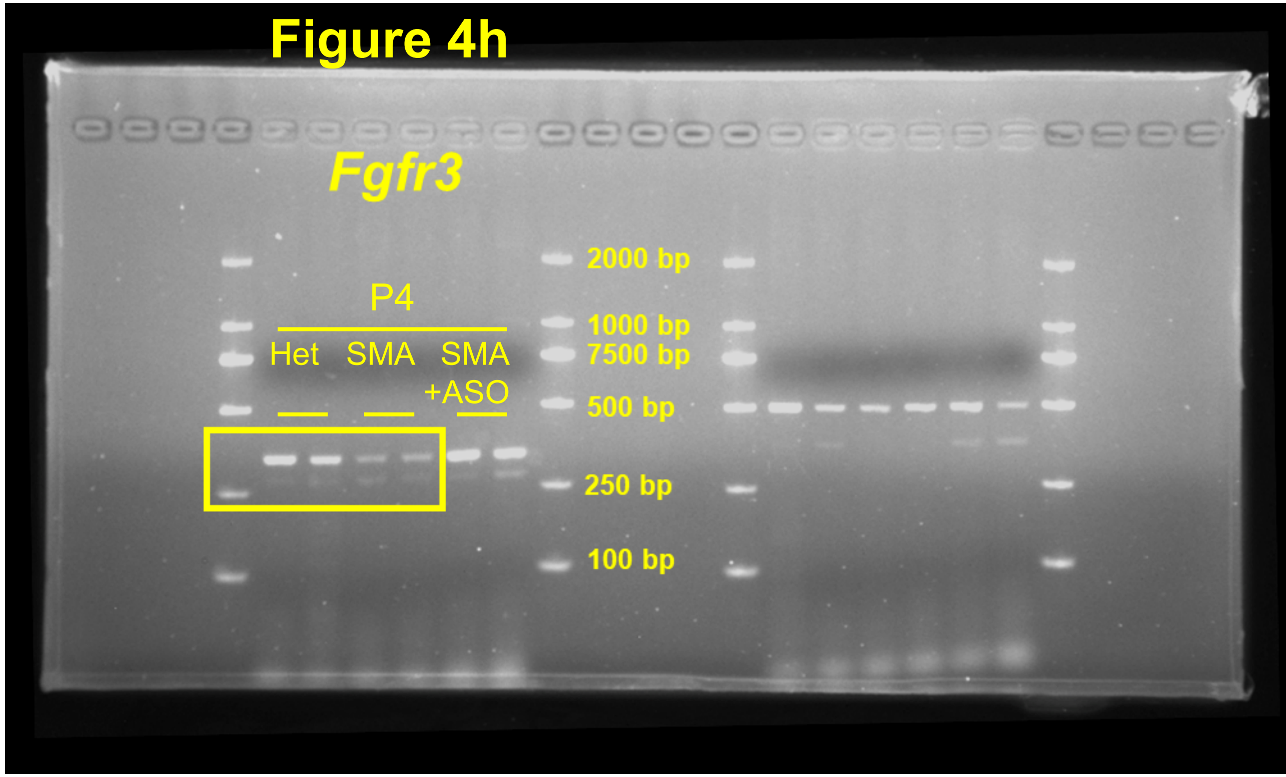

Yellow box indicates the region used for Figure 4e and 4h in the manuscript.

Unused lanes represent other experimental conditions or detection targets not included in this study.

Figure 4f and 4g

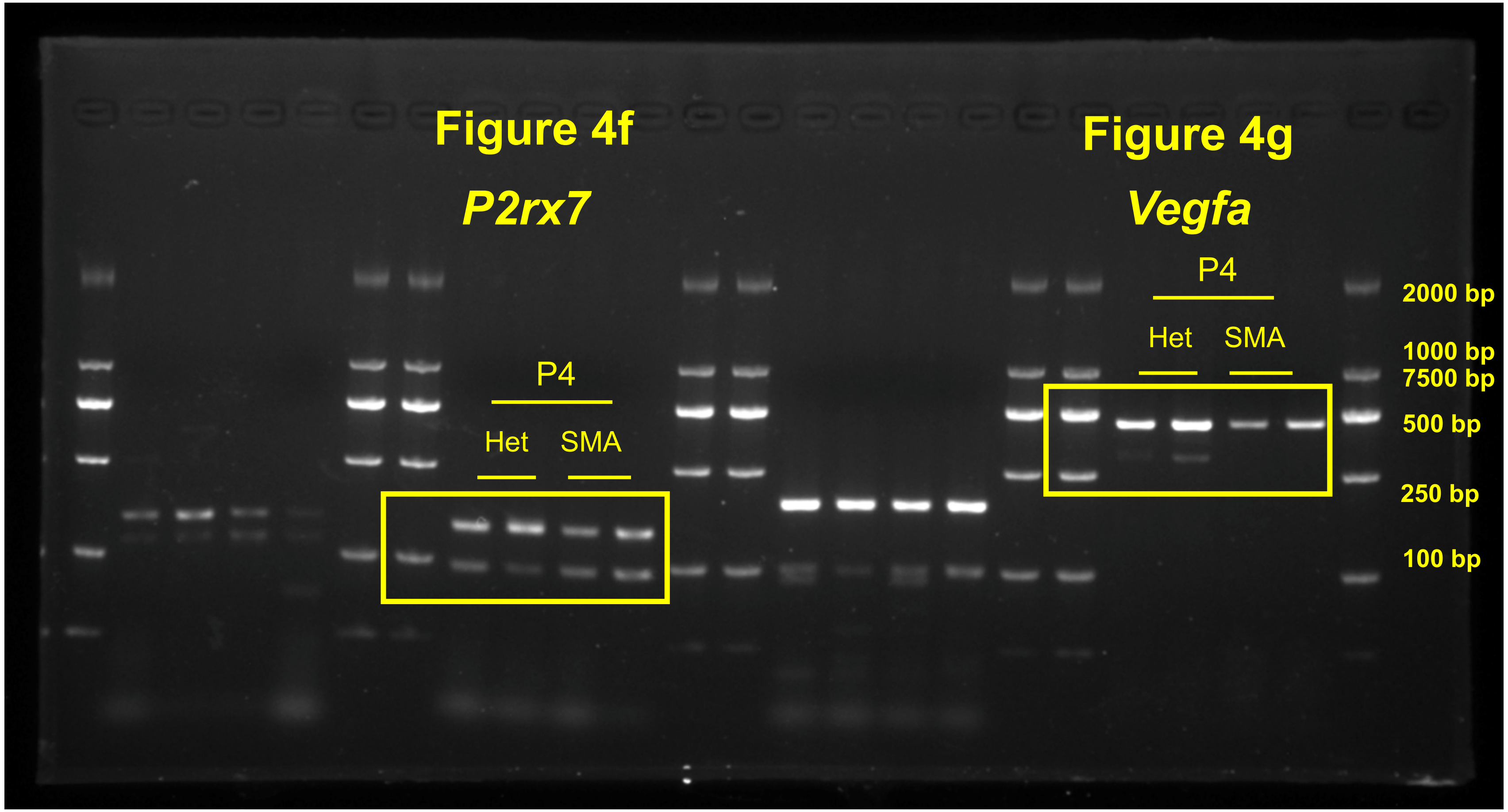

Yellow box indicates the region used for Figure 4f and 4g in the manuscript.

Unused lanes represent other experimental conditions or detection targets not included in this study.

Figure 4i

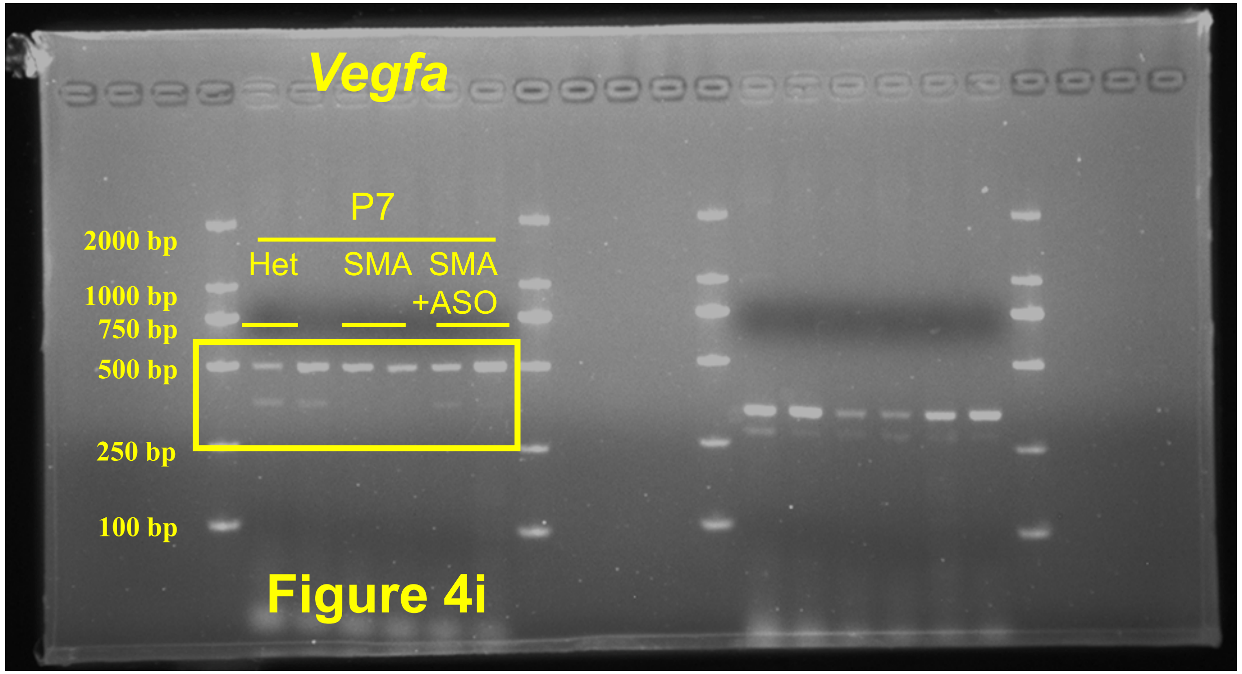

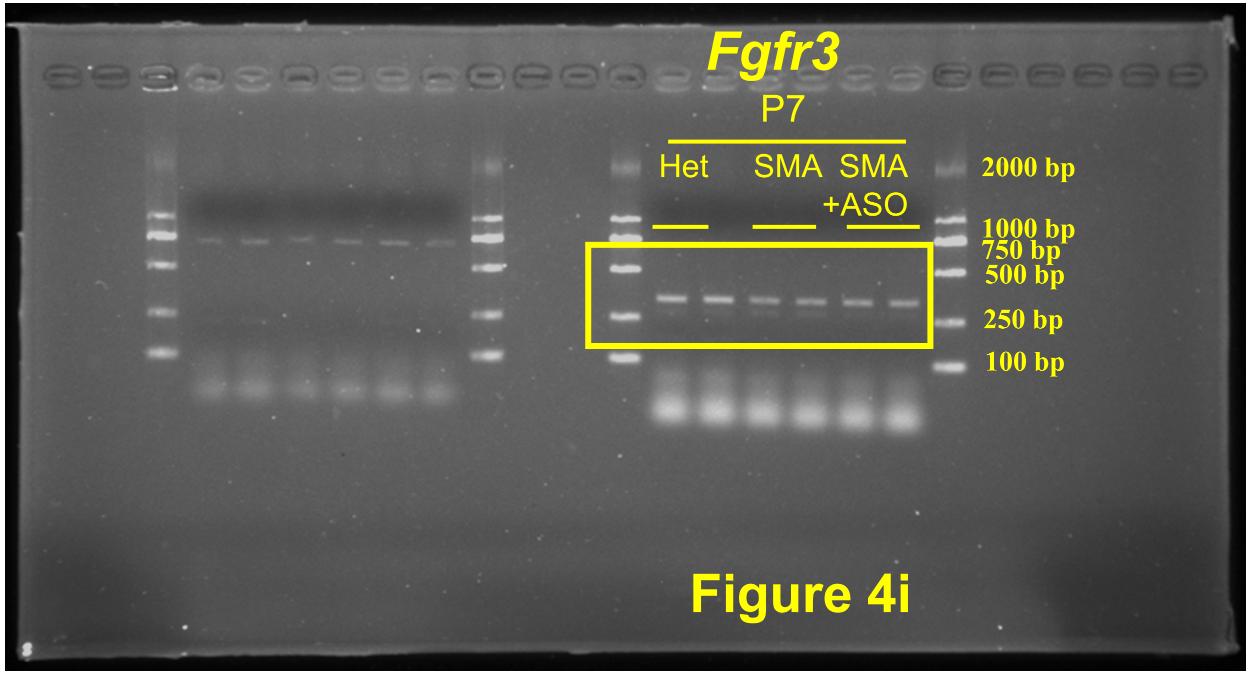

Yellow box indicates the region used for Figure 4i in the manuscript.

Unused lanes represent other experimental conditions or detection targets not included in this study.

Figure 4i

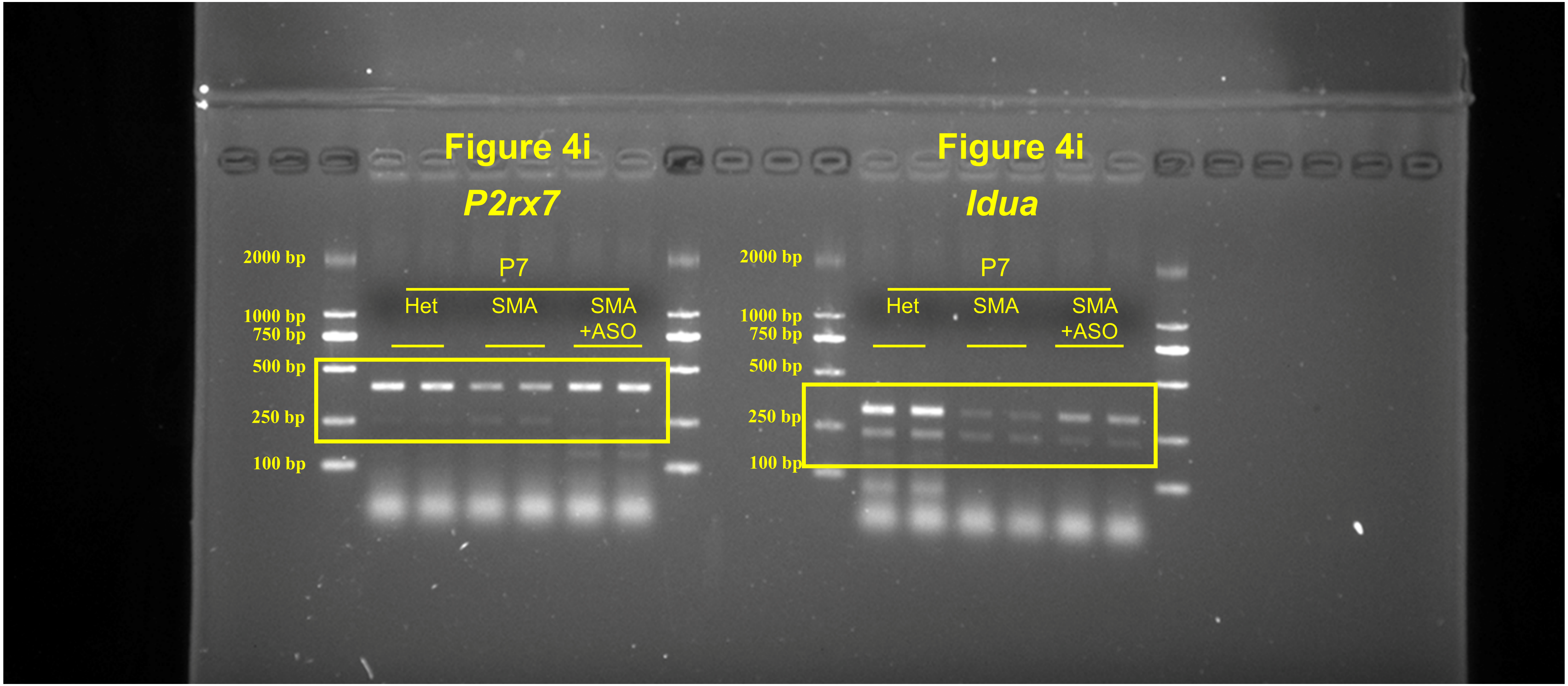

Yellow box indicates the region used for Figure 4i in the manuscript.

Unused lanes represent other experimental conditions or detection targets not included in this study.

Figure 6b

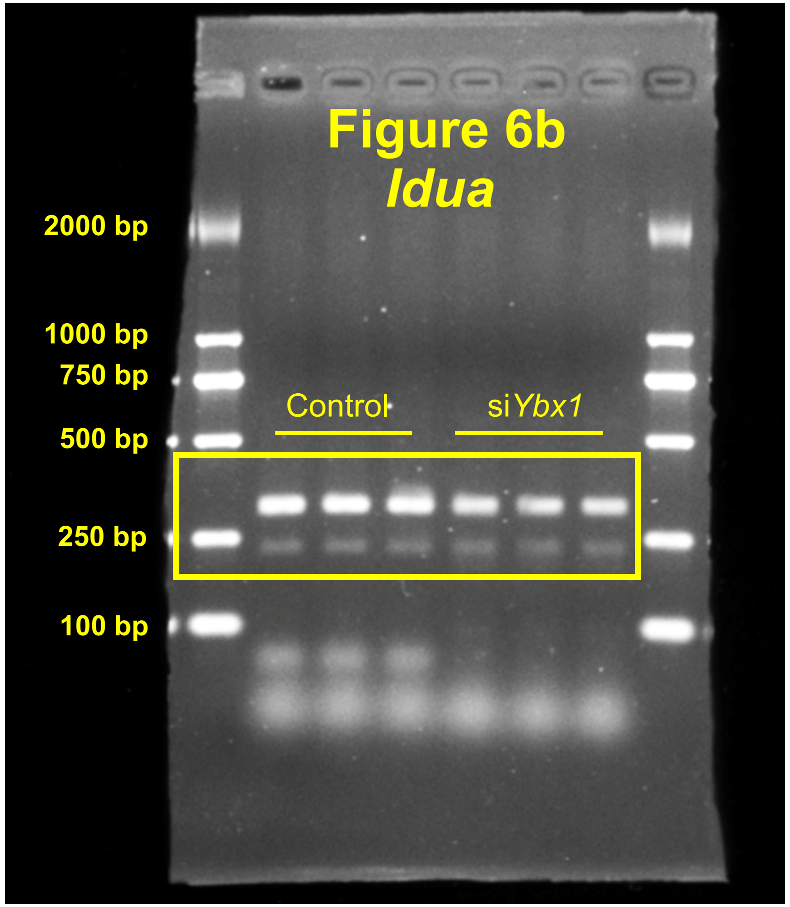

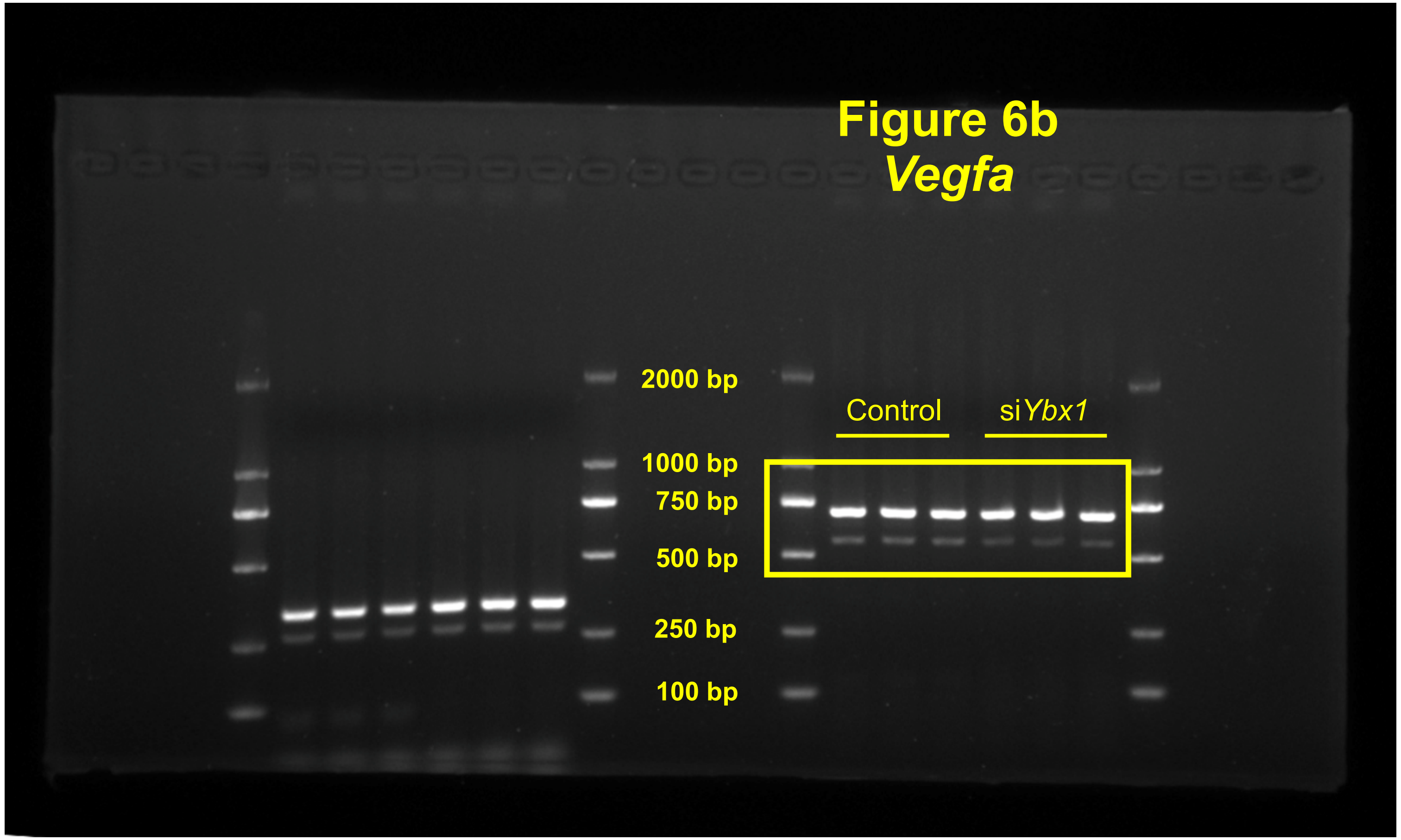

Yellow box indicates the region used for Figure 6b in the manuscript.

Unused lanes represent other experimental conditions or detection targets not included in this study.

Figure 6b

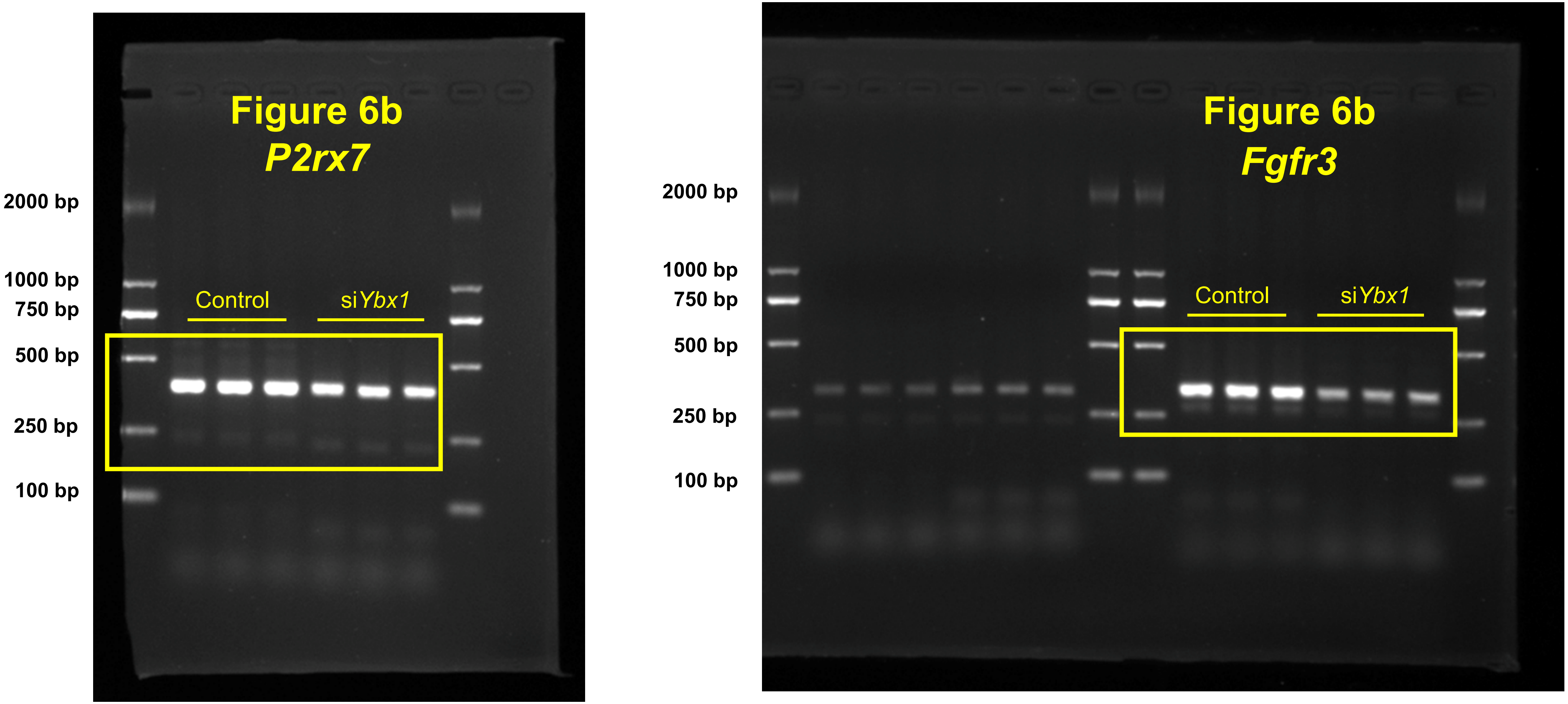

Yellow box indicates the region used for Figure 6b in the manuscript.

Unused lanes represent other experimental conditions or detection targets not included in this study.

Figure S7a

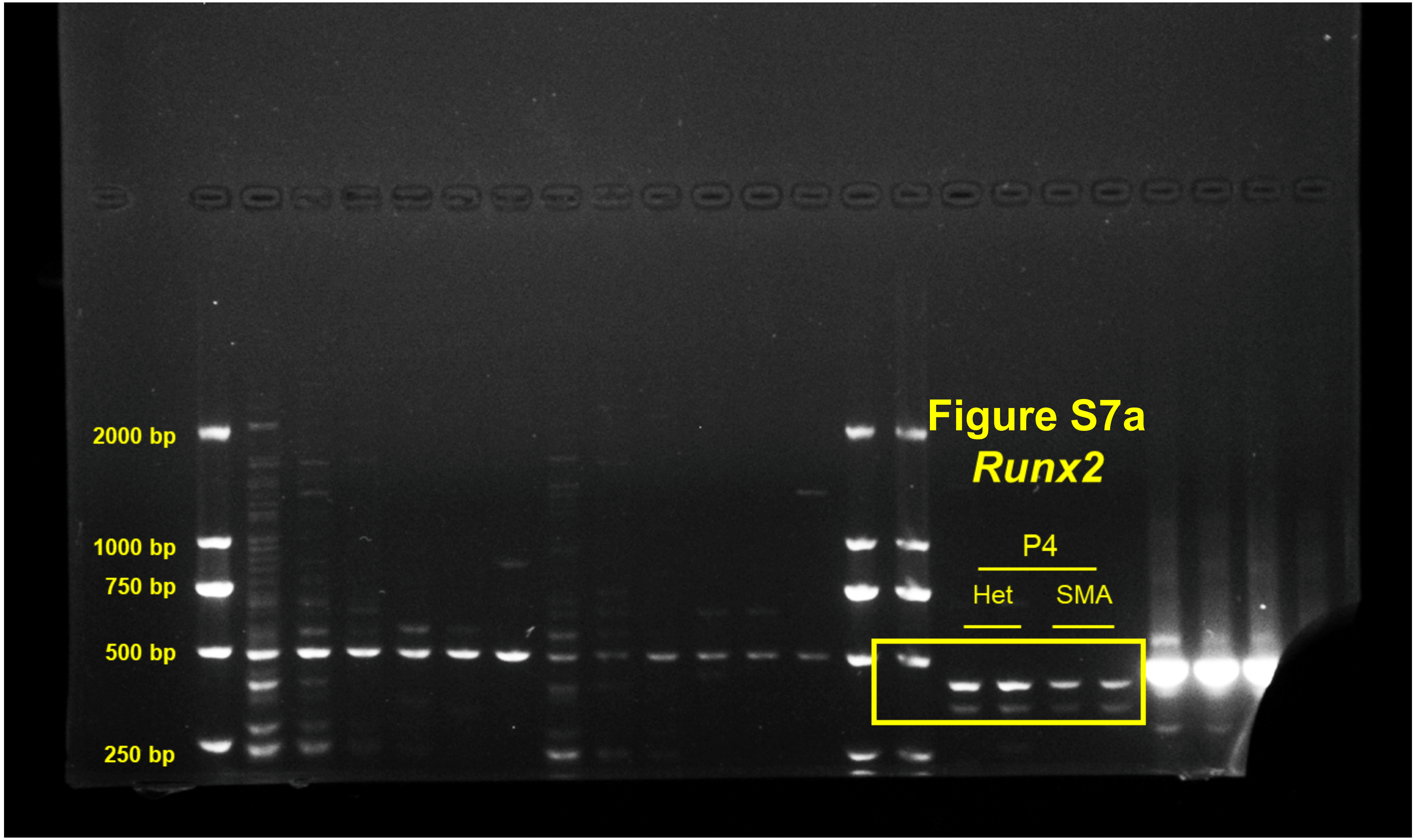

Yellow box indicates the region used for Figure S7a in the manuscript.

Unused lanes represent other experimental conditions or detection targets not included in this study.

Figure S7b

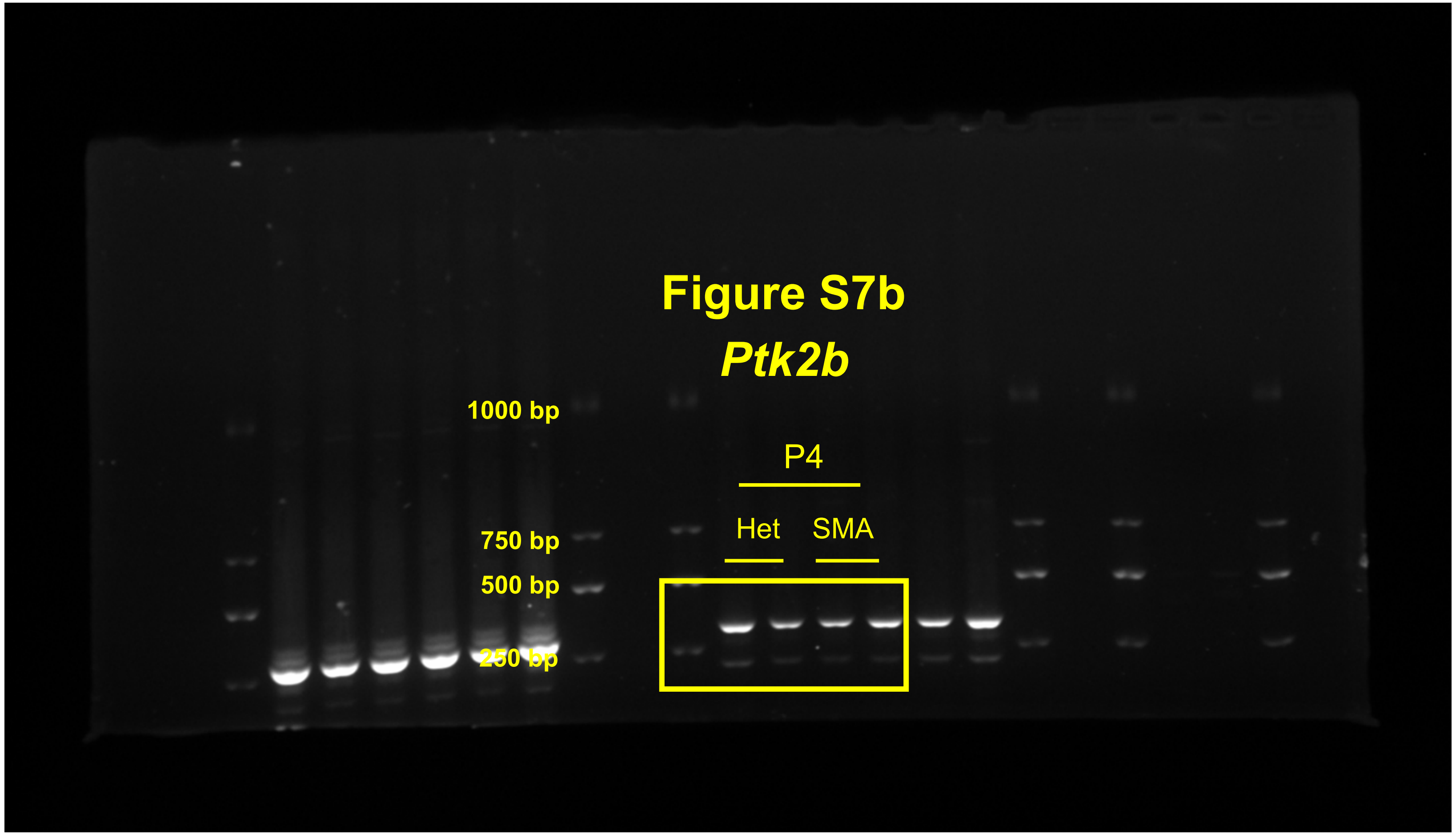

Yellow box indicates the region used for Figure S7b in the manuscript.

Unused lanes represent other experimental conditions or detection targets not included in this study.

Figure S9c

Yellow box indicates the region used for Figure S9c in the manuscript.

Unused lanes represent other experimental conditions or detection targets not included in this study.

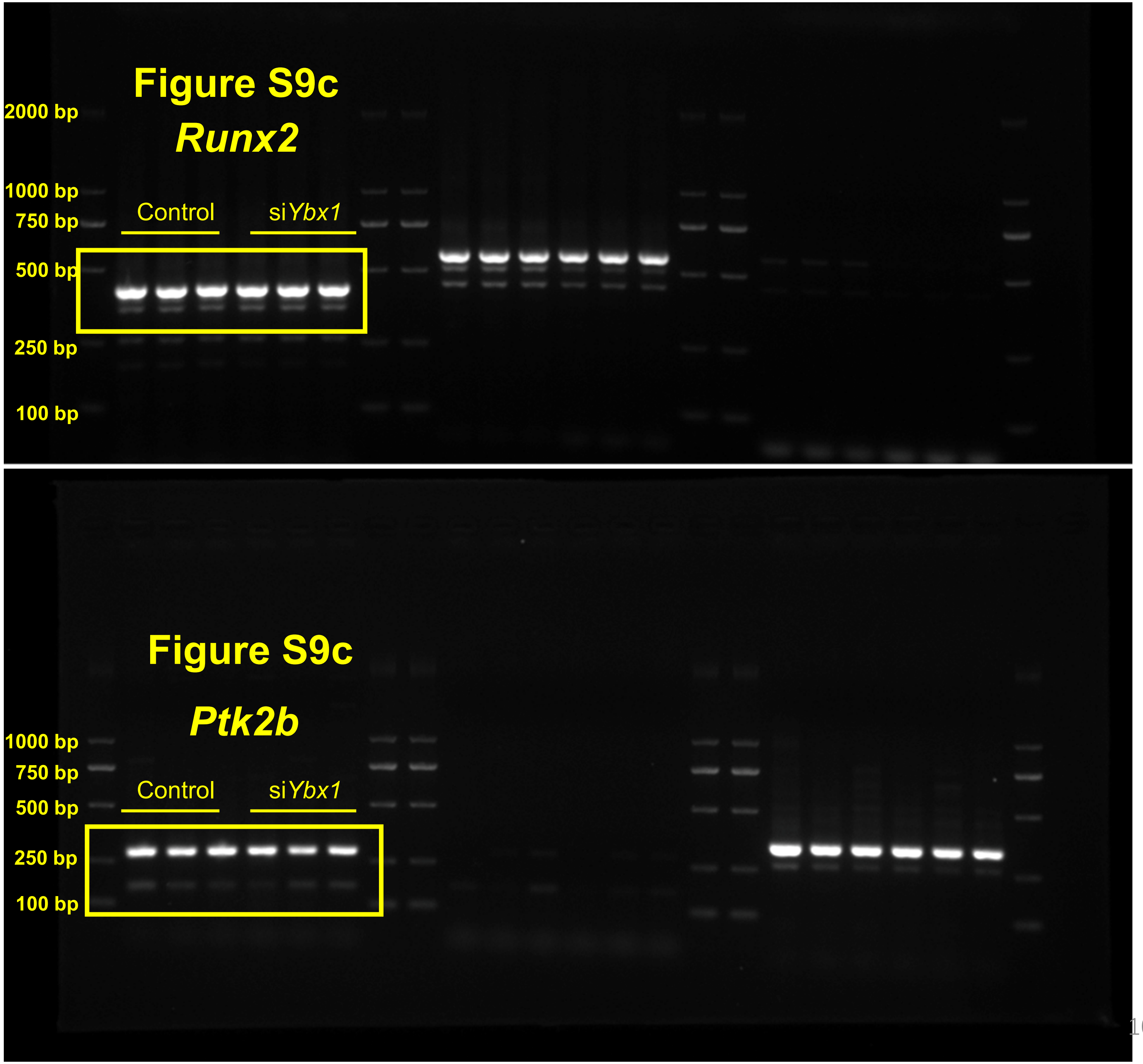

Unedited blot images

Figure 5d and S10a

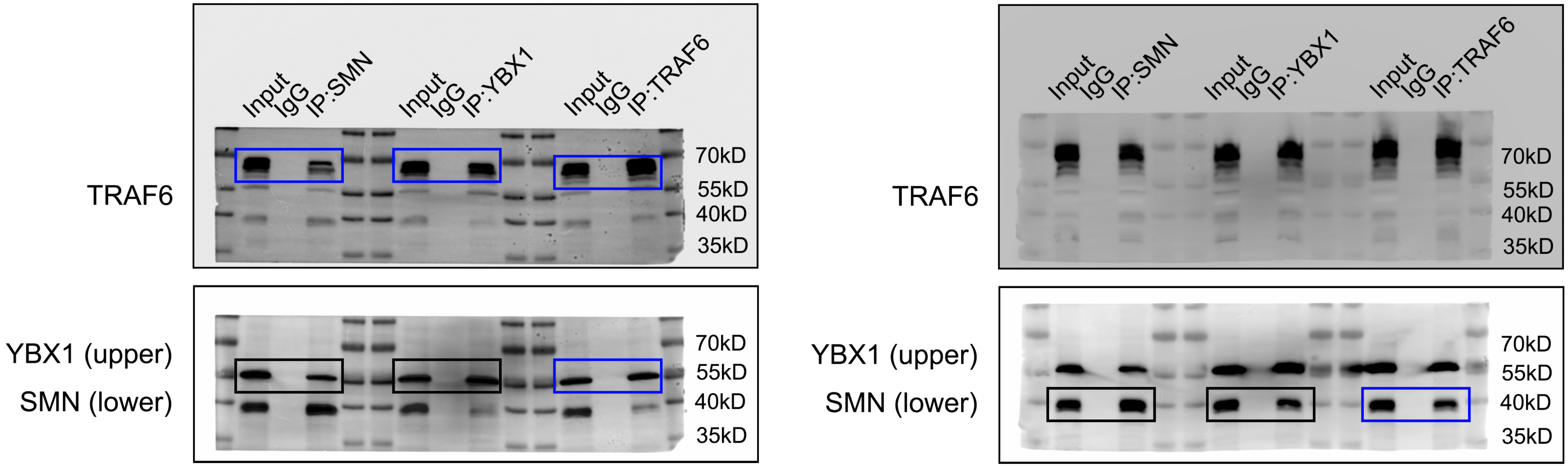

Black box indicates the region used for Figure 5d in the manuscript.

Blue box indicates the region used for Figure S10a in the manuscript.

The left and right panels represent two independent biological replicates.

The upper and lower panels on each side were obtained from the same PVDF membrane by sequential antibody stripping and reprobing.

SMN and YBX1 were detected simultaneously using a mixed primary antibody solution.

Figure 5f and 5g

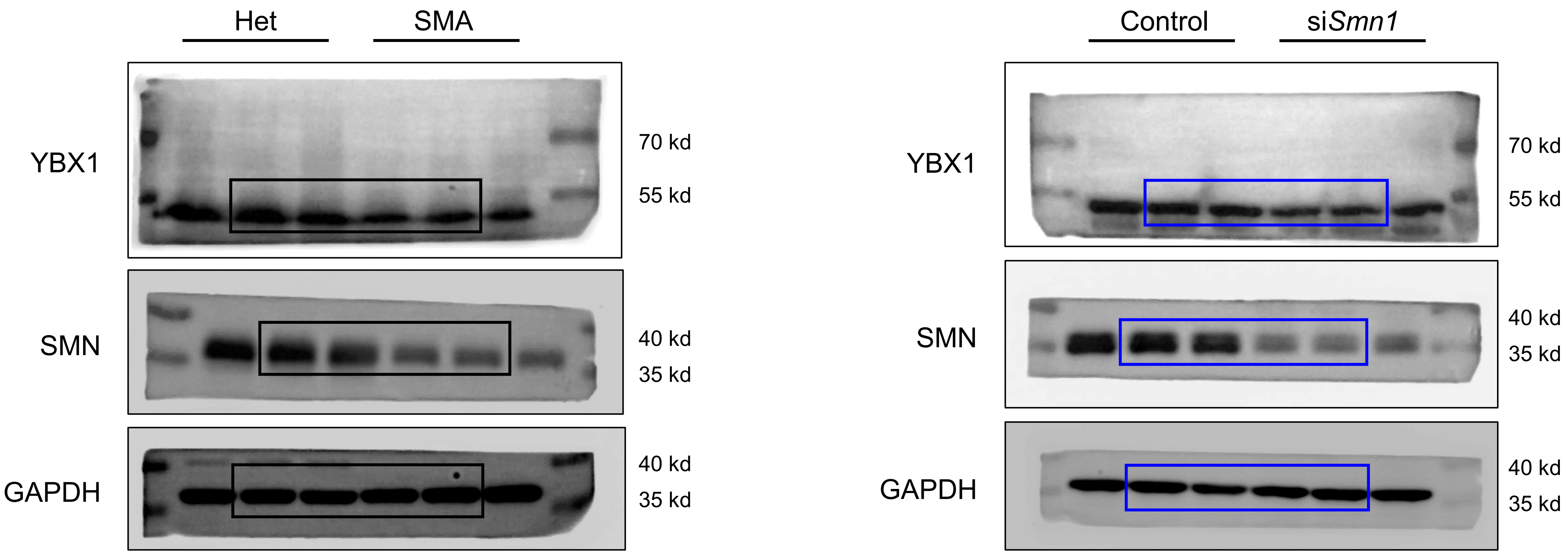

Black box indicates the region used for Figure 5f in the manuscript.

Blue box indicates the region used for Figure 5g in the manuscript.

Figure 5h

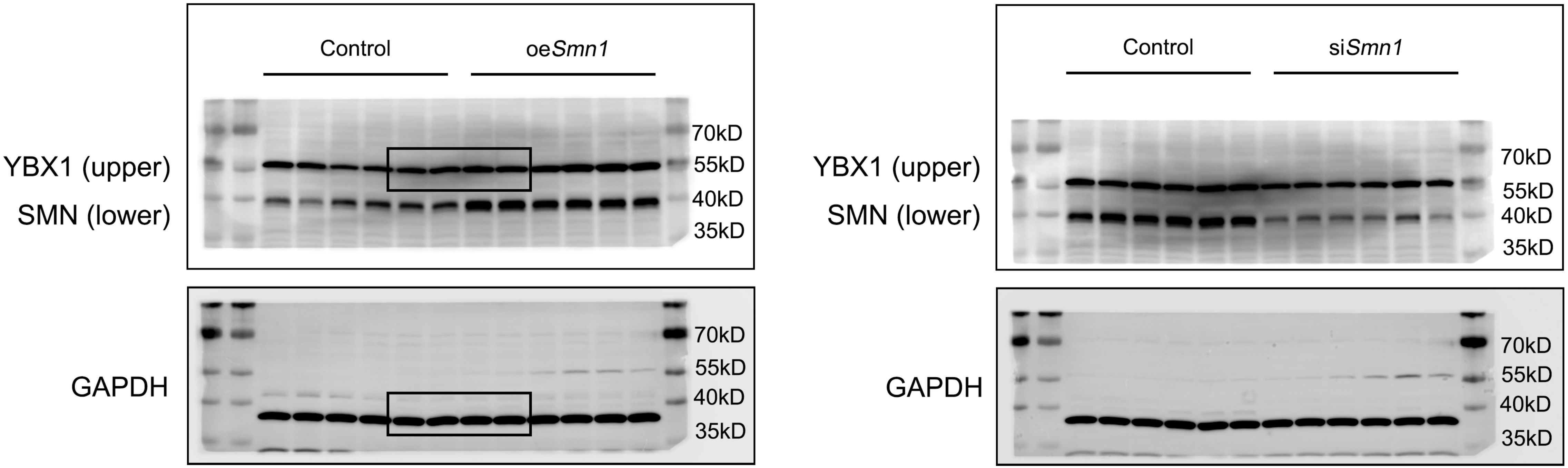

Black box indicates the region used for Figure 5h in the manuscript.

The upper and lower panels on each side were obtained from the same PVDF membrane by sequential antibody stripping and reprobing.

SMN and YBX1 were detected simultaneously using a mixed primary antibody solution.

The YBX1 and SMN band on right panels was not displayed in the final figures but served as a replicate in the quantification of Figure 5g.

Figure 6c

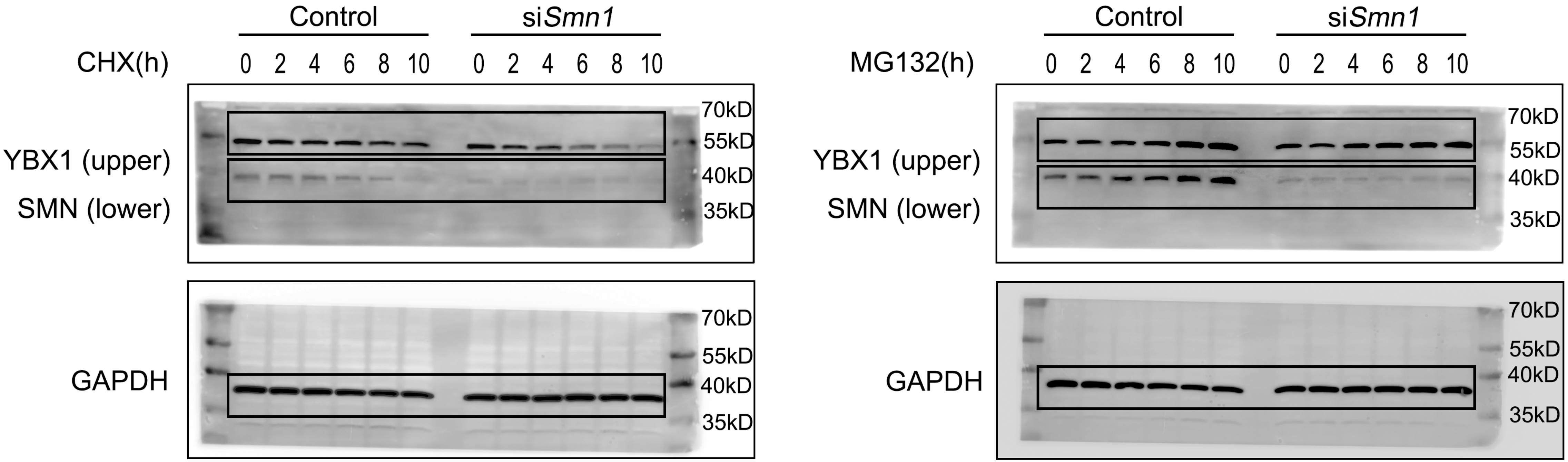

Black box indicates the region used for Figure 6c in the manuscript.

The upper and lower panels on each side were obtained from the same PVDF membrane by sequential antibody stripping and reprobing.

SMN and YBX1 were detected simultaneously using a mixed primary antibody solution.

Figure 6e and 6f

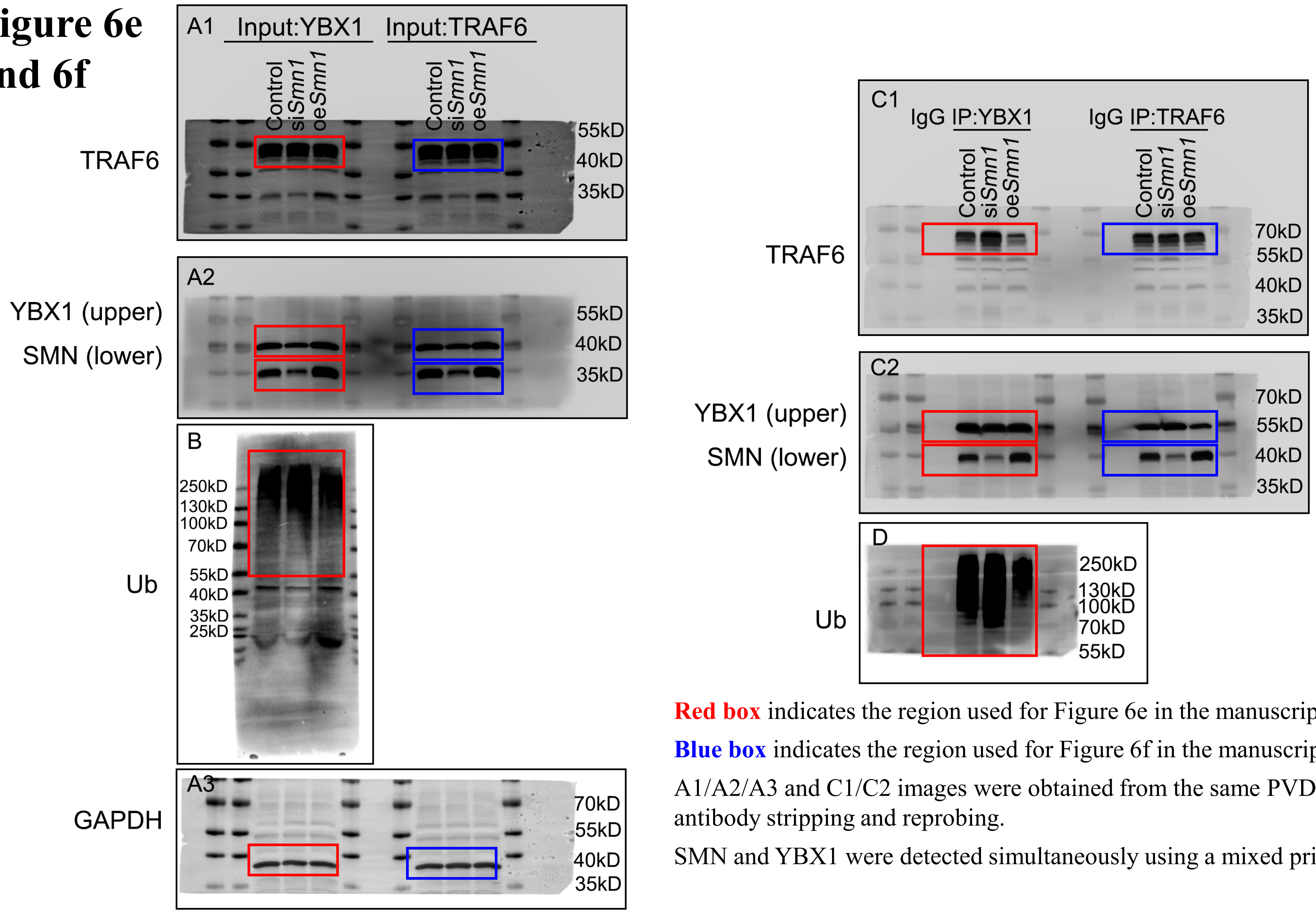

Red box indicates the region used for Figure 6e in the manuscript.

Blue box indicates the region used for Figure 6f in the manuscript.

A1/A2/A3 and C1/C2 images were obtained from the same PVDF membrane by sequential antibody stripping and reprobing.

SMN and YBX1 were detected simultaneously using a mixed primary antibody solution.

Figure 6g

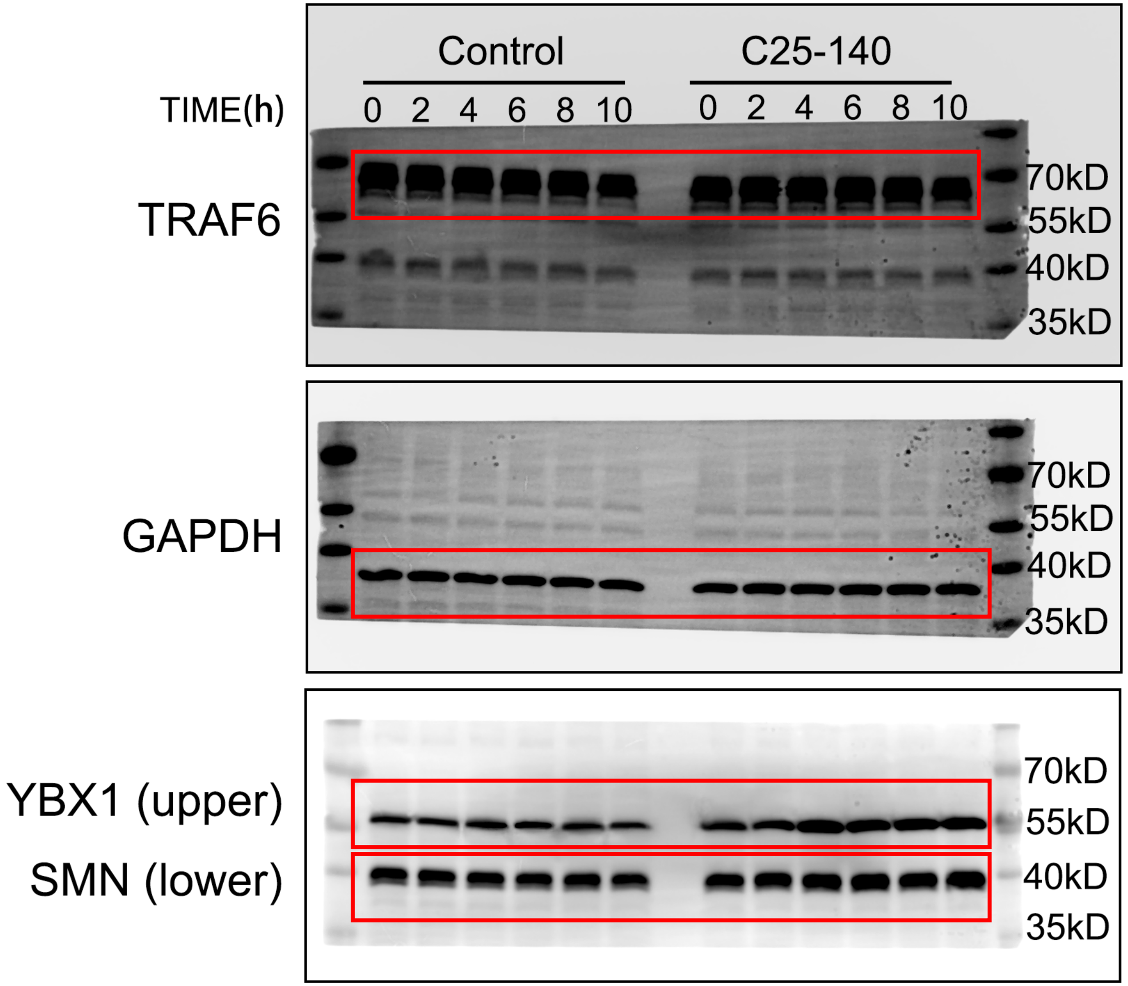

Red box indicates the region used for Figure 6g in the manuscript.

The first two indicators were obtained from the same PVDF membrane by sequential antibody stripping and reprobing.

SMN and YBX1 were detected simultaneously using a mixed primary antibody solution.

Figure S2d

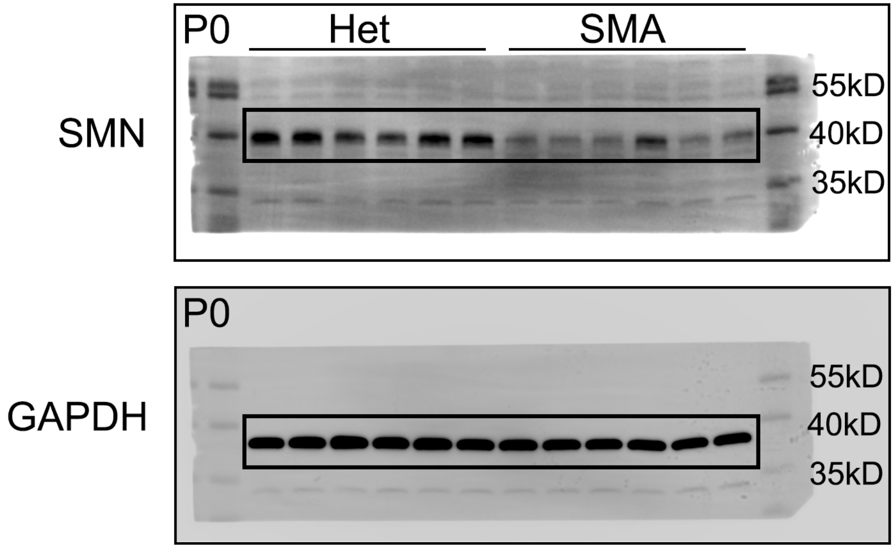

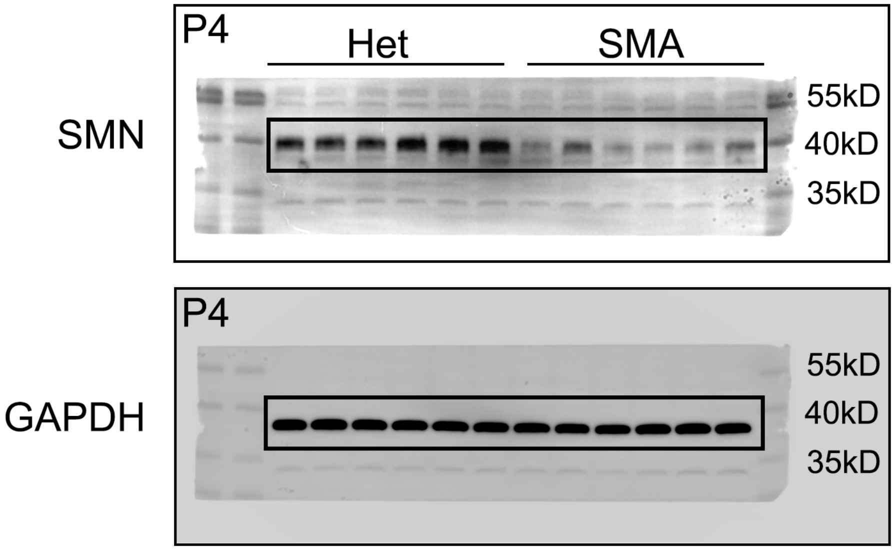

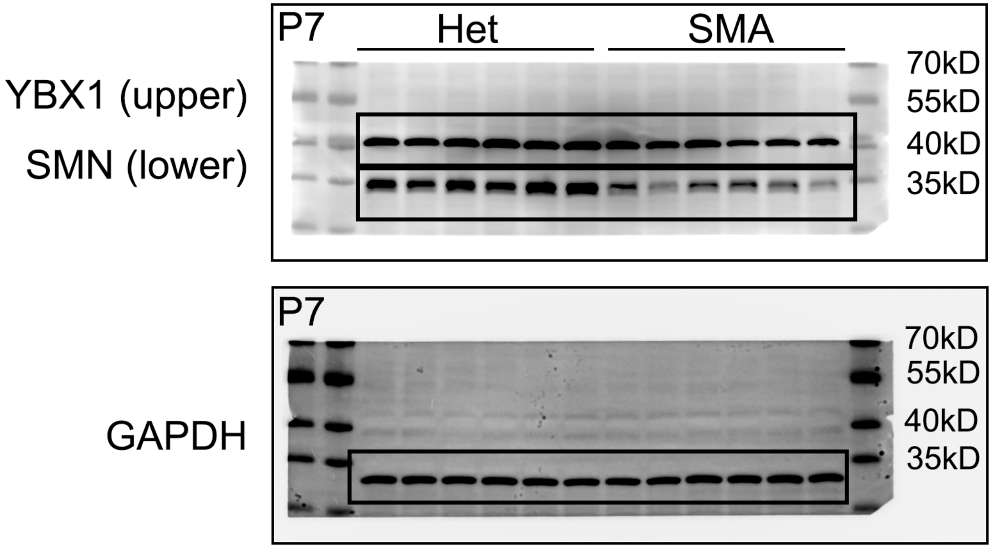

Black box indicates the region used for Figure S2d in the manuscript.

All results at each time point were obtained from the same PVDF membrane by sequential antibody stripping and reprobing.

For P7, SMN and YBX1 were detected simultaneously using a mixed primary antibody solution.

The YBX1 band for P7 was not displayed in the final figures but served as a replicate in the quantification of Figure 5f.

Figure S4b

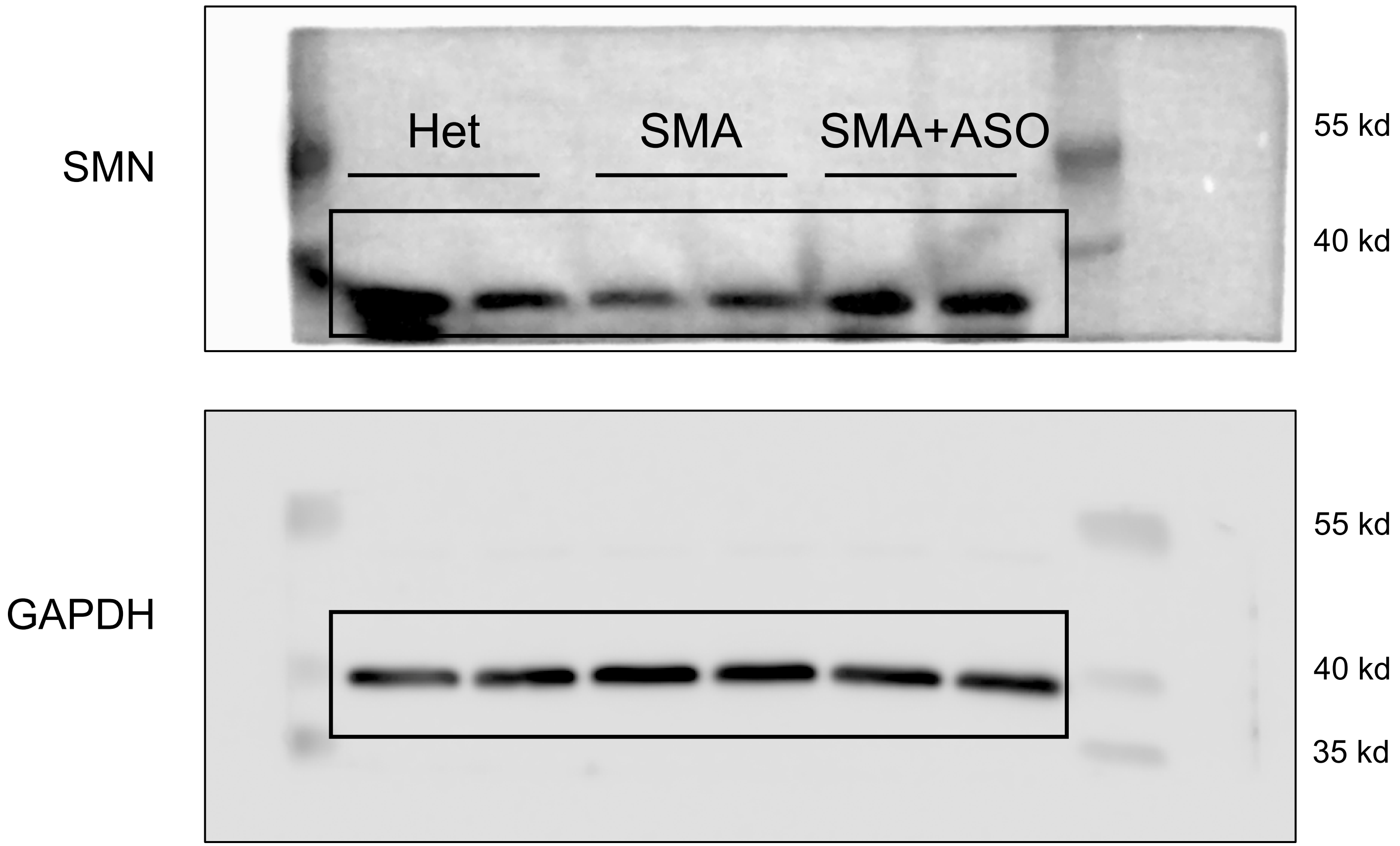

Black box indicates the region used for Figure S4b in the manuscript.

Unedited blot images (excluded)

These blots were shown in the original submission but later excluded in the revised version due to insufficient image clarity. Original unedited images are provided here for transparency.

Appeared in the first submission
Figure 5d and 6h

Black box indicates the region appeared in Figure 5d of the first submission

Red box indicates the region appeared in Figure 6h of the first submission

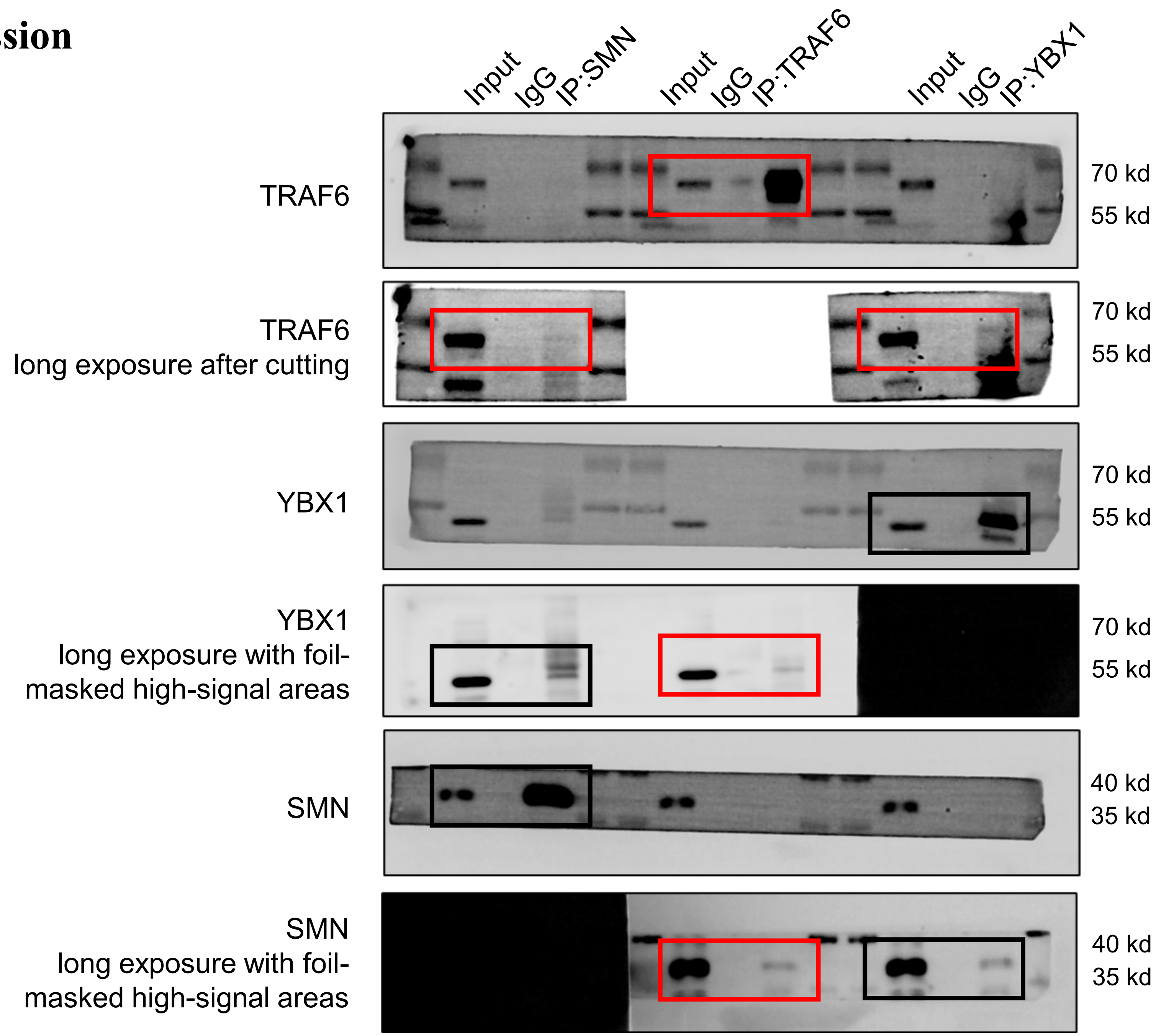

Appeared in the first submission
Figure 6c

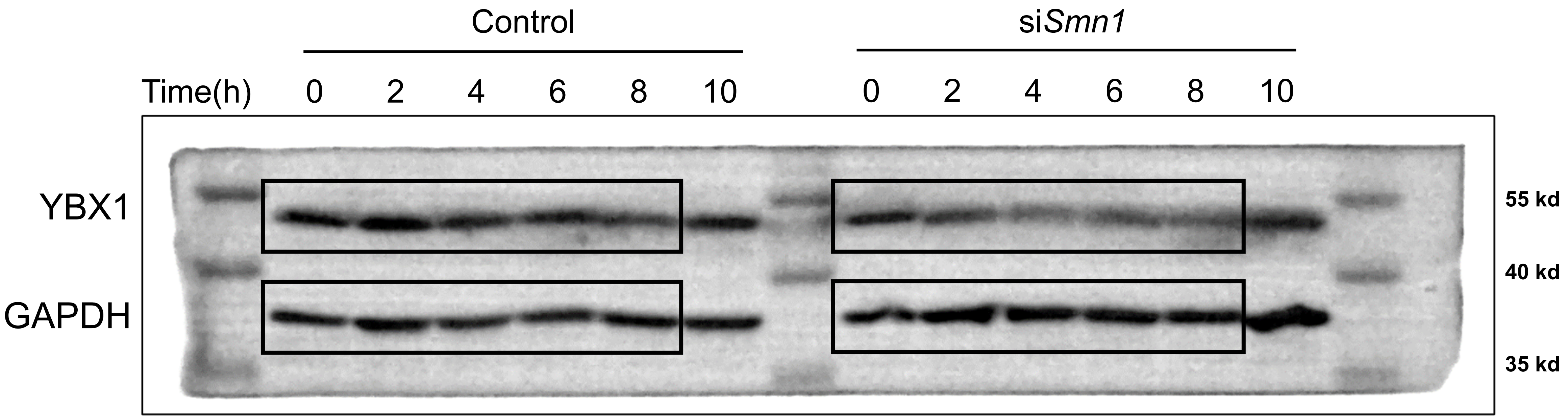

Black box indicates the region appeared in Figure 6c of the first submission.

Appeared in the second submission
Figure 6c

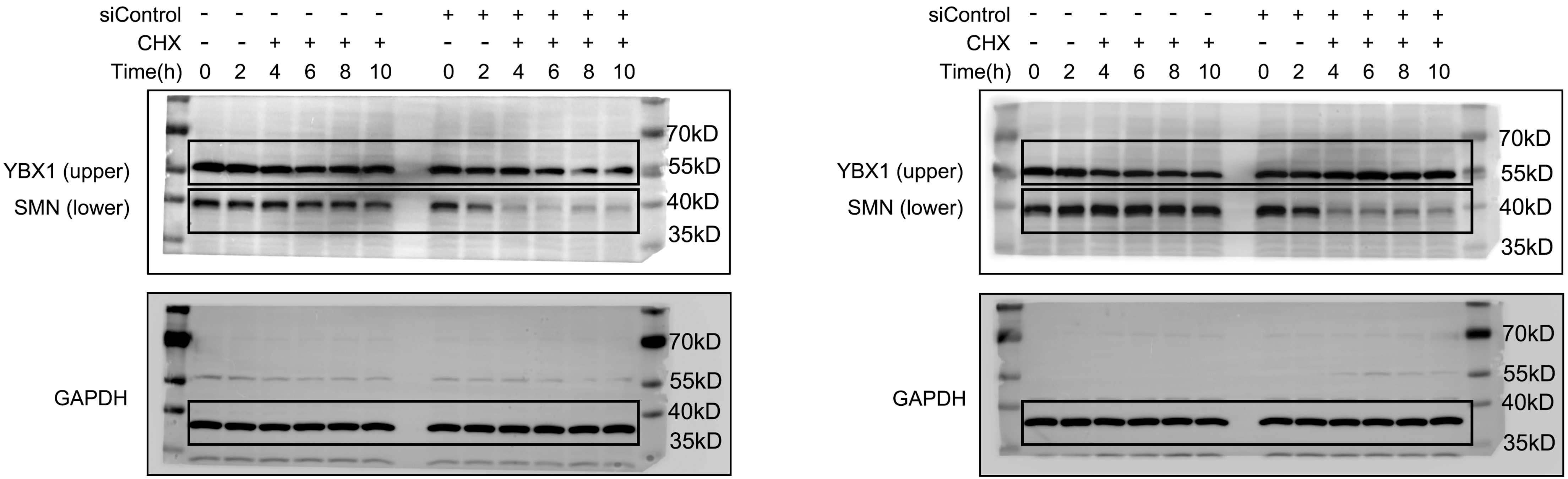

Black box indicates the region used for Figure 6c in the manuscript.

The upper and lower panels on each side were obtained from the same PVDF membrane by sequential antibody stripping and reprobing.

SMN and YBX1 were detected simultaneously using a mixed primary antibody solution.

Appeared in the first submission
Figure 6d and 6i

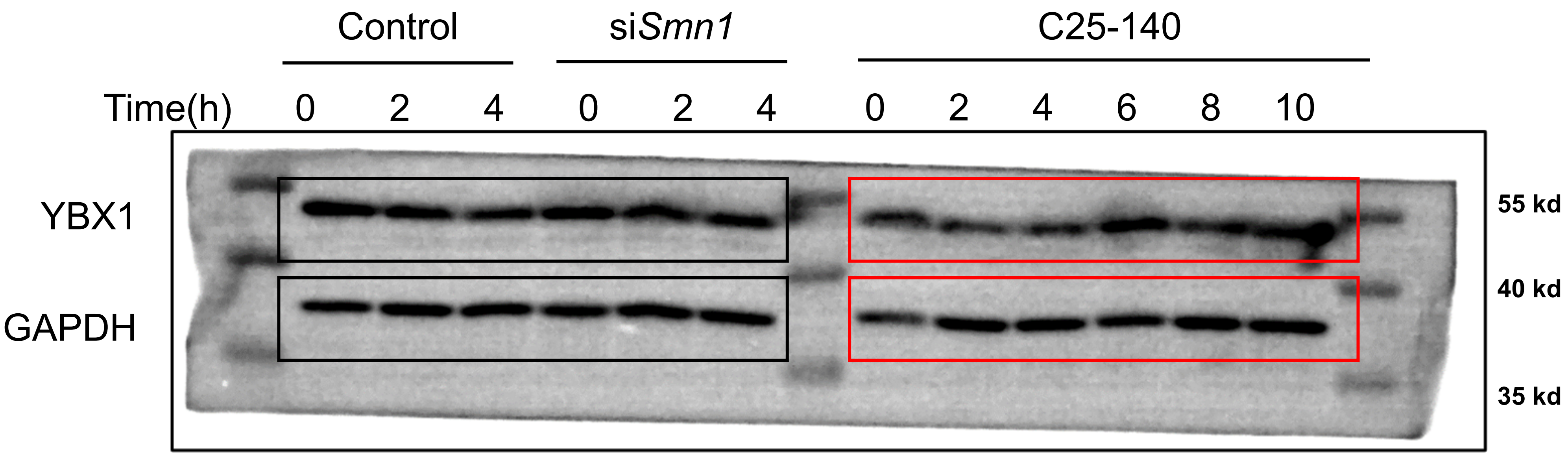

Black box indicates the region appeared in Figure 6d of the first submission

Red box indicates the region appeared in Figure 6i of the first submission

Appeared in the first submission
Figure 6e

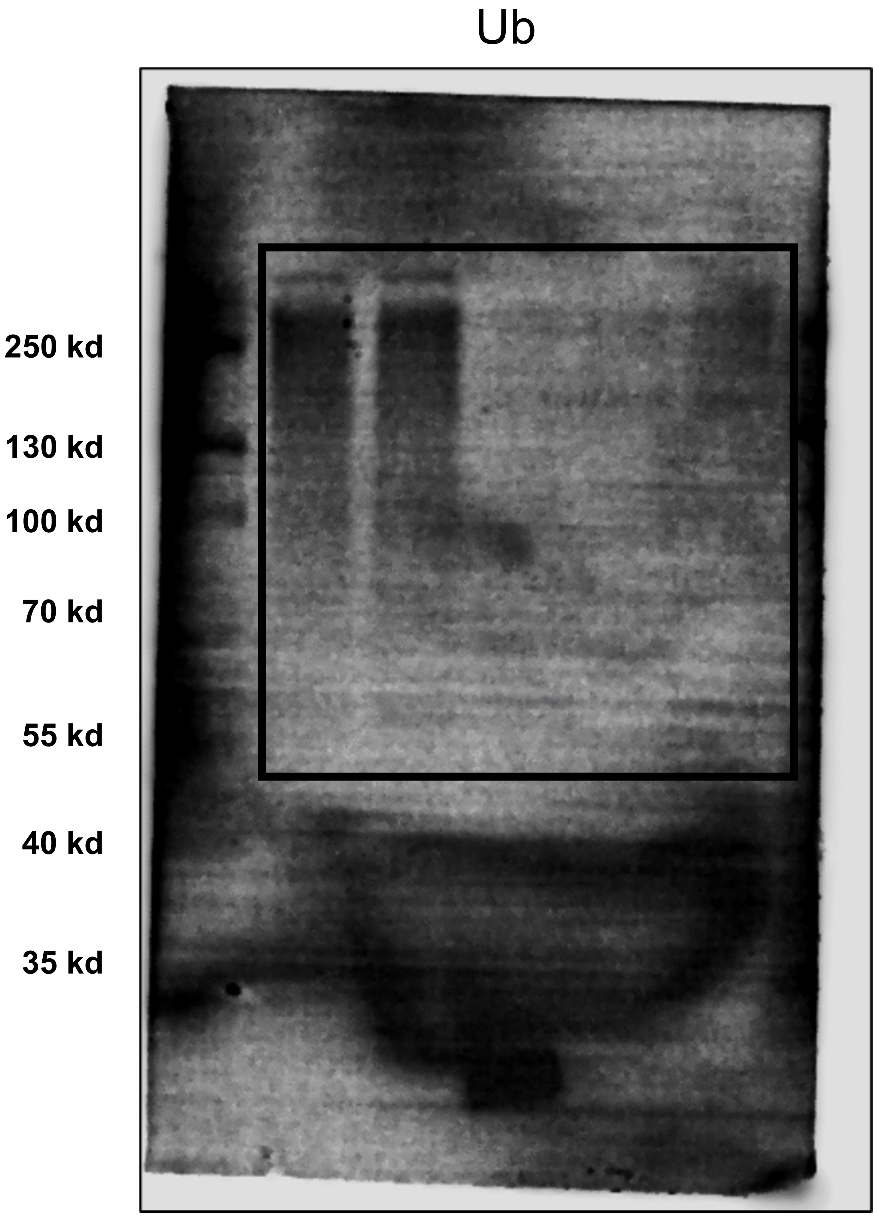

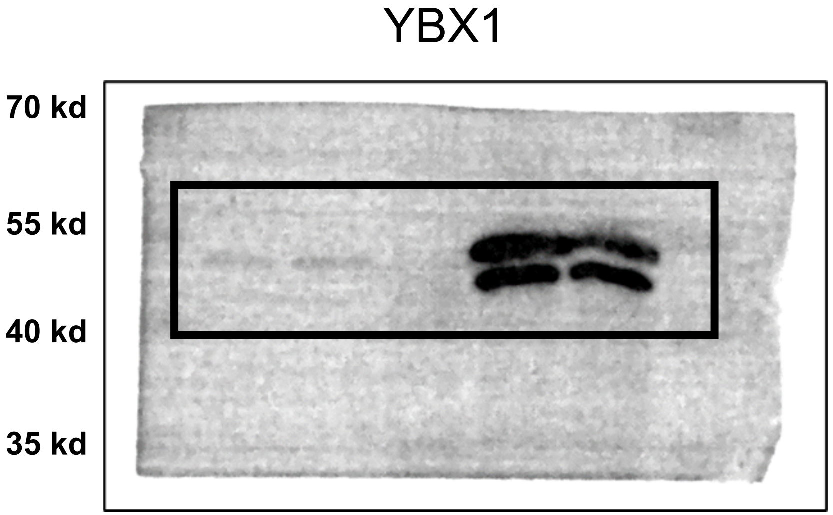

Black box indicates the region appeared in Figure 6e of the first submission.
